# Exploring the Antitubercular Activity of Anthranilic Acid Derivatives: From MabA (FabG1) Inhibition to Intrabacterial Acidification

**DOI:** 10.3390/ph16030335

**Published:** 2023-02-22

**Authors:** Léo Faïon, Kamel Djaout, Catalin Pintiala, Catherine Piveteau, Florence Leroux, Alexandre Biela, Stéphanie Slupek, Rudy Antoine, Monika Záhorszká, Francois-Xavier Cantrelle, Xavier Hanoulle, Jana Korduláková, Benoit Deprez, Nicolas Willand, Alain R. Baulard, Marion Flipo

**Affiliations:** 1 Univ. Lille, Inserm, Institut Pasteur de Lille, U1177—Drugs and Molecules for Living Systems, F-59000 Lille, France; 2 Univ. Lille, CNRS, Inserm, CHU Lille, Institut Pasteur de Lille, U1019—UMR 9017—CIIL—Center for Infection and Immunity of Lille, F-59000 Lille, France; 3 Univ. Lille, CNRS, Inserm, CHU Lille, Institut Pasteur Lille, US 41—UAR 2014—PLBS, F-59000 Lille, France; 4 Department of Biochemistry, Faculty of Natural Sciences, Comenius University in Bratislava, Mlynská Dolina, Ilkovičova 6, 842 15 Bratislava, Slovakia; 5 Univ. Lille, Inserm, CHU Lille, Institut Pasteur de Lille, U1167—RID-AGE—Risk Factors and Molecular Determinants of Aging-Related Diseases, F-59000 Lille, France; 6 CNRS, EMR9002—Integrative Structural Biology, F-59000 Lille, France

**Keywords:** MabA inhibitors, anthranilic acid, FabG1, tuberculosis, mycolic acids

## Abstract

*Mycobacterium tuberculosis*, the pathogen that causes tuberculosis, is responsible for the death of 1.5 million people each year and the number of bacteria resistant to the standard regimen is constantly increasing. This highlights the need to discover molecules that act on new *M. tuberculosis* targets. Mycolic acids, which are very long-chain fatty acids essential for *M. tuberculosis* viability, are synthesized by two types of fatty acid synthase (FAS) systems. MabA (FabG1) is an essential enzyme belonging to the FAS-II cycle. We have recently reported the discovery of anthranilic acids as MabA inhibitors. Here, the structure–activity relationships around the anthranilic acid core, the binding of a fluorinated analog to MabA by NMR experiments, the physico-chemical properties and the antimycobacterial activity of these inhibitors were explored. Further investigation of the mechanism of action *in bacterio* showed that these compounds affect other targets than MabA in mycobacterial cells and that their antituberculous activity is due to the carboxylic acid moiety which induces intrabacterial acidification.

## 1. Introduction

Tuberculosis (TB), an infectious disease caused by *Mycobacterium tuberculosis*, is one of the leading causes of death from a single infectious agent [1]. The World Health Organization (WHO) estimates that in 2021, 10.6 million people developed the disease and 1.6 million died from TB including 187,000 deaths among HIV-positive people [2]. In addition, the number of bacteria resistant to anti-TB drugs is constantly increasing and in 2021, around 450,000 people developed rifampicin-resistant TB, of which 78% had multidrug-resistant TB (MDR-TB) [2]. 

In the last 10 years, three new drugs, bedaquiline [3], delamanid [4] and pretomanid [5] have been approved for the treatment of resistant TB. Nevertheless, treatment success rates remain insufficient, particularly in patients with MDR- and XDR-TB (extensively drug-resistant) [6], and bacteria resistance to these new drugs has already been reported in many countries, warning that new compounds acting on unexploited targets are needed.

Mycolic acids, a key component of the bacterial cell wall, are essential for *M. tuberculosis* viability [7]. Characterized as very long-chain α-alkyl-β-hydroxy fatty acids (C70–C90) and composed of a C24–C26 saturated α-chain and a meromycolate chain up to C56, their biosynthesis is carried out by two types of fatty acid synthase (FAS) systems, FAS-I and FAS-II [8,9]. FAS-I synthesizes short-chain fatty acids with two different chain lengths: C24–C26 which correspond to the α-branch in mycolic acids and C16–C18 which are then elongated by FAS-II to produce the meromycolate chain [10]. FAS-II is composed of four types of enzymes, which act successively to ensure fatty acid elongation. Several cycles catalyzed by MabA, HadAB/BC, InhA and KasA/B lead to very long meromycolic acid chains (C42–C56) which will produce mycolic acids [11]. These enzymes have been shown to be essential for bacterial growth [12,13,14,15] and several antibiotics target InhA, HadAB and KasA/B [16,17,18,19]. However, MabA (FabG1) was the only enzyme of the FAS-II cycle without a specific inhibitor. We have recently reported the discovery of the first MabA inhibitors through a fragment-based screening approach [20].

**Figure 1:**
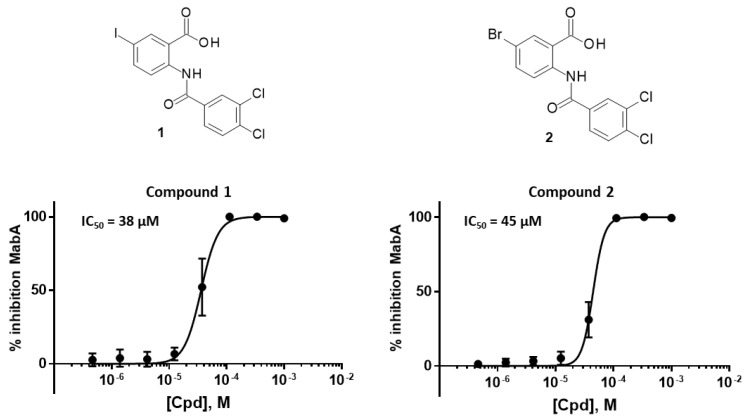
Structures and dose–response curves of compounds **1** and **2**. The Y-axis represents the inhibition of the transformation of acetoacetyl-CoA in HBCoA by MabA measured by mass spectrometry [20].

MabA catalyzes the NADPH-specific reduction of β-ketoacyl derivatives into β-hydroxyacyl derivatives [21]. In order to discover MabA inhibitors, we developed a new LC-MS/MS-based enzymatic assay in microplates [20]. In this assay, MabA catalyzes the reduction of acetoacetyl-CoA into hydroxybutyryl-CoA (HBCoA) using NADPH as a cofactor and we detect the formation of HBCoA by mass spectrometry. The screening of a 1280-fragment library led to the selection of an anthranilic acid series and early structure-activity relationships studies led to the identification of compound **1**. This inhibitor, composed of a 3,4-dichlorobenzoyl motif linked to the nitrogen of the anthranilic core and an iodine atom in position 5 of the phenyl ring, inhibits MabA with an IC_50_ of 38 ± 6 µM (Figure 1) [20]. The direct brominated analog (compound **2**) displayed a similar potency (IC_50_ = 45 ± 6 µM).

With the aim of increasing the potency of our compounds, the structure–activity relationships (SAR) around the anthranilic acid core were further explored. The SAR study includes the bioisosteric replacement of the amide and carboxylic acid functions as well as the modification of the iodophenyl ring. The direct binding of a fluorinated anthranilic acid analog to MabA was confirmed by ^19^F ligand-observed NMR experiments. Physico-chemical properties and antimycobacterial activity of five chemically diverse compounds were then evaluated. Further study on how these compounds affect bacterial growth revealed that they impact targets other than MabA in mycobacterial cells and that their antituberculous activity is due to the carboxylic acid moiety which causes acidification inside the bacteria.

## 2. Results and Discussion

### 2.1. Chemistry

#### 2.1.1. Study of the Modification of the Amide Bond

In the iodine series, the first modifications introduced were the methylation of the nitrogen of the amide linker leading to compound **3** and the replacement of the amide bond by a sulfonamide leading to compound **4**. In the bromine series, the retro amide (compound **5**) and thioamide (compound **6**) analogs were synthesized. The bioisosteric replacement of the amide by a 1,2,4-oxadiazole ring (compound **7**) was also tested [22].

**Scheme 1:**
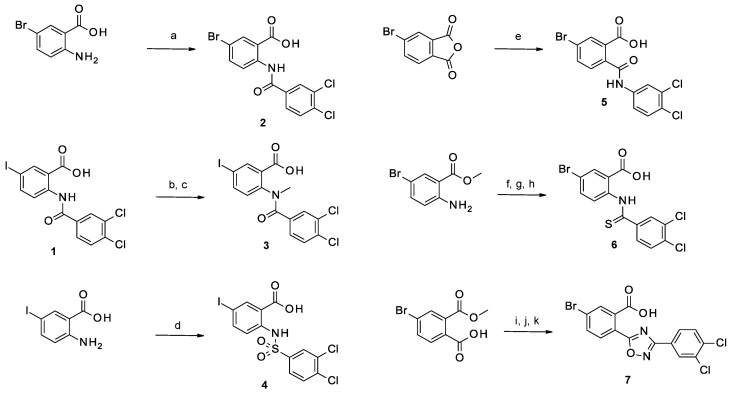
Synthesis of compounds **2**–**7**. Reagents and conditions: (a) Na_2_CO_3_ (2.36 eq), isopropoxycarbonyl 3,4-dichlorobenzoate (1.5 eq), THF/H_2_O (1/1), RT, 3 h; (b) CH_3_I (3.2 eq), Cs_2_CO_3_ (3.5 eq), anh. THF/DMF (3/1), RT, overnight; (c) LiOH (3 eq), H_2_O, RT, 5 h; (d) 3,4-dichlorobenzenesulfonyl chloride (1 eq), Na_2_CO_3_ (1 eq), H_2_O, RT, 30 min; (e) 3,4-dichloroaniline (1 eq), DIEA (2.2 eq), anh. DMF, RT, overnight; (f) 3,4-dichlorobenzoyl chloride (1 eq), anh. pyridine, RT, 1 h; (g) Lawesson’s reagent (2 eq), anh. toluene, argon, 110 °C, 4 days; (h) NaOH (4 eq), MeOH, H_2_O, reflux, overnight; (i) oxalyl chloride (1.2 eq), DMF, DCM, RT, 1 h; (j) 3,4-dichloro-*N*′-hydroxy-benzamidine, pyridine, 100 °C, 4.5 h (k) NaOH (4 eq), MeOH/H_2_O (20/1), 65 °C, 1 h.

Following the methodology described previously [20], the coupling between 2-amino-5-bromo benzoic acid and isopropoxycarbonyl 3,4-dichlorobenzoate led to compound **2** (Scheme 1). Compound **3** was obtained by alkylation of compound **1** with an excess of iodomethane, which led to the alkylation of both the amide and the carboxylic acid. The hydrolysis of the methyl ester was then performed using LiOH. Compound **4** was synthesized by coupling 2-amino-5-iodobenzoic acid with 3,4-dichlorobenzenesulfonyl chloride. The reaction of 4-bromo-phthalic anhydride with 3-4-dichloroaniline led to compound **5** and its regioisomer which were separated by preparative HPLC. Thioamide **6** was synthesized following a three-step procedure: firstly, methyl-2-amino-5-bromobenzoate was coupled with 3,4-dichlorobenzoyl chloride to give the corresponding amide, followed by thionation using Lawesson’s reagent, and finally, the desired compound **6** was obtained by hydrolysis of the methyl ester using NaOH. The 1,2,4-oxadiazole analog (compound **7**) was also obtained after three chemical steps. Firstly, 4-bromo-2-methoxycarbonyl-benzoic acid was functionalized in the corresponding acid chloride using oxalyl chloride, which was then coupled with 3,4-dichloro-*N*′-hydroxy-benzamidine. The 1,2,4-oxadiazole ring formation was obtained at 100 °C and the hydrolysis of the methyl ester using NaOH led to the corresponding acid (compound **7**).

#### 2.1.2. Study of the Modification of the Carboxylic Acid Moiety

**Scheme 2:**
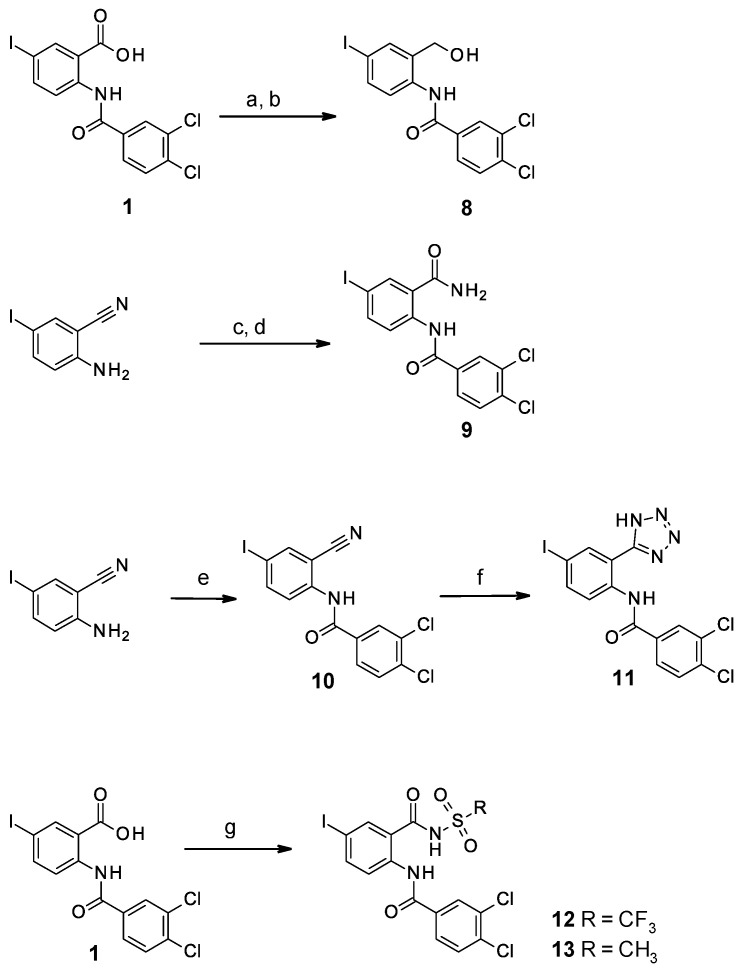
Synthesis of compounds **8**–**13**. Reagents and conditions: (a) DCC (1.02 eq), *N*-hydroxysuccinimide (1.07 eq), EtOAc, 0 °C to RT, overnight; (b) NaBH_4_ (3.1 eq), THF/H_2_O (10/1), RT, 1.5 h; (c) H_2_SO_4_ (solvent), RT, 72 h; (d) Na_2_CO_3_ (2 eq), isopropoxycarbonyl 3,4-dichlorobenzoate (1.5 eq), THF/H_2_O (1/1), RT, 72 h; (e) 3,4-dichlorobenzoyl chloride (1.5 eq), pyridine, RT, 1 h; (f) NaN_3_ (1 eq), ZnBr_2_ (1 eq), DMF, 130 °C, 2 h; (g) trifluoromethanesulfonamide or methanesulfonamide (2 eq), CDI (2 eq), DBU (3 eq), anh. THF, RT, 3 h.

We first replaced the carboxylic acid moiety with a primary alcohol. Compound **8** was obtained from the corresponding carboxylic acid (compound **1**) which was coupled with *N*-hydroxysuccinimide using DCC (dicyclohexylcarbodiimide) and then reduced by sodium borohydride (Scheme 2). The synthesis of the primary carboxamide analog (compound **9**) proceeded in two steps starting from 2-amino-5-iodo-benzonitrile. Firstly, the nitrile was hydrolyzed under acidic conditions into the primary amide [23] and then the aniline was coupled with isopropoxycarbonyl 3,4-dichlorobenzoate to give the desired compound **9**. The nitrile analog (compound **10**) was obtained from the coupling of 2-cyano-4-iodoaniline with 3,4-dichlorobenzoyl chloride. The bioisosteric replacement of the carboxylic acid by a tetrazole (compound **11**) was achieved starting from compound **10**, by the addition of sodium azide. Finally, acylsulfonamides (compounds **12**–**13**) were obtained by coupling the carboxylic acid **1** with the respective sulfonamides using CDI (carbonyldiimidazole) and DBU (1,8-Diazabicyclo [5.4.0]undec-7-ene) [23].

#### 2.1.3. Study of the Modification of the Phenyl Ring

The substitution of the phenyl ring and the introduction of new aromatic rings were explored. Since an increase in activity correlated with an increase in halogen size has been previously observed in the Boc-anthranilic acid series [20], we assumed that a bulky and hydrophobic substituent was preferable for activity. Firstly, we moved the bromine atom to position 4 on the phenyl ring of the anthranilic acid core to obtain compound **14**. We then replaced the bromine atom with a hydrogen atom (compound **15**) to confirm the loss of activity. The introduction of alkyl substituents was then explored. As the ethynyl group was described as a bioisostere of the iodine atom due to their similar molecular electrostatic potentials [24], an ethynyl group was introduced in position 4 (compound **17**) as well as a cyclopropylethynyl in position 5 (compound **16**). To complete this exploration, a pyrazole was introduced in position 4 (compound **18**). Finally, the introduction of a naphthalene (compound **19**) and a quinoline (compound **20**) as a replacement for the phenyl core was also investigated.

**Scheme 3:**
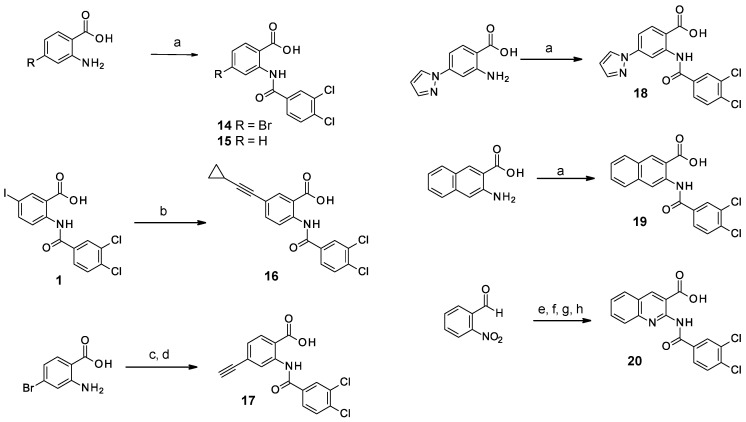
Synthesis of compounds **14**–**20**. Reagents and conditions: (a) Na_2_CO_3_ (2 eq), isopropoxycarbonyl 3,4-dichlorobenzoate (1.5 eq), THF/H_2_O (1/1), RT, 2 h to overnight; (b) ethynylcyclopropane (2 eq) PdCl_2_(PPh_3_)_2_ (6%), TBAI (1.5 eq), CuI (0.2 eq), TEA (16.9 eq), anh. acetonitrile, RT, 1.5 h, then 60 °C 30 min; (c) ethynyl(trimethyl)silane (6 eq), triphenyl phosphine (0.1 eq), CuI (0.1 eq), PdCl_2_(PPh_3_)_2_ (0.1 eq), TEA (46.5 eq), anh. THF, argon, 80 °C, 2 h; (d) Na_2_CO_3_ (2 eq), isopropoxycarbonyl 3,4-dichlorobenzoate (1.5 eq), THF/H_2_O, RT, 4 h; (e) ethyl 2-cyanoacetate (1 eq), piperidine (1.1 eq), ethanol, RT, 1.5 h; (f) Fe (3 eq), acetic acid, 80 °C, 1.5 h; (g) 3,4-dichlorobenzoyl chloride (3.5 eq), pyridine, 100 °C under microwave irradiation, 30 min; (h), NaOH (4 eq), H_2_O, EtOH, RT, 1 h.

Amides **14** and **15** were obtained as described above from commercial anilines (Scheme 3). Alkynes **16** and **17** were obtained using a Sonogashira coupling from iodo or bromo derivatives. Compounds **18** and **19** were isolated after the coupling of the appropriate anilines with isopropoxycarbonyl 3,4-dichlorobenzoate. Finally, 2-nitrobenzaldehyde was used in a Knoevenagel condensation with ethyl 2-cyanoacetate, then reduction of the nitro group followed by an intramolecular cyclization led to ethyl 2-aminoquinoline-3-carboxylate. Coupling with 3,4-dichlorobenzoyl chloride and hydrolysis of the ethyl ester led to the desired compound **20**.

### 2.2. Biological Results

All compounds were evaluated for their inhibitory activity on MabA using our LC-MS/MS-based enzymatic assay. The potency of the compounds is reported as the concentration inhibiting 50% of the transformation of acetoacetyl-CoA in HBCoA in the presence of NADPH.

#### 2.2.1. Modifications of the Amide Bond

In the iodine series, the replacement of the amide function with a sulfonamide function (compound **4**) led to a compound as potent as the reference compound **1** while methylation of the amide function (compound **3**) decreased the potency by more than five times (Table 1). In the bromine series, the replacement of the amide function with a thioamide function was tolerated (compound **6**) while the introduction of a reverse amide or a 1,2,4-oxadiazole (compounds **5** and **7**) was less favorable.

The exploration of the replacement of the amide function did not lead to more potent analogs; therefore, all the following modifications were performed in the amide series.

#### 2.2.2. Modifications of the Carboxylic Acid Moiety

The replacement of the carboxylic acid with alcohol (compound **8**), a carboxamide (compound **9**) or a nitrile (compound **10**) was deleterious for activity (IC_50_ > 333 µM). On the contrary, its replacement by bioisosteres such as a tetrazole (compound **11**) or acylsulfonamides (compounds **12**-**13**) was well tolerated and led to compounds with IC_50_ between 24 and 35 µM (Table 2). Overall, these SARs show that the presence of an acid function is required to inhibit MabA in vitro.

#### 2.2.3. Modifications of the Phenyl Ring

Removal of the halogen atom on the phenyl ring (compound **15**) led to an inactive compound (IC_50_ > 1000 µM). The introduction of a bromine atom in position 4 of the phenyl ring (compound **14**) instead of position 5 (compound **2**) led to a compound with similar activity suggesting that both positions are tolerated for the introduction of substituents (Table 3). This observation was confirmed by the introduction of a fused phenyl ring (compound **19**), as a similar activity to that of the halogenated compounds was observed for this naphthyl analog. Replacing the naphthyl ring with a quinoline ring (compound **20**) resulted in a 2-fold reduction in the potency (IC_50_ = 76 µM). The replacement of the iodine atom in position 5 of the phenyl ring with a cyclopropyl-substituted alkyne (compound **16**) led to a one-third reduction in potency (IC_50_ = 60 µM) compared to the reference compound whereas the introduction of an unsubstituted alkyne in position 4 (compound **17**) led to a 2-fold decrease in potency (IC_50_ = 77 µM). Interestingly, the introduction of a pyrazole in position 4 of the phenyl ring (compound **18**) improved the activity by a factor of 1.6 (IC_50_ = 23 µM).

These SARs show that the introduction of a bulky substituent in position 4 or 5 of the phenyl ring is important for activity. This suggests that a new hydrophobic contact with the binding pocket of MabA may have been created. The replacement of the halogen atom by ethynyl groups, which are described as bioisosteres of the iodine atom [24], is well tolerated and supports this hypothesis.

#### 2.2.4. Evaluation of the Interaction of the Fluorinated Compound **12** with MabA by NMR

**Figure 2:**
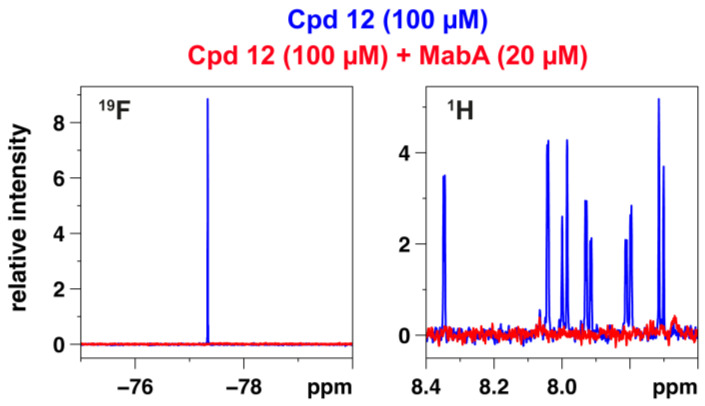
Superimposition of 1D ^19^F (**left panel**) and ^1^H (**right panel**) NMR spectra of compound **12** at 100 µM alone (in blue) and in the presence of MabA at 20 µM (in red).

^19^F-NMR experiments have already been used to confirm the binding of fluorinated inhibitors to MabA [20]. Ligand-observed NMR techniques can be applied to detect the weak binding of small molecules as measured by the reduction in fluorine or proton signal intensity [25,26]. Compound **12** bearing a trifluoromethyl group was selected for NMR experiments using a 600 MHz spectrometer equipped with a ^19^F cryogenic probe. The disappearance of ^19^F- and ^1^H-NMR signals of compound **12** in the presence of MabA confirmed the direct binding of this inhibitor to the protein (Figure 2).

Interestingly, a comparison of the ^19^F-NMR signal observed with compound **12** and three previously published MabA inhibitors [20] shows a correlation between the signal decrease and the inhibitory activity of the compounds. Experiments carried out with compound **12**, which displays the best activity, show a total disappearance of the signal in contrast to the other three compounds where the signal is still visible (Supplementary materials Figure S1).

#### 2.2.5. Evaluation of Physico-Chemical Properties and Antimycobacterial Activity

The two reference compounds (**1** and **2**) and three analogs (**12**, **16** and **18**), chemically diverse and of similar potency to the reference compounds, were selected to further evaluate their physico-chemical properties (Table 4). All compounds displayed a solubility greater than 140 µM and a logD between 2.54 and 4.10. Plasma protein binding was measured and these compounds, which are hydrophobic and bear a carboxylic acid function, were highly bound to plasma proteins. The introduction of a pyrazole ring (compound **18**) expectedly decreased lipophilicity but did not reduce plasma protein binding.

**Figure 3:**
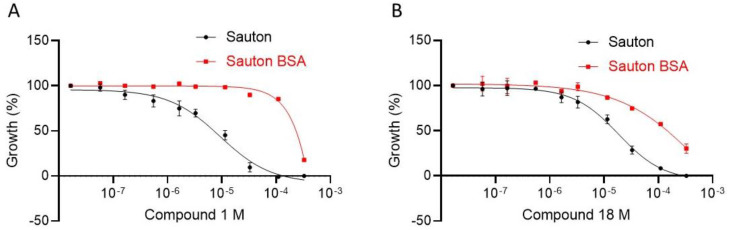
*M. tuberculosis* growth inhibition by compound **1** (**A**) and compound **18** (**B**) in Sauton medium with (red curve) or without (black curve) 12.5 g/L of bovine serum albumin (BSA).

The antibacterial activity of compounds **1** and **2** reported previously was assessed using green fluorescent protein (GFP)-expressing *M. tuberculosis* H37Rv grown in Middlebrook 7H9 liquid medium supplemented with Oleic Albumin Dextrose Catalase (OADC). After 5 days of incubation, the GFP signal was measured and showed that both compounds had an equivalent potency to inhibit 90% of the bacterial growth (MIC_90_ = 300 µM). The bacterial culture medium used in this previous study contained albumin, which we know now may limit the quantity of unbound compounds **1** and **2**. In the present study, the culture medium was therefore switched to Sauton, which does not contain albumin. In these conditions, compound **1** displayed a MIC_90_ of 100 µM while the addition of albumin to the Sauton medium reversed its MIC_90_ to 300 μM (Figure 3A). A similar pattern was observed in the presence of albumin for all compounds tested such as compound **18** (Figure 3B).

The antimycobacterial activity of our five best compounds was then evaluated on GFP-expressing *M. tuberculosis* H37Rv grown in Sauton medium (Table 4). All compounds inhibited bacterial growth with MIC_90_ values between 100 and 300 µM. However, there was no apparent correlation between enzymatic and antibacterial activities.

#### 2.2.6. Analysis of Mycolic Acids Inhibition in *M. tuberculosis*

**Figure 4:**
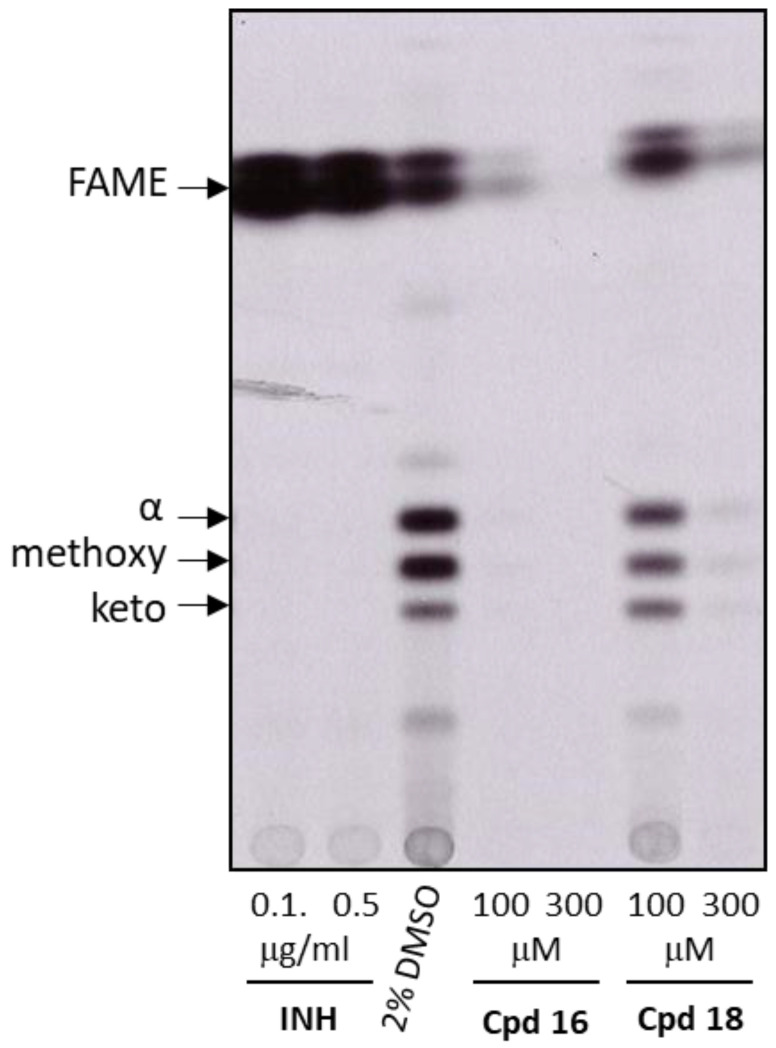
Autoradiogram of TLC analysis of fatty acid methyl esters extracted from the cells *M. tuberculosis* H37Rv cultivated in the presence of INH (positive control), DMSO (negative control), and compounds **16** and **18**. The cells were metabolically labeled with ^14^C-acetate. FAME—fatty acid methyl esters; α-, methoxy-, keto-, refer to the different forms of mycolic acid methyl esters.

To assess whether the studied compounds were able to inhibit the FAS-II system in bacterio, ^14^C metabolic labeling of *M. tuberculosis* H37Rv treated with selected compounds was performed, mycolic acids were isolated, derivatized to corresponding methyl esters and analyzed by TLC (Figure 4). Mycolic acids are synthesized by two fatty acid synthase systems FAS-I and FAS-II. In this assay, *M. tuberculosis* was grown in Sauton medium without BSA, and radiolabeled acetate was used to produce radiolabeled mycolic acids. The fatty acid profiles of the cells treated with 2% DMSO as a control showed the presence of the three types of mycolic acids (alpha, methoxy and keto), as well as standard fatty acid methyl esters (FAME) coming from FAS-I and used as precursors for the mycolic acid’s synthesis. In this assay, a treatment with isoniazid (INH), a FAS-II inhibitor, led to a decrease in mycolic acids synthesis and an accumulation of the FAMEs which are no longer used for the biosynthesis of mycolic acids.

Two MabA inhibitors (compounds **16** and **18**) were selected and tested at 100 and 300 µM in this assay. The solubility of these compounds in Sauton medium was measured and they displayed a solubility greater than 300 µM. TLC analysis showed no specific inhibition of mycolic acids in cells treated with these compounds. It revealed a general dose-dependent decrease in all ^14^C-labeled molecules, correlated to the activity of the compounds measured on *M. tuberculosis* (compound **16** MIC_90_ = 100 µM and compound **18** MIC_90_ = 300 µM). This result indicates that compounds **16** and **18** affect other targets than MabA in mycobacterial cells.

#### 2.2.7. Exploration of the Mechanism of Action of Inhibitors in Bacteria

**Figure 5:**
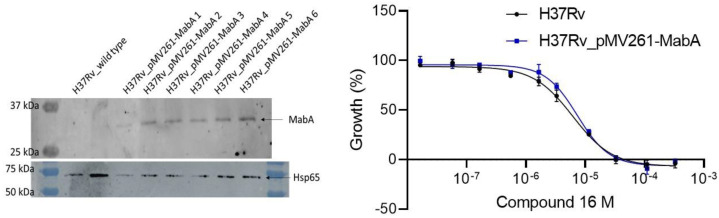
(**Left**) Western blot showing overexpression of MabA in H37Rv_pMV261-MabA, as compared to the parental control. H37Rv_pMV261-MabA 1 through 6 correspond to different clones tested. (**Right**) Antimycobacterial activity of compound **16** on H37Rv (black curve) and H37Rv_pMV261-MabA (blue curve). Curves represent the mean of two independent experiments.

To evaluate the contribution of MabA inhibition in the mechanism of action of the compounds, MabA (Rv1483) was cloned into pMV261 constitutive overexpression plasmid under the control of the strong *hsp60* promoter. The construct was transformed in H37Rv and the overexpression of MabA was verified by Western blot. For an equivalent loading material, as exemplified by the housekeeping protein Hsp65 band, MabA was undetectable in the parental H37Rv, while H37Rv_pMV261-MabA displayed a strong MabA production (Figure 5, left). No difference in activity for the inhibitors was observed between the parental and mabA overexpressing H37Rv, suggesting that the inhibition of MabA plays a minor role, if any, in the mechanism of action of these inhibitors (Figure 5, right).

Several rounds of selection of spontaneous resistance mutants were undertaken at 2× and 3× MIC of compound **16** and this resulted in a bacterial lawn or lack of resistant colonies, respectively. The selection of resistant mutants was also attempted in liquid. Cultures were treated with increasing concentrations of compound **16** in Sauton medium, up to 45 µM, where growth inhibition was apparent by optical density. After 7 days of treatment, the bacteria were pelleted and plated on 7H11 agar plates carrying 2× MIC and 3× MIC of compound **16**. Again, this resulted in the growth of a lawn after 3 months of incubation at 37 °C or in the absence of colonies, respectively.

Transcriptomic profiling can be a useful tool to inform on the mechanism of action of a drug [27]. Therefore, H37Rv was treated with 50 µM of compound **16** in Sauton medium for 2 h after which the bacteria were pelleted, and their RNA extracted for library preparation and RNA sequencing.

**Figure 6:**
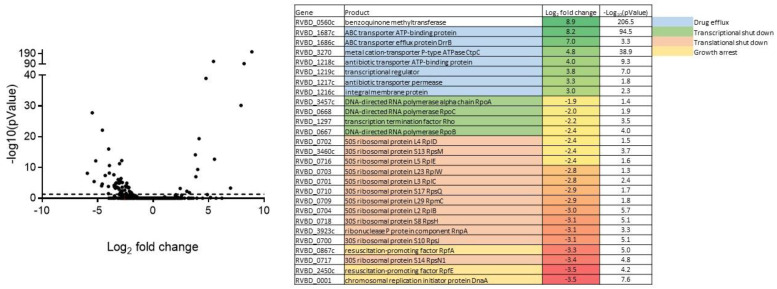
Transcriptomic profile of *M. tuberculosis* response to compound **16** by RNA sequencing.

Transcriptional analysis revealed the downregulation of genes implicated in DNA replication, transcription, translation and growth, in line with the growth-inhibitory effect of the compound (Figure 6). Furthermore, overexpression of drug efflux operons (Rv1687c-1686c, Rv1219c-1216c) was particularly significant suggesting a rapid induction of export of the compound. Lastly, Rv0560c was strongly upregulated. Rv0560c is described as an S-adenosylmethionine-dependent methyltransferase that was shown to be induced by salicylate stress. In fact, our transcriptional results agreed well with described gene expression profile observed after *M. tuberculosis* treatment with salicylate [28].

**Figure 7:**
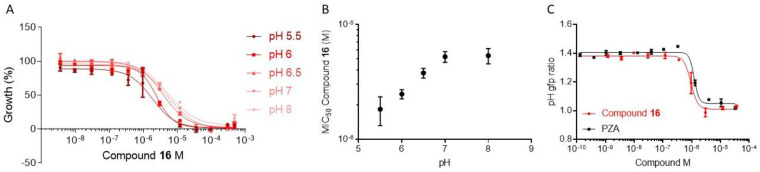
Effect of pH on compound **16** activity. (**A**) Dose response of H37Rv to compound **16** under a range of pH conditions; (**B**) Correlation plot of pH and MIC_50_ of compound **16**; (**C**) Effect of compound **16** and pyrazinamide (PZA) on the maintenance of intrabacterial pH.

The anthranilic acid derivatives are close analogs of salicylic acid and transcriptional profiling reveals that this feature plays a major role in the bacterial response to compound **16** treatment. *M. tuberculosis* is unusually sensitive to weak acids such as salicylic acid. Weak acids display higher potency in decreasing pH conditions, and this was correlated to their ability to act as protonophores, inducing acidification of intrabacterial pH and decreased membrane potential [29]. To determine whether this is a trait of our MabA inhibitors, the activity of compound **16** was tested under a range of pH conditions on H37Rv (Figure 7).

Indeed, decreasing pH improved the potency of compound **16** (Figure 7A,B). Moreover, we used an H37Rv expressing a ratiometric pH-sensitive GFP to measure the effect of compound **16** treatment in the maintenance of the pH homeostasis [30]. Consistently, we observed a dose-dependent decrease in the intrabacterial pH in the presence of compound **16** (Figure 7C). These observations are consistent with our inability to select resistance mutants to compound **16** as the peculiar mechanism of action of these weak acids does not rely on a single target, but rather on a general effect on membrane potential.

Overall, these results suggest that the anthranilic acid core of MabA inhibitors is the main driver of bacterial response, inducing intrabacterial acidification and arrest of replication and fatty acid synthesis [28]. These effects are shared with pyrazinamide (PZA), a prodrug bioactivated by the enzyme PncA into pyrazinoic acid. This compound, which is also poorly active in neutral pH conditions, is nevertheless a cornerstone drug in current TB regimens.

## 3. Materials and Methods

### 3.1. Chemistry

The solvents used for synthesis, analysis and purification were obtained as analytical grade from commercial suppliers and used without further purification. Chemical reagents were obtained from Fisher Scientific, Merck, Fluorochem, Enamine or TCI as reagent grade and used without further purification.

The LC-MS Waters system used included a 2747 sample manager, a 2695 separations module, a 2996 photodiode array detector (200–400 nm), and a Waters Micromass ZQ2000 detector (scan 100–800). The XBridge C18 column (50 mm × 4.6 mm, 3.5 µm, Waters) was used with an injection volume of 20 µL and a mobile phase mixture of water and acetonitrile in gradient-elution. The pH of the mobile phase was adjusted to 3.8 with HCOOH and NH_4_OH to form a buffer solution. Analysis time was 5 min (at a flow rate of 2 mL/min), 10 min (at a flow rate of 1 mL/min) or 30 min (at a flow rate of 1 mL/min). Purity was determined by reversed-phase HPLC with UV detection (215 nm) and all isolated compounds had a purity greater than 95%.

HRMS analysis was conducted on a LC-MS system utilizing a LCT Premier XE mass spectrometer from Waters and a XBridge C18 column (50 mm × 4.6 mm, 3.5 µm, Waters). The mobile phase gradient started with 98% H_2_O 5 mM Ammonium Formate pH 3.8 and reached 100% CH_3_CN 5 mM Ammonium Formate pH 3.8 within 3 min at a flow rate of 1 mL/min.

NMR spectra were recorded on a Bruker DRX-300 spectrometer using the solvent as an internal reference [e.g., 2.50 (residual DMSO-*d*_6_) and 39.52 (DMSO-*d*_6_) ppm for ¹H and ¹³C NMR spectra, respectively]. Chemical shifts (δ) were in parts per million (ppm) and assignments were made using one-dimensional (1D) ^1^H and ^13^C spectra and two-dimensional (2D) HSQC-DEPT, COSY, and HMBC spectra. NMR coupling constants (*J*) were reported in Hertz (Hz) and splitting patterns were indicated as follows: m for multiplet, s for singlet, brs for broad singlet, d for doublet, t for triplet, q for quartet, dd for doublet of doublet, ddd for doublet of doublet of doublet.

Reverse flash chromatography was performed using a CombiFlash^®^ C18 Rf200, which was equipped with C_18_ silica gel cartridges and UV detection (215 and 254 nm) was used to isolate the desired product.

Preparative HPLC was performed using a Varian PRoStar system, which was equipped with an Omni-Sphere 10 µm column C18 Dynamax (250 mm × 41.4 mm) from Agilent Technologies. A gradient starting from 20% MeCN/80% H_2_O/0.1% formic acid and reaching 100% MeCN/0.1% formic acid at a flow rate of 80 mL/min was used with UV detection (215 and 254 nm) to isolate the desired product.

The synthesis process utilizing microwave irradiation was carried out using Biotage^®^ Initiator+.

#### 3.1.1. General Method for the Coupling of Anilines with Isopropoxycarbonyl 3,4-dichlorobenzoate

**Scheme 4:**
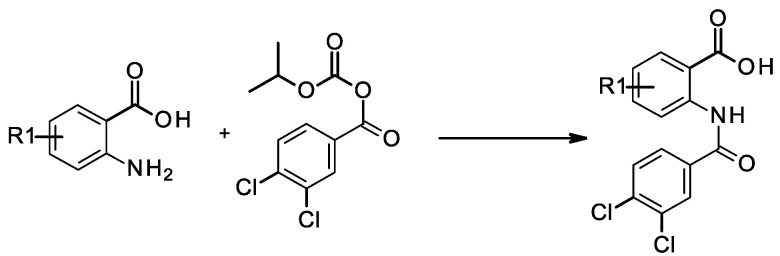
Coupling of anilines with isopropoxycarbonyl 3,4-dichlorobenzoate.

The appropriate amine (1 eq) was dissolved in water (C = 0.2–0.5 M) with sodium carbonate (2 eq). This solution was cooled to 0 °C, and then a solution of isopropoxycarbonyl 3,4-dichlorobenzoate (1.5 eq) in THF (C = 0.33–0.8 M) was added dropwise at 0 °C. The mixture was stirred at RT until completion of the reaction (Scheme 4). The product was solubilized in MeOH, celite was added, solvents were removed under reduced pressure and the product was purified by reverse flash chromatography (MeOH/H_2_O).

##### 5-bromo-2-[(3,4-dichlorobenzoyl)amino]benzoic Acid (**2**)

Prepared following general method Section 3.1.1. Reaction time: 3 h. Yield = 69%. ^1^H-NMR (DMSO-*d*_6_): δ (ppm) 7.50 (dd, *J* = 8.8, *J* = 2.6 Hz, 1H), 7.82 (d, *J* = 8.5 Hz, 1H), 7.96 (dd, *J* = 8.5 Hz, *J* = 2.0 Hz, 1H), 8.13-8.14 (m, 2H), 8.57 (d, *J* = 8.8 Hz, 1H). ^13^C-NMR (DMSO-*d*_6_): δ (ppm) 114.4, 121.0, 127.7, 127.8, 129.6, 131.5, 132.2, 133.2, 134.1, 134.9, 136.1, 140.3, 162.3, 168.5. HRMS (TOF, ES-) *m*/*z* [M-H]^−^: Calculated for C_14_H_7_NO_3_Cl_2_Br 385.8986, found 385.8989.

##### 4-bromo-2-[(3,4-dichlorobenzoyl)amino]benzoic Acid (**14**)

Prepared following general method Section 3.1.1. Reaction time: 2 h. Yield = 23%. ^1^H-NMR (DMSO-*d*_6_): δ (ppm) 12.44 (s, 1H), 8.82 (s, 1H), 8.10 (s, 1H), 7.88–7.97 (m, 3H), 7.42 (d, *J* = 8.0 Hz, 1H) ^13^C-NMR (DMSO-*d*_6_): δ (ppm) 169.9, 163.1, 141.9, 135.7, 135.0, 133.4, 132.4, 131.8, 129.6, 127.8, 127.7, 126.7, 122.8, 117.2. HRMS (TOF, ES-) *m*/*z* [M-H]^−^: Calculated for C_14_H_7_NO_3_BrCl_2_ 389.8986, found 389.8998.

##### 2-[(3,4-dichlorobenzoyl)amino]benzoic Acid (**15**)

Prepared following general method Section 3.1.1. Reaction time: 2 h. Yield = 14%. ^1^H-NMR (DMSO-*d*_6_): δ (ppm) 12.35 (s, 1H), 8.58 (d, *J* = 8.1 Hz, 1H), 8.13 (d, *J* = 1.4 Hz, 1H), 8.05 (dd, *J* = 7.9, 1.2 Hz, 1H), 7.83–7.94 (m, 2H), 7.65 (t, *J* = 7.1 Hz, 1H), 7.23 (t, *J* = 7.5 Hz, 1H). ^13^C-NMR (DMSO-*d*_6_): δ (ppm) 170.4, 162.9, 140.9, 135.5, 135.4, 134.4, 132.3, 131.8, 131.7, 129.6, 127.7, 123.9, 120.6, 118.5. HRMS (TOF, ES-) *m*/*z* [M-H]^−^: Calculated for C_14_H_8_NO_3_Cl_2_ 307.9881, found 307.9886.

##### 2-[(3,4-dichlorobenzoyl)amino]-4-pyrazol-1-yl-benzoic Acid (**18**)

Prepared following general method Section 3.1.1. Reaction time: 2 h. Yield = 36%. ^1^H-NMR (DMSO-*d*_6_): δ (ppm) 9.14 (d, *J* = 1.9 Hz, 1H), 8.43 (d, *J* = 2.2 Hz, 1H), 8.20 (d, *J* = 1.7 Hz, 1H), 8.11 (d, *J* = 8.5 Hz, 1H), 8.01 (dd, *J* = 8.4, 1.8 Hz, 1H), 7.86 (d, *J* = 8.4 Hz, 1H), 7.76 (s, 1H), 7.45 (dd, *J* = 8.4, 2.0 Hz, 1H), 6.55 (s, 1H). ^13^C-NMR (DMSO-*d*_6_): δ (ppm) 169.3, 162.4, 142.1, 141.5, 141.1, 136.3, 134.8, 132.8, 132.2, 131.6, 129.7, 128.2, 127.8, 123.5, 112.0, 109.0, 108.4. HRMS (TOF, ES-) *m*/*z* [M-H]^−^: Calculated for C_17_H_10_N_3_O_3_CL_2_ 374.0099, found 374.0103.

##### 3-[(3,4-dichlorobenzoyl)amino]naphthalene-2-carboxylic Acid (**19**)

Prepared following general method Section 3.1.1. Time of reaction: overnight. Yield = 25%. ^1^H-NMR (DMSO-*d*_6_): δ (ppm) 14.0 (s, 1H), 12.15 (s, 1H), 9.01 (s, 1H), 8.73 (s, 1H), 8.14 (s, 1H), 8.05 (d, *J* = 8.0 Hz, 1H), 7.96-7.84 (m, 3H), 7.64 (t, *J* = 6.8 Hz, 1H), 7.51 (t, *J* = 7.4 Hz, 1H). ^13^C-NMR (DMSO-*d*_6_): δ (ppm) 170.4, 162.8, 136.1, 135.9, 135.5, 135.3, 133.7, 132.3, 131.8, 129.8, 129.6, 129.5, 129.0, 127.7, 127.6, 126.3, 118.4, 117.8. HRMS (TOF,ES-) *m*/*z* [M-H]^−^: Calculated for C_18_H_10_NO_3_Cl_2_ 358.0038, found 358.0046.

#### 3.1.2. Synthesis of 2-[(3,4-dichlorobenzoyl)-methyl-amino]-5-iodo-benzoic Acid (**3**)

To a solution of 2-[(3,4-dichlorobenzoyl)amino]-5-iodo-benzoic acid (1, 0.05 mmol) in a mixture of anhydrous THF and DMF (2 mL, *v*/*v*, 3:1) was added cesium carbonate (0.19 mmol) and iodomethane (0.16 mmol). The mixture was stirred at RT overnight. Lithium hydroxide (0.15 mmol) in water (2 mL) was then added and the reaction was stirred at RT for 5 h. The solvent was removed under reduced pressure and to the white residue was added HCl 1 N (5 mL). The obtained precipitated was filtered to afford the desired product as a white powder. Yield = 88%. ^1^H-NMR (MeOD-*d*_4_): δ (ppm) 8.14 (s, 1H), 7.90 (d, *J* = 7.0 Hz, 1H), 7.44 (s, 1H), 7.33 (d, *J* = 8.0 Hz, 1H), 7.21 (d, *J* = 8.5 Hz, 1H), 7.12 (d, *J* = 7.5 Hz, 1H), 3.38 (s, 3H). ^13^C-NMR (MeOD-*d*_4_): δ (ppm) 168.7, 165.4, 143.5, 142.1, 140.2, 135.8, 133.6, 131.7, 131.6, 130.5, 130.1, 129.7, 127.5, 92.1, 37.0. HRMS (TOF, ES-) *m*/*z* [M-H]^−^: Calculated for C_15_H_9_NO_3_Cl_2_I 447.9004, found 447.9026.

#### 3.1.3. Synthesis of 2-[(3,4-dichlorophenyl)sulfonylamino]-5-iodo-benzoic Acid (**4**)

In a round bottom flask charged with 2-amino-5-iodo-benzoic acid (0.5 mmol) and Na_2_CO_3_ (0.5 mmol) dissolved in water (1.5 mL) was slowly added 3,4-dichlorobenzenesulfonyl chloride (0.5 mmol). During the addition of sulfonyl chloride and the progression of reaction the pH was maintained at a value around 8 (sodium carbonate was added portion-wise if necessary). The resulting mixture was stirred at RT for 30 min. The product precipitated as a yellow solid which was filtered and washed with water, cyclohexane and DCM to afford the desired product as a white powder. Yield = 45%. ^1^H-NMR (DMSO-*d*_6_): δ (ppm) 8.10 (d, *J* = 2.2 Hz, 1H), 7.95 (d, *J* = 2.0 Hz, 1H), 7.79 (d, *J* = 8.4 Hz, 1H), 7.70 (d, *J* = 8.5 Hz, 1H), 7.69 (dd, *J* = 8.6, 4.2 Hz, 1H), 7.19 (d, *J* = 8.7 Hz, 1H), ^13^C-NMR (DMSO-*d*_6_): δ (ppm) 167.7, 142.3, 142.2, 141.4, 139.2, 135.4, 132.0, 131.7, 128.3, 126.6, 121.7, 120.9, 84.7. HRMS (TOF, ES-) *m*/*z* [M-H]^−^: Calculated for C_13_H_7_NO_4_SCl_2_I 469.8518, found 469.8514.

#### 3.1.4. Synthesis of 5-bromo-2-[(3,4-dichlorophenyl)carbamoyl]benzoic Acid (**5**)

5-bromoisobenzofuran-1,3-dione (**7b**, 1.17 mmol), DIEA (2.57 mmol and 3,4-dichloroaniline (1.17 mmol) were introduced in a tube with anhydrous DMF. The reaction mixture was stirred overnight at RT. The solution was evaporated under reduced pressure. The residue was dissolved in acetonitrile and then purified by preparative HPLC (H_2_O/MeCN) to afford one fraction pure and one contaminated. A second purification by preparative HPLC (isocratic, 45/55 H_2_O/MeCN) was attempted and gave a second pure fraction. Pure fractions were pooled. Yield =32%. ^1^H-NMR (DMSO-*d*_6_): δ (ppm) 10.68 (s, NH), 8.05 (d, *J* = 2.2 Hz, 1H), 8.02 (d, *J* = 2.0 Hz, 1H), 7.90 (dd, *J* = 8.1 Hz, *J* = 2.0 Hz, 1H), 7.61 (d, *J* = 8.8 Hz, 1H), 7.57-7.53 (m, 2H). ^13^C-NMR (DMSO-*d*_6_): δ (ppm) 167.2, 166.4, 139.8, 137.6, 135.1, 132.5, 131.4, 131.2, 130.4, 125.4, 123.2, 121.1, 120.0. HRMS (TOF, ES-) *m*/*z* [M-H]^−^: Calculated for C_14_H_7_NO_3_Cl_2_Br 385.8986, found 385.8975.

#### 3.1.5. Synthesis of 5-bromo-2-[(3,4-dichlorobenzenecarbothioyl)amino]benzoic Acid (**6**)

##### Methyl 5-bromo-2-[(3,4-dichlorobenzoyl)amino]benzoate (Intermediate **6a**)

In the round bottom flask, methyl 2-amino-5-bromo-benzoate (2 mmol) and 1 mL of pyridine were added. 3,4-dichlorobenzoyl chloride (2 mmol) dissolved in 2 mL of pyridine was added dropwise to the solution. The mixture was stirred at RT for 1 h. The solvent was removed under reduced pressure, EtOAc was added and a precipitated was formed. It was filtrated to afford 744 mg of white powder leading to a 92% yield.

##### Methyl 5-bromo-2-[(3,4-dichlorobenzenecarbothioyl)amino]benzoate (Intermediate **6b**)

A suspension of Lawesson’s reagent (0.57 mmol), dissolved in anhydrous toluene (2 mL), was added at RT to a stirred solution of methyl 5-bromo-2-[(3,4-dichlorobenzoyl)amino]benzoate (0.285 mmol) in anhydrous toluene (5 mL). The solution was stirred under argon at 110 °C for 4 days. The solvent was removed under reduced pressure, and the crude was purified by flash chromatography (Cyclohexane/EtOAc from 100/0 to 95/5 in 20 min) to afford an impure yellow powder which was used in the next step without further purification.

##### 5-bromo-2-[(3,4-dichlorobenzenecarbothioyl)amino]benzoic Acid (**6**)

In round bottom flask was dissolved methyl 5-bromo-2-[(3,4-dichlorobenzenecarbothioyl) amino]benzoate (0.29 mmol) in 2.5 mL of MeOH. NaOH (1.15 mmol) was dissolved in 2.5 mL of MeOH and 0.1 mL of water and then added to the first solution. The mixture was stirred at reflux. 7 mL of MeOH were added and then the reaction was stirred overnight at reflux. The solvent was then removed under reduced pressure. The crude was dissolved in EtOAc and washed with HCl, brine, dried over MgSO_4_ and evaporated under reduced pressure. The crude was purified by reverse flash chromatography (MeOH/H_2_O from 90/10 to 100/0 in 25 min) to give the desired product as a yellow powder. Yield = 30% (over two steps), ^1^H-NMR (DMSO-*d*_6_): δ (ppm) 9.67 (d, *J* = 8.9 Hz, 1H), 8.17 (d, *J* = 2.6 Hz, 1H), 8.14 (d, *J* = 2.2, 1H), 7.91 (dd, *J* = 8.5 Hz, *J* = 2.2 Hz, 1H), 7.74 (d, *J* = 8.5 Hz, 1H), 7.60 (dd, *J* = 8.9 Hz, *J* = 2.6 Hz, 1H) ^13^C-NMR (DMSO-*d*_6_): δ (ppm) 190.4, 167.8, 144.1, 141.7, 133.8, 133.7, 132.7, 131.4, 130.9, 129.7, 129.3, 127.7, 120.8, 116.9. HRMS (TOF, ES-) *m*/*z* [M-H]^−^: Calculated for C_14_H_7_NO_2_SCl_2_Br 401.8758, found 401.8764.

#### 3.1.6. Synthesis of 5-bromo-2-[3-(3,4-dichlorophenyl)-1,2,4-oxadiazol-5-yl]benzoic Acid (**7**)

##### 3,4-dichloro-*N*′-hydroxy-benzamidine (Intermediate **7a**)

In a 25 mL round bottom flask, 3,4-dichlorobenzonitrile (3 mmol, 516 mg, 1 eq), hydroxyammonium chloride (4.5 mmol, 312 mg, 1.5 eq) and DIEA (6.4 mmol, 1.115 mL, 1.6 eq) dissolved in 4 mL of EtOH were added. The mixture was stirred under reflux at 80 °C for 4 h. The solution was evaporated under reduced pressure. The residue was dissolved in EtOAc (15 mL), washed twice with water (15 mL × 2), once with brine (15 mL), dried over MgSO_4_ filtrated and then evaporated under reduced pressure to afford a white powder. Yield = 76% yield. ^1^H-NMR (DMSO-*d*_6_): δ ppm 9.87 (s, 1H), 7.88 (d, *J* = 1.8 Hz, 1H), 7.67 (dd, *J* = 8.5 Hz, *J* = 1.8 Hz, 1H), 7.62 (d, *J* = 8.5 Hz, 1H), 5.96 (s, 2H).

##### 5-bromoisobenzofuran-1,3-dione (Intermediate **7b**)

In a 25 mL round bottom flask, 4-bromophthalic acid (1050 mg, 4.28 mmol, 1 eq), acetic anhydride (5 mL) and trifluoroacetic anhydride (0.08 mL) were added. The mixture was stirred at 100 °C for 4 h. The mixture was then evaporated under reduced pressure to afford a reddish powder. Yield = 99%. ^1^H-NMR (DMSO-*d*_6_): δ ppm 8.35 (s, 1H), 8.19 (d, *J* = 7.6 Hz, 1H), 8.0 (d, *J* = 7.0 Hz, 1H).

##### 4-bromo-2-methoxycarbonyl-benzoic Acid (Intermediate **7c**)

In a round bottom flask, 5-bromoisobenzofuran-1,3-dione (**7b**, 2.7 mmol) and anhydrous MeOH (5 mL) were added. The solution was stirred at RT for 1 h. Then, the solvent was removed under reduced pressure. The crude was purified with reverse flash chromatography (MeCN/H_2_O 65/35, isocratic) to afford 165 mg of a white powder. Yield = 23%. ^1^H-NMR (CDCl_3_): δ ppm 9.80 (brs, 1H), 7.81–7.84 (m, 2H), 7.72 (dd, *J* = 8.9 Hz, *J* = 2.0 Hz, 1H), 3.94 (s, 3H). ^13^C-NMR (CDCl_3_): δ (ppm) 171.39, 167.26, 135.21, 133.91, 131.76, 131.52, 128.23, 127.31, 53.14.

##### Methyl 5-bromo-2-chlorocarbonyl-benzoate (Intermediate **7d**)

In a round bottom flask charged with 4-bromo-2-methoxycarbonyl-benzoic acid (7c, 0.618 mmol) dissolved in anhydrous DCM (1 mL), 3 drops of anhydrous DMF was added. Oxalyl dichloride (0.741 mmol) was then added dropwise. The solution was stirred at RT for 1 h. The solvent was removed under reduced pressure. The crude was used in the next reaction step without further purification.

##### Methyl 5-bromo-2-[3-(3,4-dichlorophenyl)-1,2,4-oxadiazol-5-yl]benzoate (Intermediate **7e**)

In a round bottom flask, methyl 5-bromo-2-chlorocarbonyl-benzoate (**7d**, 0.616 mmol), pyridine (2 mL) and 3,4-dichloro-*N*′-hydroxy-benzamidine (**7a**, 0.616 mmol) were added. The solution was stirred at 100 ° C for 4h30. The solvent was then removed under reduced pressure. The crude was purified by flash chromatography. Yield = 80%. ^1^H-NMR (DMSO-*d*_6_): δ (ppm) 8.20 (d, *J* = 1.8 Hz, 1H), 8.14 (d, *J* = 2.0 Hz, 1H), 8.07 (dd, *J* = 8.19 Hz, *J* = 2.0 Hz, 1H), 8.05-7.97 (m, 2H), 7.89 (d, *J* =8.3 Hz, 1H), 3.82 (s, 3H).

##### 5-bromo-2-[3-(3,4-dichlorophenyl)-1,2,4-oxadiazol-5-yl]benzoic Acid (**7**)

In a round bottom flask charged with methyl 5-bromo-2-[3-(3,4-dichlorophenyl)-1,2,4-oxadiazol-5-yl]benzoate (**7e**, 0.491 mmol), dissolved in MeOH (5 mL) and water (0.1 mL) was added NaOH (1.9 mmol). The solution was stirred at RT overnight, and then the solution was heated at 65 °C for 1 h. The solvent was removed under reduced pressure. The crude was purified by reverse phase chromatography (MeOH/H_2_O from 10/90 to 100/0 in 40 min) to afford the desired product as a white powder. Yield = 27%. ^1^H-NMR (DMSO-*d*_6_): δ (ppm) 8.21 (d, *J* = 1.7 Hz, 1H), 8.08 (d, *J* = 1.2 Hz,1H), 8.03 (dd, *J* = 1.7 Hz, *J* = 8.4 Hz, 1H), 7.97 (d, *J* = 8.26 Hz, 1H), 7.91–7.82 (m, 2H). ^13^C-NMR (DMSO-*d*_6_): δ (ppm) 176.4, 166.7, 135.0, 134.6, 132.8, 132.7, 132.7, 132.3, 129.1, 127.6, 127.0, 126.6, 122.7. HRMS (TOF, ES-) *m*/*z* [M-H]^−^: Calculated for C_15_H_6_N_2_O_3_Cl_2_Br 410.8939, found 410.8935.

#### 3.1.7. Synthesis of 3,4-dichloro-*N*-[2-(hydroxymethyl)-4-iodo-phenyl]benzamide (**8**)

##### (2,5-dioxopyrrolidin-1-yl)-2-[(3,4-dichlorobenzoyl)amino]-5-iodo-benzoate (Intermediate **8a**)

To a stirred solution of 2-[(3,4-dichlorobenzoyl)amino]-5-iodo-benzoic acid (**1**, 0.34 mmol) dissolved in EtOAc (1 mL) was added *N*-hydroxysuccinimide (0.368 mmol) at 0 °C. A solution of DCC (0.35 mmol) in AcOEt (0.5 mL) was added slowly. 1.5 mL of AcOEt was added. The reaction was allowed to reach RT and stirred overnight. The precipitate of DCU was filtered off and washed with EtOAc. The filtrate was washed successively with saturated aqueous sodium hydrogen carbonate and brine, dried over MgSO_4_, and evaporated under reduced pressure to give the desired product as a white powder. Yield = 64%.

##### 3,4-dichloro-*N*-[2-(hydroxymethyl)-4-iodo-phenyl]benzamide (**8**)

To a stirred solution of (2,5-dioxopyrrolidin-1-yl)-2-[(3,4-dichlorobenzoyl)amino]-5-iodo-benzoate (0.22 mmol) dissolved in THF (0.5 mL) a solution of NaBH_4_ (0.69 mmol) in THF (1.5 mmol) and water (0.2 mL) was added dropwise. The reaction was stirred for 1.5 h at RT. Saturated aqueous ammonium chloride (10 mL) was added to quench the reaction. The aqueous layer was then extracted twice with EtOAc. Then, the organic layer was washed with brine, dried over MgSO_4_, and evaporated under reduced pressure. The crude was purified by reverse phase chromatography (H_2_O/MeOH 90/10-0/100 20 min) to afford the desired product as a white powder. Yield = 58%. ^1^H-NMR (DMSO-*d*_6_): δ (ppm) 10.17 (brs, 1H), 8.15 (d, *J* = 2.0 Hz, 1H), 7.89 (dd, *J* = 8.4 Hz, *J* = 2.0 Hz, 1H), 7.80–7.84 (m, 2H), 7.64 (dd, *J* = 2.1 Hz, *J* = 8.3 Hz, 1H), 7.37 (d, *J* = 8.4 Hz, 1H), 5.57 (brs, 1H), 4.53 (s, 2H). ^13^C-NMR (DMSO-*d*_6_): δ (ppm) 163.4, 139.2, 136.1 (2C), 135.2, 135.1, 135.0, 131.9, 131.4, 129.9, 128.2, 127.5, 91.1, 59.9. HRMS (TOF, ES-) *m*/*z* [M-H]^−^: Calculated for C_14_H_9_NO_2_Cl_2_I 419.9055, found 419.9054.

#### 3.1.8. Synthesis of *N*-(2-carbamoyl-4-iodo-phenyl)-3,4-dichloro-benzamide (**9**)

##### 2-amino-5-iodobenzamide (Intermediate **9a**)

Concentrated sulfuric acid (2 mL) was carefully added dropwise at 0 °C to 2-amino-5-iodobenzonitrile (1 mmol). After completion of the addition, the reaction mixture was allowed to warm from 0 °C to RT and stirred for 72 h. The reaction mixture was poured onto ice and then brought to a pH of 9−10 by addition of concentrated aqueous ammonium hydroxide solution. The resulting solid was collected by filtration, washed three times with water (3 × 1 mL), and three times with a 1:1 mixture of diethyl ether cyclohexane (3 × 1 mL) to afford a pale brown solid which was used in the next step without further purification. Yield = 83%. ^1^H-NMR (DMSO-*d*_6_): δ (ppm) 7.81 (d, *J* = 2.0 Hz, 2H), 7.37 (dd, *J* = 8.7, 2.0 Hz, 1H), 7.14 (bs, 1H), 6.70 (s, 2H, NH2), 6.54 (d, *J* = 8.7 Hz, 1H).

##### *N*-(2-carbamoyl-4-iodo-phenyl)-3,4-dichloro-benzamide (**9**)

Prepared following general method Section 3.1.1. Reaction time: 3 days. The crude was purified by reverse flash chromatography (MeOH/H_2_O from 10/90 to 100/0), to afford the desired product as a white powder. Yield = 9%. ^1^H-NMR (DMSO-*d*_6_): δ (ppm) 12.98 (s, 1H), 8.53 (bs, 1H), 8.42 (d, *J* = 8.8 Hz, 1H), 8.07 (d, *J* = 0.7 Hz, 1H), 8.23 (d, *J* = 1.8 Hz, 1H), 8.01 (bs, 1H), 7.91 (dd, *J* = 8.7, 1.6 Hz, 1H), 7.82–7.89 (m, 2H). ^13^C-NMR (DMSO-*d*_6_): δ (ppm) 169.7, 162.2, 141.0, 139.3, 136.8, 135.0, 134.8, 131.9, 131.3, 129.1, 127.1, 122.3, 121.5, 87.0. HRMS (TOF, ES-) *m*/*z* [M-H]^−^: Calculated for C_14_H_8_N_2_O_2_Cl_2_I 432.9008, found 432.9001.

#### 3.1.9. 3,4-dichloro-*N*-(2-cyano-4-iodo-phenyl)benzamide (**10**)

To a solution of 2-amino-5-iodo-benzonitrile (1 mmol) in pyridine (3 mL), was added portion wise 3,4-dichlorobenzoyl chloride (1.5 mmol) and the reaction mixture was stirred at RT for 1 h. The solvent was removed under reduced pressure, water (5 mL) and EtOAc (5 mL) were added and the mixture was stirred at RT for 15 min. The solid formed was filtered, washed with water, HCl, MeOH, AcOEt and acetonitrile, and then dried in a desiccator. Yield = 64%. ^1^H-NMR (DMSO-*d*_6_): δ (ppm) 10.83 (s, 1H), 8.25 (dd, *J* = 8.6 Hz, 1.8 Hz, 2H), 8.10 (dd, *J* = 8.6, 1.9 Hz, 1H), 7.95 (dd, *J* = 8.4, 1.8 Hz, 1H), 7.87 (d, *J* = 8.4 Hz, 1H), 7.38 (d, *J* = 8.5 Hz, 1H). ^13^C-NMR (DMSO-*d*_6_): δ (ppm) 163.4, 142.6, 140.9, 139.7, 135.1, 133.7, 131.5, 131.1, 129.7, 128.4, 128.1, 115.4, 111.0, 90.7. HRMS (TOF, ES-) *m*/*z* [M-H]^−^: Calculated for C_14_H_6_N_2_OCl_2_I 414.8902, found 414.8906.

#### 3.1.10. 3,4-dichloro-*N*-[4-iodo-2-(1H-tetrazol-5-yl)phenyl]benzamide (**11**)

To a solution of 3,4-dichloro-*N*-(2-cyano-4-iodo-phenyl)benzamide (**10**, 0.24 mmol) dissolved in DMF (0.5 mL), zinc bromide (0.24 mmol) and sodium azide (0.24 mmol) were added. The resulting mixture was heated at 130 °C for 1h30. Supplementary sodium azide (7.8 mg, 0.12 mmol, 0.5 eq) was then added and the mixture was stirred for 30 min. The mixture was cooled to RT and a few drops of NaOH in water (50% *w*/*v*) were added. The resulting emulsion was filtered and HCl 1 N was added to the solution until a pH of around 4 was reached. The formed solid was filtered, washed with water and dried in a desiccator to obtain a pale-yellow powder as the desired product. Yield = 86%. ^1^H-NMR (DMSO-*d*_6_): δ (ppm) 12.09 (s, 1H), 8.42 (d, *J* = 2.0 Hz, 1H), 8.29 (d, *J* = 8.8 Hz, 1H), 8.25 (d, *J* = 2.0 Hz, 1H), 8.02 (dd, *J* = 8.4, 2.1 Hz, 1H), 7.91 (d, *J* = 8.4 Hz, 1H), 7.86 (dd, *J* = 8.7, 2.1 Hz, 1H). ^13^C-NMR (DMSO-*d*_6_): δ (ppm) 162.9, 155.4, 138.8, 136.3, 136.0, 135.0, 134.9, 131.8, 131.2, 129.5, 127.5, 123.8, 118.8, 88.3. HRMS (TOF, ES-) *m*/*z* [M-H]^−^: Calculated for C_14_H_7_N_5_OCl_2_I 457.9072, found 457.9073.

#### 3.1.11. 3,4-dichloro-*N*-[4-iodo-2-(trifluoromethylsulfonylcarbamoyl)phenyl]benzamide (**12**)

A solution of 2-[(3,4-dichlorobenzoyl)amino]-5-iodo-benzoic acid (**1**, 0.25 mmol) and CDI (0.5 mmol) was prepared in dry THF (4 mL) and refluxed under argon for 1 h. Trifluoromethanesulfonamide (0.5 mmol) and DBU (0.75 mmol) were dissolved in anhydrous THF (0.5 mL) and the solution was added to the initial reaction mixture. The reaction was then stirred at RT for 2 h. The solvent was removed under reduced pressure and DCM (4 mL) was added. The organic layer was washed with 5% citric acid aqueous solution and brine, dried over MgSO_4_, and then the solvent was removed under reduced pressure. The crude was purified by flash chromatography (from DCM/MeOH 100/0 to 90/10) to afford the desired product as a white power. Yield = 52%. ^1^H-NMR (DMSO-*d*_6_): δ (ppm) 13.43 (s, 1H), 8.51 (d, *J* = 8.7 Hz, 1H), 8.40 (d, *J* = 1.8 Hz, 1H), 8.10 (d, *J* = 1.5 Hz, 1H), 7.79–7.97 (m, 3H). ^13^C-NMR (DMSO-*d*_6_): δ (ppm) 170.7, 162.3, 140.8, 140.0, 139.5, 135.0, 134.9, 132.0, 131.2, 129.3, 127.0, 124.3, 121.7, 120.1 (q, *J* = 324.0 Hz, CF_3_), 86.3. HRMS (TOF, ES-) *m*/*z* [M-H]^−^: Calculated for C_15_H_7_N_2_O_4_F_3_SCl_2_I 564.8500, found 564.8505.

#### 3.1.12. 3,4-dichloro-*N*-[4-iodo-2-(methylsulfonylcarbamoyl)phenyl]benzamide (**13**)

A solution of 2-[(3,4-dichlorobenzoyl)amino]-5-iodo-benzoic acid (**1**, 0.25 mmol) and CDI (0.5 mmol) was prepared in dry THF (4 mL) and refluxed under argon for 1 h. Methanesulfonamide (0.5 mmol) and DBU (0.75 mmol) were dissolved in anhydrous THF (0.5 mL) and the solution was added to the initial reaction mixture. The reaction was then stirred at RT for 2 h. THF was then removed under reduced pressure and DCM (4 mL) was added. The organic layer was washed with 5% citric acid and brine, dried over MgSO_4_, and then the solvent was evaporated under reduced pressure. The crude was purified by flash chromatography (from DCM/MeOH 100/0 to 90/10). The product precipitated into collected fractions and the solid was filtered and dried in a desiccator to give the desired product as a white powder. Yield = 41%. ^1^H-NMR (DMSO-*d*_6_): δ (ppm) 3.25 (s, 3H), 7.67 (d, *J* = 8.5 Hz, 1H), 7.84 (d, *J* = 8.4 Hz, 1H), 7.93 (ddd, *J* = 8.3, 3.0, 2.1 Hz, 2H), 8.01 (d, *J* = 1.6 Hz, 1H), 8.16 (d, *J* = 1.9 Hz, 1H), 11.28 (bs, 1H). ^13^C-NMR (DMSO-*d*_6_): δ (ppm) 40.9, 88.5, 125.3, 127.4, 127.7, 129.5, 131.1, 131.6, 134.4, 134.9, 136.6, 137.5, 140.8, 162.9, 166.3. HRMS (TOF, ES-) *m*/*z* [M-H]^−^: Calculated for C_15_H_10_N_2_O_4_SCl_2_I 510.8783, found 510.8790.

#### 3.1.13. 5-(2-cyclopropylethynyl)-2-[(3,4-dichlorobenzoyl)amino]benzoic Acid (**16**)

In a round bottom flask charged with 2-[(3,4-dichlorobenzoyl)amino]-5-iodo-benzoic acid (**1**, 0.57 mmol), Pd(PPh_3_)_2_Cl_2_ (0.034 mmol), tetrabutylammonium iodide (0.86 mmol) and copper iodide (0.1 mmol) dissolved in anhydrous acetonitrile (6 mL) was added dropwise TEA (9.7 mmol). The resulting solution was stirred at RT for 10 min, and then ethynylcyclopropane (1.26 mmol) was added dropwise. The reaction mixture was stirred at RT for 1h30, and then the solution was heated at 60 °C for 30 min. The solvent was removed under reduced pressure, the residue was dissolved in EtOAc (20 mL) and the organic layer was washed with an aqueous solution of HCl 1 N, water, brine, dried over MgSO_4_, filtered and the solvent was removed under reduced pressure to afford a brown oil. The crude was purified by reverse flash chromatography to afford a dark beige powder. DCM was added, a precipitate was formed and filtrated to afford the desired product as a white powder. Yield = 33%. ^1^H-NMR (DMSO-*d*_6_): δ (ppm) 12.15 (s, 1H), 8.54 (d, *J* = 8.7 Hz, 1H), 8.10 (s, 1H), 7.94 (s, 1H), 7.85–7.90 (m 2H), 7.63 (d, *J* = 8.7 Hz, 1H), 1.60–1.50 (m, 1H), 0.94–0.82 (m, 2H), 0.79–0.70 (m, 2H). ^13^C-NMR (DMSO-*d*_6_): δ (ppm) 169.8, 162.8, 140.2, 137.1, 135.6, 135.2, 134.4, 132.3, 131.8, 129.6, 127.6, 120.8, 118.5, 94.6, 74.9, 8.9 (2C), 0.2. HRMS (TOF, ES-) *m*/*z* [M-H]^−^: Calculated for C_19_H_12_NO_3_Cl_2_ 372.0194, found 372.0193.

#### 3.1.14. Synthesis of 2-[(3,4-dichlorobenzoyl)amino]-4-ethynyl-benzoic Acid (**17**)

##### 2-amino-4-(2-trimethylsilylethynyl)benzoic Acid (Intermediate **17a**)

In a round bottom flask, 2-amino-4-bromo-benzoic acid (1.15 mmol), triphenyl phosphine (0.11 mmol), copper iodide (0.11 mmol), bis(triphenylphosphine)palladium(II) chloride (0.11 mmol) and anhydrous THF (7.5 mL) were added. The round bottom flask was purged with argon for 30 min, and then TEA (54 mmol) and ethynyl(trimethyl)silane (6.9 mmol) were added dropwise to the solution. The mixture was stirred for 2 h at 80 °C. The resulting mixture was filtered, and the filtrate was evaporated under reduced pressure. The crude was purified by reverse flash chromatography to afford the desired product as a yellow oil. Yield = 32%. ^1^H-NMR (DMSO-*d*_6_): δ (ppm) 7.61-7.49 (m, 6H), 6.80 (d, *J* = 1.5 Hz, 1H), 6.51 (dd, *J* = 1.6 Hz, *J* = 8.1 Hz), 0.21 (s, 9H).

##### 2-[(3,4-dichlorobenzoyl)amino]-4-ethynyl-benzoic Acid (**17**)

Prepared following general method Section 3.1.1. Reaction time: 4 h. The crude was purified by reverse flash chromatography (from MeOH/H_2_O from 10/90 to 100/0). During the purification, the trimetylsilyl was removed by methanol and this led to the desired product as a white powder. Yield = 23%. ^1^H-NMR (DMSO-*d*_6_): δ (ppm) 8.72 (d, *J* = 1.4 Hz, 1H), 8.15 (d, *J* = 1.9 Hz, 1H), 8.04–7.94 (m, 2H), 7.93 (d, *J* = 8.4 Hz, 1H), 7.11 (dd, *J* = 7.9 Hz, *J* = 1.7 Hz, 1H), 4.20 (s, 1H). ^13^C-NMR (DMSO-*d*_6_): δ (ppm) 169.2, 162.4, 141.1, 136.2, 134.9, 132.2, 131.9, 131.6, 129.6, 127.7, 126.1, 125.7, 123.6, 121.8, 84.2, 81.5. HRMS (TOF, ES-) *m*/*z* [M-H]^−^: Calculated for C_16_H_8_NO_3_Cl_2_ 331.9881, found 331.9895.

#### 3.1.15. Synthesis of 2-[(3,4-dichlorobenzoyl)amino]quinoline-3-carboxylic Acid (**20**)

##### Ethyl (*Z*)-2-cyano-3-(2-nitrophenyl)prop-2-enoate (Intermediate **20a**)

In a round bottom flask, 2-nitrobenzaldehyde (604 mg, 4 mmol, 1 eq), ethyl 2-cyanoacetate (0.426 mL, 4 mmol, 1 eq), piperidine (0.435 mL, 4.4 mmol, 1.1 eq) and absolute ethanol (10 mL) were added. The solution was stirred at RT for 1.5 h. A precipitate was formed, filtrated and washed with ethanol to afford the desired product as a white powder. Yield = 58% yield. ^1^H-NMR (DMSO-*d*_6_): δ ppm 8.8 (s, 1H), 8.3 (d, *J* = 8.2 Hz, 1H), 8.0-7.8 (m, 3H), 4.3 (q, *J* = 7.2 Hz, 2H), 1.3 (t, *J* = 7.2 Hz, 3H).

##### Ethyl 2-aminoquinoline-3-carboxylate (Intermediate **20b**)

In a tube, ethyl (*Z*)-2-cyano-3-(2-nitrophenyl)prop-2-enoate (**20a**, 4.45 mmol), Fe (13.4 mmol) and acetic acid (15 mL) were added. The solution was stirred at 80 °C for 1.5 h and then cooled to RT overnight. Water was added until complete dissolution and NaHCO_3_ was added until pH reaches 10. The aqueous layer was extracted twice with EtOAc, and then organic layers were pooled, washed with an aqueous solution of NaHCO_3_, dried over MgSO_4_, filtered and evaporated under reduced pressure to afford the desired product as a yellow powder. Yield = 99%; ^1^H-NMR (DMSO-*d*_6_): δ ppm 8.75 (s, 1H), 7.85 (d, *J* = 7.9 Hz, 1H), 7.62 (t, *J* = 7.9 Hz, 1H), 7.48 (d, *J* = 8.4 Hz, 1H), 7.22 (m, 3H), 4.35 (q, *J* = 7.0 Hz, 2H), 1.36 (t, *J* = 7.0 Hz, 3H).

##### Ethyl 2-[bis(3,4-dichlorobenzoyl)amino]quinoline-3-carboxylate (Intermediate **20c**)

In a tube charged with ethyl 2-aminoquinoline-3-carboxylate (**20b**, 0.7 mmol) dissolved in pyridine (2 mL) was added 3,4-dichlorobenzoyl chloride (1 mmol) dissolved in 1 mL of pyridine. This mixture was heated at 100 °C for 10 min under microwave irradiation. 3,4-dichlorobenzoyl chloride (1 mmol) was then added and the mixture was heated at 100 °C for 10 min under microwave irradiation. Additional 3,4-dichlorobenzoyl chloride (0.5 mmol,) was added and heated at 100 °C under microwave irradiation for 10 min. The solvent was then removed under reduced pressure. The crude was dissolved in EtOAc, washed twice with NaHCO_3_ and the aqueous layer was extracted with EtOAc. Organic layers were pooled, washed with brine (15 mL), dried over MgSO_4_, filtrated and evaporated under reduced pressure. The crude was purified by flash chromatography (from cyclohexane/EtOAc 100/0 to 80/20) to afford an impure crude as a beige powder. EtOH was then added, and the precipitate was filtered and washed with EtOH. The filtrate was evaporated under reduced pressure to give a beige powder as the desire product. Yield = 23%. ^1^H-NMR (DMSO-*d*_6_): δ ppm 9.20 (s, 1H), 8.26 (d, *J* = 7.0 Hz, 1H), 7.99 (t, *J* = 1.3 Hz, 2H), 7.87 (m, 1H) 7.76 (m, 6H), 4.32 (q, *J* = 7.4 Hz, 2H), 1.28 (t, *J* = 7.4 Hz, 3H).

##### 2-[(3,4-dichlorobenzoyl)amino]quinoline-3-carboxylic Acid (**20**)

In a round bottom, ethyl 2-[bis(3,4-dichlorobenzoyl)amino]quinoline-3-carboxylate (**20c**, 0.2 mmol) and sodium hydroxide (0.85 mmol) dissolved in 5 mL of EtOH and 0.1 mL of water was added. The mixture was stirred at RT for 1 h. Water was added, and the precipitate was filtrated to afford the desired product as a white powder. Yield = 20%. ^1^H-NMR (DMSO-*d*_6_): δ (ppm) 8.85 (s, 1H), 8.21 (d, *J* = 1.8 Hz, 1H), 8.0 (dd, *J* = 1.9 Hz, *J* = 8.4 Hz, 1H), 7.94 (d, *J* = 7.8 Hz, 1H), 7.88–7.80 (m, 2H), 7.74–7.64 (m, 1H), 7.50–7.39 (m, 1H). ^13^C-NMR (DMSO-*d*_6_): δ (ppm) 167.9, 161.3, 152.0, 147.4, 140.5, 136.6, 135.0, 132.2, 131.6, 130.7, 129.9, 129.0, 127.9, 127.6, 125.7, 125.2, 121.7. HRMS (TOF, ES-) *m*/*z* [M-H]^−^: Calculated for C_17_H_9_N_2_O_3_Cl_2_ 358.9990, found 358.9989.

### 3.2. Biology

#### 3.2.1. MabA Expression and Purification

*E. coli* BL21 (DE3) bacteria that had been transformed with a specific plasmid, pET28a_MabA-His6, were induced to express the protein of interest (MabA-His6) by adding 0.5 mM IPTG when the optical density reached 0.6. The induced bacteria were grown for 4 h at 37 °C. The bacteria were then harvested by centrifugation and resuspended in a lysis buffer containing Tris (50 mM, pH 7.5), NaCl (300 mM), and imidazole (10 mM), along with DNase and a protease inhibitor (Compete EDTA-free from Roche). The bacteria were lysed by passing them through a French press twice. The MabA-His6 protein was purified using a Ni^2+^-affinity column (His-trap from GE Healthcare), eluted in lysis buffer containing 250 mM imidazole and dialyzed against Tris (50 mM, pH 7.5) and NaCl (300 mM). Glycerol at 10% was added to the protein before it was stored at −80 °C.

#### 3.2.2. MabA Enzymatic Assay

NADPH, acetoacetyl-CoA (AcAcCoA) and butyryl-CoA (BCoA) were obtained from Sigma Aldrich. In this assay, MabA catalyzes the reduction of acetoacetyl-CoA (AcAcCoA) into hydroxybutyryl-CoA (HBCoA) using NADPH as a cofactor [20]. After stopping the enzymatic reaction, HBCoA peak area was measured by LC-MS/MS analysis. Quantification of HBCoA with or without inhibitor was used to calculate a percentage inhibition and then the IC_50_. INH-NADP adduct, which displayed an IC_50_ of 20 µM in this assay, was used as a reference inhibitor [31]. Each compound was tested at least twice.

##### Dose-Response Experiments

We transferred compounds at a concentration of 100 mM in DMSO into low-binding black 384-well plates using an Echo 550 liquid Handler (Labcyte). These compounds were then tested at 8 different concentrations using 3-fold serial dilutions from 1 mM to 0.46 µM, with the DMSO volume being adjusted to keep the concentration across all wells at 1% (200 nL). A reference inhibitor INH-NADP [31,32] was used (100% inhibition at 200 µM) and prepared as described in the literature [32]. We added manually, using a 16-channel VIAFLO II electronic pipette (Integra Biosciences), 3.8 µL of Hepes buffer (100 mM pH 7) and 6 µL of MabA (1.33 µM in Hepes buffer). Compounds were incubated with the MabA enzyme for 30 min at 22 °C. We then added manually 10 µL of a mixture of the substrate AcAcCoA and the cofactor NADPH (100 µM in Hepes buffer) to start the enzymatic reaction. The final concentrations were as follows: MabA at 400 nM, AcAcCoA at 50 µM, NADPH at 50 µM. After 15 min of incubation at 22 °C we stopped the enzymatic reaction by adding manually 10 µL of a solution of trifluoroacetic acid in water at a final concentration of 1%, containing BCoA at a final concentration of 5 µM. BCoA was used as an internal standard for MS/MS analysis. The inhibition percentage of each compound at various concentrations was determined by averaging the results of negative controls (1% DMSO) and positive controls (INH-NADP adduct at 200 µM). The data was then analyzed using GraphPad Prism software and a nonlinear regression method to create dose–response curves. The IC_50_ values are the average of at least two experiments.

$$\%Inh=100-\left(\frac{\left(x-Mean_{C+}\right)\times 100}{Mean_{C-}-Mean_{C+}}\right)$$

##### LC-MS/MS Analysis

AcAcCoA, HBCoA and BCoA were separated using an Acquity UPLC BEH C18 column (2.1 × 50 mm, 1.7 μm, Waters) and detected by multiple-reaction monitoring (MRM) [20]. We used a UPLC Acquity I-class coupled with a Xevo TQD mass spectrometer (Waters). The mobile phases A and B were composed of ammonium acetate 10 mM in water and MeOH, respectively. The mass spectrometer was set to specific parameters (polarity ES+, capillary 1200 V, desolvation temperature 600 °C, cone voltage 46 V, source temperature 150 °C, cone gaz flow 50 L/h, desolvation gaz flow 1200 L/h) and the specific transitions being monitored were AcAcCoA 852.1-342.5 (collision energy: 38 eV), HBCoA 854.1-347.2 (collision energy: 36 eV), BCoA 838.1-331.2 (collision energy: 34 eV). Data was acquired and processed using MassLynx and Target-Lynx software (Waters, France). The amount of HBCoA in each well was determined by dividing the peak area of HBCoA by the peak area of BCoA which was used as an internal standard.

#### 3.2.3. Ligand-Observed NMR Experiments

^19^F NMR measurements were performed at 298 K using 3 mm tubes containing 160 μL of samples on a Bruker 600 MHz Avance III HD spectrometer equipped with a CP-QCI-F cryoprobe specifically designed for ^19^F detection. Both the ^19^F and ^1^H NMR reference spectra were acquired on samples containing compound **12** at a concentration of 100 μM, in an NMR buffer containing 50 mM Tris-Cl pH 7.5, 250 mM NaCl, 1% DMSO-*d*_6_, and 5% D_2_O. Then, ^19^F and ^1^H NMR spectra of the compounds were obtained in the presence of MabA at a concentration of 20 µM in the same NMR buffer. The parameters used for the ^1^H experiments were TD = 16,384 points, NS = 64 scans, relaxation delay D1 = 1 s, carrier frequency O1P = 4.698 ppm, spectral window sw = 16 ppm, and with ^19^F decoupling. The acquisition time was about 2.5 min per sample. The parameters used for the ^19^F experiments were TD = 8192 points, NS = 512 scans, relaxation delay D1 = 2 s, carrier frequency O1P = −65.0 ppm, spectral window sw = 20 ppm, and with ^1^H decoupling. The acquisition time was about 22 min per sample. The NMR data were processed using Bruker Topspin 4.06 software.

#### 3.2.4. MIC Determination

Compounds were dissolved in DMSO at a concentration of 100 mM and transferred to a 384-well low-dead-volume polypropylene plate (LP-0200, labcyte), which was used to prepare assay plates. These compounds were then tested at concentrations ranging from 300 µM to 15 nM. Ten 3-fold serial dilutions of the compounds were made on black Greiner 384-well clear bottom polystyrene plates (Greiner, no. 781091) using an Echo 550 liquid Handler (Labcyte). The DMSO volume was adjusted so that the final concentration of DMSO in all wells was 0.3%. The assay was performed in two independent replicates for each condition.

H37Rv-GFP bacteria were grown to mid log phase in Sauton medium for 7 days. On the day of the experiment, the bacterial cultures were adjusted to a concentration of 2. 10^6^ CFU/mL and 50 µL of the bacterial suspension was added to each assay well. Assay plates were then incubated at 37 °C for 7 days. The fluorescence intensity of the GFP was measured using a Victor Multilabel Plate Reader (PerkinElmer) at Ex/Em = 485/535 nm as a surrogate for growth. The assay also included positive control compounds such as rifampicine (MIC = 4 ng/mL), isoniazid (MIC = 125 ng/mL) and ethionamide (MIC = 2 µg/mL). The growth was normalized to DMSO (100%) and rifampicine (0%) as negative and positive controls, respectively. Data points were then analyzed using GraphPad Prism to calculate MIC values by fitting them to the equation [Y = Bottom + (Top−Bottom)/(1 + ((X^HillSlope)))].

To determine the MIC under different pH conditions, H37Rv was grown to mid log phase in Sauton medium for 7 days in the presence of a final concentration of 100 mM of 2-(*N*-morpholino)ethanesulfonic acid(MES), formerly adjusted at the specified pH (stock solutions included 500 mM MES adjusted at the desired pH and Sauton medium prepared in 80% of its original volume). The MES buffer was kept in the preculture and the assay plates, otherwise following the same above procedure for MIC determination. H37Rv carrying pMV261-MabA [33] was grown in a similar manner as the reference strain.

Curves represent the mean of two independent experiments in simplicate, with the exception of Figure 3A—Sauton BSA—for which only one simplicate was performed.

#### 3.2.5. RNA-seq

H37Rv was grown in Sauton medium until mid-log phase. Bacterial cultures were then diluted to an OD of 0.05 and growth was pursued for 3 days. Treatments with DMSO or compound 18 were initiated on day 3. After 3 h, the bacteria were pelleted by centrifugation at 3200 g for 5 min. The pellet was resuspended using FastRNA-pro Blue Kit (MP biomedicals) in Lysing Matrix B tubes (MP biomedicals). Bacterial suspensions were bead beaten in a FastPrep FP120 cell disruptor (MP Biomedicals) at 6.5 m/s for 40 s. Total RNA extraction was performed on the resulting lysate, following the manufacturer’s instructions. QIAseq FastSelect 5S/16S/23S Kit (335925, Qiagen, Les Ulis, France) was used for ribosomal RNA depletion according to manufacturer’s instructions. TrueSeq Stranded mRNA LT kit (15032611-14, Illumina, Evry, France) and QIAseq Stranded Total RNA Lib Kit (180743, Qiagen, Les Ulis, France) were used for sequencing library preparation of biological duplicates, respectively, according to manufacturer’s instructions. Illumina NextSeq 500 system (Illumina, Evry, France) was used in a high-output mode according to the manufacturer’s protocols for the sequencing of cDNA libraries. All samples were multiplexed on one lane of the flow cell. Single-read mode collected reads of lengths of 150 bp. Illumina quality control tools using default settings were used for raw RNA-seq reads processing. Mapping all the reads on *M. tuberculosis* rRNA sequences using Bowtie2 (http://bowtie-bio.sourceforge.net/bowtie2/index.shtml (accessed on 15 December 2021)) allowed filtering out of residual rRNA-specific reads. RNA sequencing data analysis was performed on the Rockhopper open-source software package using default parameters (http://cs.wellesley.edu/~btjaden/Rockhopper/ (accessed on 15 December 2021)). Transcript abundance levels were quantified using the Reads Per Kilobase per Million mapped reads (RPKM).

#### 3.2.6. Measurement of Intrabacterial pH

Plates were prepared as described previously for the MIC determination (3.2.4). H37Rv carrying pUV15-pHGFP (Addgene 70045) were grown until the mid-log phase in 7H9—tween (0.05%)—glycerol (0.2%)—OADC. The bacteria were harvested by centrifugation, washed twice in PBS, resuspended in sodium phosphate citrate buffer pH 4.5 at OD 0.4 and incubated at 37 °C. GFP fluorescence was read on day 0 and day 2 of incubation at ex/em 405/535 nm and 485/535 nm. Intrabacterial pH was extracted with the formula: (Flu_405/535_/Flu_485/535_).

#### 3.2.7. Western Blot

H37Rv and H37Rv_pMV-MabA were grown in Sauton to OD 0.5. A total of 1 mL of bacterial culture was pelleted. Whole-cell lysate was loaded on reducing SDS-PAGE. Proteins were subsequently transferred to nitrocellulose membrane, blocked and probed with mouse anti-MabA or anti-Hsp65 antibodies (Eurogentec, Liège, Belgium) and goat anti-mouse HRP (ab6789, Abcam, Cambridge, UK), and finally revealed by chemiluniescence (RPN2232, Amersham, Buckinghamshire, UK).

#### 3.2.8. Metabolic Labelling with ^14^C Acetate and Mycolic Acids Analysis

*M. tuberculosis* H37Rv cells were cultured in Sauton media with 0.025% (*v*/*v*) Tyloxapol shaking (120 rpm) at 37 °C until O.D._600nm_ reached 0.48. The studied compounds were dissolved in DMSO and added in final concentrations 0, 100 and 300 μM. The final concentration of DMSO was kept at 2%. After 24 h cultivation (statically at 37 °C) ^14^C acetate [ARC; specific activity 106 mCi/mmol] was added to each of the cultures at a final concentration of 0.5 µCi/mL. After the next 24 h of cultivation, the cells were harvested.

Methyl esters of fatty acids (FAME) and mycolic acids (MAME) were prepared as previously described [34]. Dried extracts were dissolved in 50 μL chloroform: methanol (2:1), 5 μL were loaded on TLC plates and different forms of methyl esters were separated in *n*-hexane/ethyl acetate [95:5], 3 runs and visualized by autoradiography.

### 3.3. Physico-Chemical Properties

These experiments were analyzed using a UPLC Acquity I-class coupled with a Xevo TQD mass spectrometer (Waters) under MRM detection. Source parameters were set as follows: capillary 0.5 kV, desolvation temperature 600 °C, source temperature 150 °C, cone gaz flow 50 L/h, desolvation gaz flow 1200 L/h. Transitions monitored were as follows: compound **1**, 433.8-389.9 (polarity ES-, cone voltage 42 V, collision energy 18 eV); compound **2**, 385.9-342.0 (polarity ES-, cone voltage 40 V, collision energy 16 eV); compound **12**, 564.9-147.9 (polarity ES-, cone voltage 46 V, collision energy 28 eV); compound **16**, 372.0-328.0 (polarity ES-, cone voltage 44 V, collision energy 18 eV); compound **18**, 376.0-186.0 (polarity ES+, cone voltage 30 V, collision energy 16 eV).

#### 3.3.1. LogD

A total of 5 μL of a 10 mM solution of the compound in DMSO was diluted in 245 µL of a 1/1 octanol/PBS mixture at pH 7.4. The mixture was gently shaken for 2 h at room temperature. A total of 10 µL of each phase were diluted in 490 μL of MeOH and analyzed by LC-MS/MS. Each compound was tested in triplicate. Log D was determined as the logarithm of the ratio of the concentration of product in octanol and PBS, determined by mass signals.

#### 3.3.2. Solubility

A total of 5 µL of 10 mM of compound solution in DMSO was diluted either in 245 μL of PBS pH 7.4 (triplicate) or in 245 µL of MeOH (triplicate twice). After gently stirring 24 h at room temperature, the solutions were centrifuged for 5 min at 4000 rpm and filtered over 0.45 μm filters (Millex-LH Millipore), except one of the MeOH triplicates. Then, 10 µL of each solution was diluted in 490 µL of MeOH and analyzed by LC-MS/MS. The solubility is determined according to the following formula:

Solubility (μM) = [AUC_(filtered PBS)_/AUC_(not filtered MeOH)_] × 200

The test was validated if:

[AUC_(not filtered MeOH)_ − AUC_(filtered MeOH)_]/AUC_(not filtered MeOH)_ ≤ 10%

#### 3.3.3. Plasma Protein Binding

As it was anticipated that the compounds were likely to be highly bound, a 10% plasma (plasma beforehand diluted in a 1 to 10 ratio in a phosphate buffer pH 7.2) was used for this experiment. The 10% plasma, spiked with the tested compound at 10 µM final (0.1% DMSO), was added to the red-ring chamber of a RED (Rapid Equilibrium Dialysis) device. Blank phosphate buffer pH 7.2 was added to the outer chamber of the RED device and the plate was placed at 37 °C with shaking. After 4 h of incubation, aliquots of the buffer and plasma chambers were removed and the concentration in each compartment was determined by LC-MS/MS analysis.

The calculation of the unbound fraction (fu) is performed according to the formula:

f_u 10%_ = 1 − [(**A**_Plasma,4h_ − **A**_PBS,4h_)/**A**_Plasma,4h_]

The following equation is used to convert from the fraction unbound at 10% to a fraction unbound at 100%: f_u 100%_ = f_u 10%_/(10 − 9f_u 10%_)

The percentage of recovery was calculated according to:

% Recovery = 100 × [(**V**_PBS_ × **A**_PBS,4h_) + (**V**_plasma_ × **A**_plasma,4h_)]/(**V**_plasma_ × **A**_plasma,0h_)

where A is the area corresponding to the compound and V, the volume of solution presents in each compartment (i.e., V_PBS_ = 350 μL and V_Plasma_ = 200 μL).

## 4. Conclusions

Our work led to the discovery and optimization of the first MabA inhibitors. Twenty compounds sharing an anthranilic acid scaffold were synthesized and tested as MabA inhibitors to delineate structure–activity relationships. These SARs showed that the replacement of the amide function with a sulfonamide or a thioamide function was tolerated and that an acidic function was necessary for the activity. Ligand-observed NMR experiments confirmed the direct binding of a fluorinated anthranilic acid to MabA. The antimycobacterial activity of 5 MabA inhibitors, selected based on their potency and chemical diversity, was evaluated on *M. tuberculosis* H37Rv grown in Sauton medium and although all the compounds were able to inhibit bacterial growth, there was no apparent correlation between enzymatic and antibacterial activities.

Metabolic labeling of *M. tuberculosis* H37Rv grown in the presence of two studied compounds did not reveal any changes in mycolic acids profiles suggesting that these inhibitors do not affect the FAS-II system. Further investigation of the mechanism of action of these compounds was carried out by overexpressing MabA, attempting to select resistance mutants and using transcriptional analysis. These experiments led us to conclude that, although anthranilic acid derivatives inhibit MabA in vitro, the activity observed in bacteria was partly due to the presence of the carboxylic acid moiety, which induces internal pH changes. These effects are shared with pyrazinamide, which historically allowed to reduce TB treatment duration from 9 to 6 months. This major outcome is attributed to the ability of pyrazinamide to target intracellular bacilli in acidified phagosomes. Therefore, compound **16** or its derivatives could be tested in combination with anti-TB drugs to assess whether a synergistic effect is observed as with pyrazinamide.

## Figures and Tables

**Table 1:** Inhibition of MabA enzymatic activity by compounds **1**–**7**.

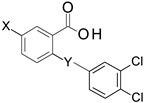			
Cpd	X	-Y-	IC_50_ (µM)
**1**	I	-NH-CO-	38 ± 6
**3**	I	-N(CH_3_)-CO-	>200
**4**	I	-NH-SO_2_-	40
**2**	Br	-NH-CO-	45
**5**	Br	-CO-NH-	268
**6**	Br	-NH-CS-	39
**7**	Br	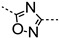	102

**Table 2:** Inhibition of MabA enzymatic activity by compounds **1**, **8**-**13**.

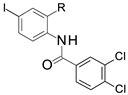		
Cpd	R	IC_50_ (µM)
**1**	-COOH	38 ± 6
**8**	-CH_2_-OH	>333
**9**	-CONH_2_	>500
**10**	-C≡N	>1000
**11**	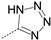	24
**12**	-CO-NH-SO_2_-CF_3_	35
**13**	-CO-NH-SO_2_-CH_3_	28

**Table 3:** Biological activities of compounds **1**, **2**, **14**-**20**.

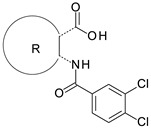		
Cpd	R	IC_50_ (µM)
**1**	* 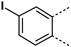 *	38
**2**	* 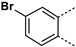 *	45
**14**	* 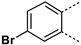 *	34
**15**	* 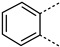 *	>1000
**16**	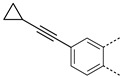	60
**17**	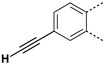	77
**18**	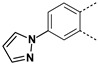	23
**19**	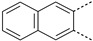	33
**20**	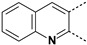	76

**Table 4:** Biological activities and physico-chemical properties of compounds **1**, **2**, **12**, **16**, **18**.

Cpd	Structure	IC_50_ (µM)	MIC_90_ (µM)	Solubility (µM)	LogD_7_._4_	PPB (%)
**1**	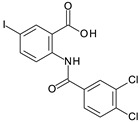	38	100	>200	3.401	99.86
**2**	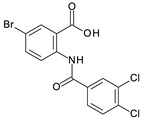	45	100	181	2.998	-
**12**	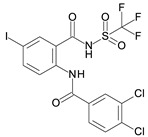	35	300	180	4.102	99.98
**16**	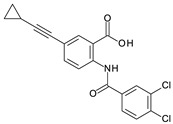	60	100	>200	-	99.94
**18**	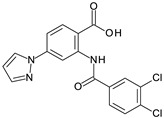	23	300	149	2.537	>99.99

## Data Availability

Data is contained within the article and supplementary material.

## Supplementary Materials

The following supporting information can be downloaded at: https://www.mdpi.com/article/10.3390/ph16030335/s1, Figure S1: 1D ^19^F NMR spectra of 4 compounds alone and in the presence of MabA; Figure S2: Western blot showing overexpression of MabA in H37Rv_pMV261-MabA, as compared to the parental control; Figures S3–S40: ^1^H NMR, ^13^C NMR and HRMS data of compounds **2**–**20**.

## Abbreviations

AcAcCoA, acetoacetyl-CoA; anh, anhydrous; BCoa, butyryl-CoA; BSA, bovine serum albumin; CDI, carbonyldiimidazole; FAME, fatty acid methyl ester; FAS, fatty-acid synthase; DBU, 1,8-Diazabicyclo [5.4.0]undec-7-ene; DCC, dicyclohexylcarbodiimide; DCM, dichloromethane; DMF, dimethylformamide; DMSO, dimethylsulfoxide; EtOAc, ethyl acetate; eq, equivalent; GFP, green fluorescent protein; HBCoA, hydroxybutyryl-CoA; HIV, human immunodeficiency virus; INH, isoniazid; MeCN, acetonitrile; MDR, multidrug-resistant; MeOH, methanol; MIC, minimum inhibitory concentration; NADPH, nicotinamide adenine dinucleotide phosphate; OADC, Oleic Albumin Dextrose Catalase; PZA, pyrazinamide; RT, room temperature; SAR, structure-activity relationships; TB, tuberculosis; TBAF, tetra-*n*-butylammonium fluoride; TBAI, tetra-*n*-butylammonium iodide; TEA, triethylamine; THF, tetrahydrofuran; TLC, thin-layer chromatography; WHO, World Health Organization; XDR, extensively drug-resistant.

## References

[1] MacNeil A., Glaziou P., Sismanidis C., Date A., Maloney S., Floyd K. (2020). Global Epidemiology of Tuberculosis and Progress Toward Meeting Global Targets—Worldwide, 2018. Morb. Mortal. Wkly. Rep..

[2] World Health Organization (2022). Global Tuberculosis Report.

[3] Cox E., Laessig K. (2014). FDA Approval of Bedaquiline—The Benefit–Risk Balance for Drug-Resistant Tuberculosis. N. Engl. J. Med..

[4] Ryan N.J., Lo J.H. (2014). Delamanid: First Global Approval. Drugs.

[5] Keam S.J. (2019). Pretomanid: First Approval. Drugs.

[6] Torres N.M.C., Rodríguez J.J.Q., Andrade P.S.P., Arriaga M.B., Netto E.M. (2019). Factors Predictive of the Success of Tuberculosis Treatment: A Systematic Review with Meta-Analysis. PLoS ONE.

[7] Dulberger C.L., Rubin E.J., Boutte C.C. (2020). The Mycobacterial Cell Envelope—A Moving Target. Nat. Rev. Microbiol..

[8] Abrahams K.A., Besra G.S. (2021). Synthesis and Recycling of the Mycobacterial Cell Envelope. Curr. Opin. Microbiol..

[9] Marrakchi H., Lanéelle M.-A., Daffé M. (2014). Mycolic Acids: Structures, Biosynthesis, and Beyond. Chem. Biol..

[10] Batt S.M., Minnikin D.E., Besra G.S. (2020). The Thick Waxy Coat of Mycobacteria, a Protective Layer against Antibiotics and the Host’s Immune System. Biochem. J..

[11] Bhatt A., Molle V., Besra G.S., Jacobs W.R., Kremer L. (2007). The Mycobacterium Tuberculosis FAS-II Condensing Enzymes: Their Role in Mycolic Acid Biosynthesis, Acid-Fastness, Pathogenesis and in Future Drug Development. Mol. Microbiol..

[12] Parish T., Roberts G., Laval F., Schaeffer M., Daffé M., Duncan K. (2007). Functional Complementation of the Essential Gene FabG1 of Mycobacterium Tuberculosis by Mycobacterium Smegmatis FabG but Not Escherichia Coli FabG. J. Bacteriol..

[13] Brown A.K., Bhatt A., Singh A., Saparia E., Evans A.F., Besra G.S. (2007). Identification of the Dehydratase Component of the Mycobacterial Mycolic Acid-Synthesizing Fatty Acid Synthase-II Complex. Microbiology.

[14] Vilchèze C., Morbidoni H.R., Weisbrod T.R., Iwamoto H., Kuo M., Sacchettini J.C., Jacobs W.R. (2000). Inactivation of the InhA-Encoded Fatty Acid Synthase II (FASII) Enoyl-Acyl Carrier Protein Reductase Induces Accumulation of the FASI End Products and Cell Lysis of Mycobacterium Smegmatis. J. Bacteriol..

[15] Bhatt A., Kremer L., Dai A.Z., Sacchettini J.C., Jacobs W.R. (2005). Conditional Depletion of KasA, a Key Enzyme of Mycolic Acid Biosynthesis, Leads to Mycobacterial Cell Lysis. J. Bacteriol..

[16] North E.J., Jackson M., Lee R.E. (2014). New Approaches to Target the Mycolic Acid Biosynthesis Pathway for the Development of Tuberculosis Therapeutics. Curr. Pharm. Des..

[17] Banerjee A., Dubnau E., Quemard A., Balasubramanian V., Um K.S., Wilson T., Collins D., de Lisle G., Jacobs W.R. (1994). InhA, a Gene Encoding a Target for Isoniazid and Ethionamide in Mycobacterium Tuberculosis. Science.

[18] Grzegorzewicz A.E., Korduláková J., Jones V., Born S.E.M., Belardinelli J.M., Vaquié A., Gundi V.A.K.B., Madacki J., Slama N., Laval F. (2012). A Common Mechanism of Inhibition of the Mycobacterium Tuberculosis Mycolic Acid Biosynthetic Pathway by Isoxyl and Thiacetazone. J. Biol. Chem..

[19] Kremer L., Douglas J.D., Baulard A.R., Morehouse C., Guy M.R., Alland D., Dover L.G., Lakey J.H., Jacobs W.R., Brennan P.J. (2000). Thiolactomycin and Related Analogues as Novel Anti-Mycobacterial Agents Targeting KasA and KasB Condensing Enzymes in Mycobacterium Tuberculosis. J. Biol. Chem..

[20] Faïon L., Djaout K., Frita R., Pintiala C., Cantrelle F.X., Moune M., Vandeputte A., Bourbiaux K., Piveteau C., Herledan A. (2020). Discovery of the First Mycobacterium Tuberculosis MabA (FabG1) Inhibitors through a Fragment-Based Screening. Eur. J. Med. Chem..

[21] Marrakchi H., Ducasse S., Labesse G., Montrozier H., Margeat E., Emorine L., Charpentier X., Daffé M., Quémard A. (2002). MabA (FabG1), a Mycobacterium Tuberculosis Protein Involved in the Long-Chain Fatty Acid Elongation System FAS-II. Microbiology.

[22] Reed C.W., Washecheck J.P., Quitlag M.C., Jenkins M.T., Rodriguez A.L., Engers D.W., Blobaum A.L., Jeffrey Conn P., Niswender C.M., Lindsley C.W. (2019). Surveying Heterocycles as Amide Bioisosteres within a Series of MGlu 7 NAMs: Discovery of VU6019278. Bioorg. Med. Chem. Lett..

[23] Claffey M.M., Helal C.J., Verhoest P.R., Kang Z., Fors K.S., Jung S., Zhong J., Bundesmann M.W., Hou X., Lui S. (2012). Application of Structure-Based Drug Design and Parallel Chemistry to Identify Selective, Brain Penetrant, in Vivo Active Phosphodiesterase 9A Inhibitors. J. Med. Chem..

[24] Wilcken R., Zimmermann M.O., Bauer M.R., Rutherford T.J., Fersht A.R., Joerger A.C., Boeckler F.M. (2015). Experimental and Theoretical Evaluation of the Ethynyl Moiety as a Halogen Bioisostere. ACS Chem. Biol..

[25] Buchholz C.R., Pomerantz W.C.K. (2021). 19 F NMR Viewed through Two Different Lenses: Ligand-Observed and Protein-Observed 19 F NMR Applications for Fragment-Based Drug Discovery. RSC Chem. Biol..

[26] Mureddu L.G., Vuister G.W. (2022). Fragment-Based Drug Discovery by NMR. Where Are the Successes and Where Can It Be Improved?. Front. Mol. Biosci..

[27] Freiberg C., Brötz-Oesterhelt H., Labischinski H. (2004). The Impact of Transcriptome and Proteome Analyses on Antibiotic Drug Discovery. Curr. Opin. Microbiol..

[28] Denkin S., Byrne S., Jie C., Zhang Y. (2005). Gene Expression Profiling Analysis of Mycobacterium Tuberculosis Genes in Response to Salicylate. Arch. Microbiol..

[29] Zhang Y., Zhang H., Sun Z. (2003). Susceptibility of Mycobacterium Tuberculosis to Weak Acids. J. Antimicrob. Chemother..

[30] Vandal O.H., Pierini L.M., Schnappinger D., Nathan C.F., Ehrt S. (2008). A Membrane Protein Preserves Intrabacterial PH in Intraphagosomal Mycobacterium Tuberculosis. Nat. Med..

[31] Ducasse-Cabanot S., Cohen-Gonsaud M., Marrakchi H., Nguyen M., Zerbib D., Bernadou J., Daffé M., Labesse G., Quémard A. (2004). In Vitro Inhibition of the Mycobacterium Tuberculosis β-Ketoacyl-Acyl Carrier Protein Reductase MabA by Isoniazid. Antimicrob. Agents Chemother..

[32] Nguyen M., Claparols C., Bernadou J., Meunier B. (2001). A Fast and Efficient Metal-Mediated Oxidation of Isoniazid and Identification of Isoniazid-NAD(H) Adducts. ChemBioChem.

[33] Veyron-Churlet R., Guerrini O., Mourey L., Daffé M., Zerbib D. (2004). Protein-Protein Interactions within the Fatty Acid Synthase-II System of Mycobacterium Tuberculosis Are Essential for Mycobacterial Viability. Mol. Microbiol..

[34] Phetsuksiri B., Baulard A.R., Cooper A.M., Minnikin D.E., Douglas J.D., Besra G.S., Brennan P.J. (1999). Antimycobacterial Activities of Isoxyl and New Derivatives through the Inhibition of Mycolic Acid Synthesis. Antimicrob. Agents Chemother..

