# Supplementary material for: Exploring the Antitubercular Activity of Anthranilic Acid Derivatives: From MabA (FabG1) Inhibition to Intrabacterial Acidification
Source: Pharmaceuticals (Basel). 2023 Feb 22;16(3):335. doi: 10.3390/ph16030335 (PMC10057394; doi:10.3390/ph16030335)
Supplement: Supplementary file 1 [file pharmaceuticals-16-00335-s001.zip › pharmaceuticals-2208054-supplementary.pdf]

Supplementary materials

Exploring the antitubercular activity of anthranilic acid derivatives: from MabA (FabG1) inhibition to intrabacterial acidification

Léo Faïon [1,†], Kamel Djaout [2,†], Catalin Pintiala [1], Catherine Piveteau [1], Florence Leroux [1], Alexandre Biela [1], Stéphanie Slupek [2], Rudy Antoine [2], Monika Záhorszká [3], Francois-Xavier Cantrelle [4,5], Xavier Hanoulle [4,5], Jana Korduláková [3], Benoit Deprez [1], Nicolas Willand [1], Alain R. Baulard [2,*], Marion Flipo [1,*]

[1] Univ. Lille, Inserm, Institut Pasteur de Lille, U1177 - Drugs and Molecules for living Systems, F-59000 Lille, France
[2] Univ. Lille, CNRS, Inserm, CHU Lille, Institut Pasteur de Lille, U1019 - UMR 9017 - CIIL - Center for Infection and Immunity of Lille, F-59000 Lille, France
[3] Department of Biochemistry, Faculty of Natural Sciences, Comenius University in Bratislava, Mlynská Dolina, Ilkovičova 6, 842 15 Bratislava, Slovakia
[4] Univ. Lille, Inserm, CHU Lille, Institut Pasteur de Lille, U1167 - RID-AGE - Risk Factors and Molecular Determinants of Aging-Related Diseases, F-59000 Lille, France
[5] CNRS, ERL9002 - Integrative Structural Biology, F-59000 Lille, France
* Correspondence: alain.baulard@inserm.fr (A.R.B.); marion.flipo@univ-lille.fr (M.F.)
† These authors contributed equally to this work.

Figure S1: 1D ^{19}F NMR spectra of 4 compounds alone and in the presence of MabA

Figure S2: Western blot showing overexpression of MabA in H37Rv_pMV261-MabA, as compared to the parental control.

Figures S3-S40: ^{1}H NMR, ^{13}C NMR and HRMS data of compounds **2-20**

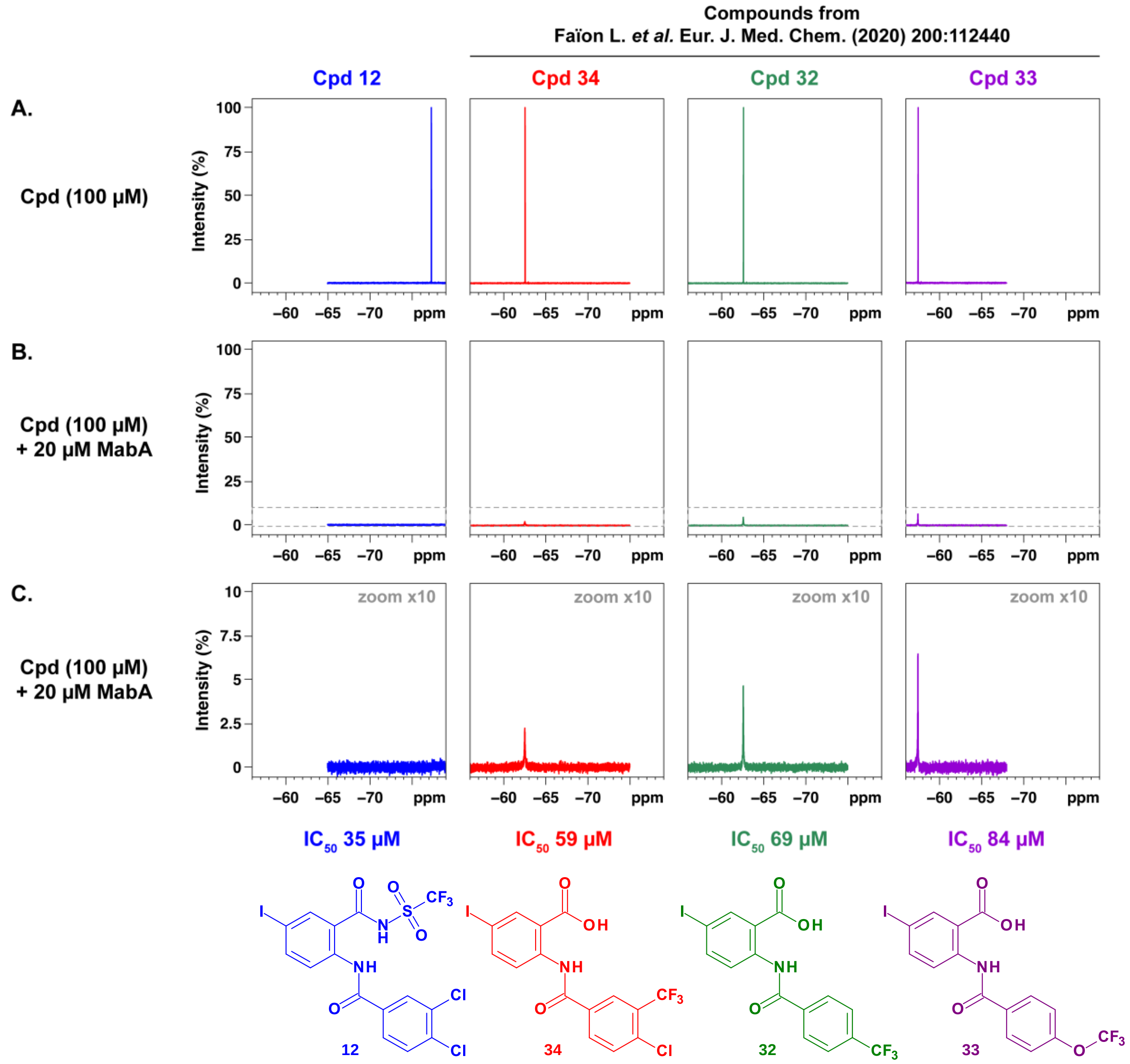

Figure S1: 1D ^{19}F NMR spectra of compound **12** (this study) and compounds **34**, **32** and **33** from Faion L. *et al.* [20] (in blue, red, green and violet, respectively) at 100 µM alone (**A.**) and in the presence of MabA at 20 µM (**B.** and **C.**). The spectra in C. correspond to the ones in B. with a 10x magnification of the y-axis. The IC_{50} for each compound is indicated at the bottom.

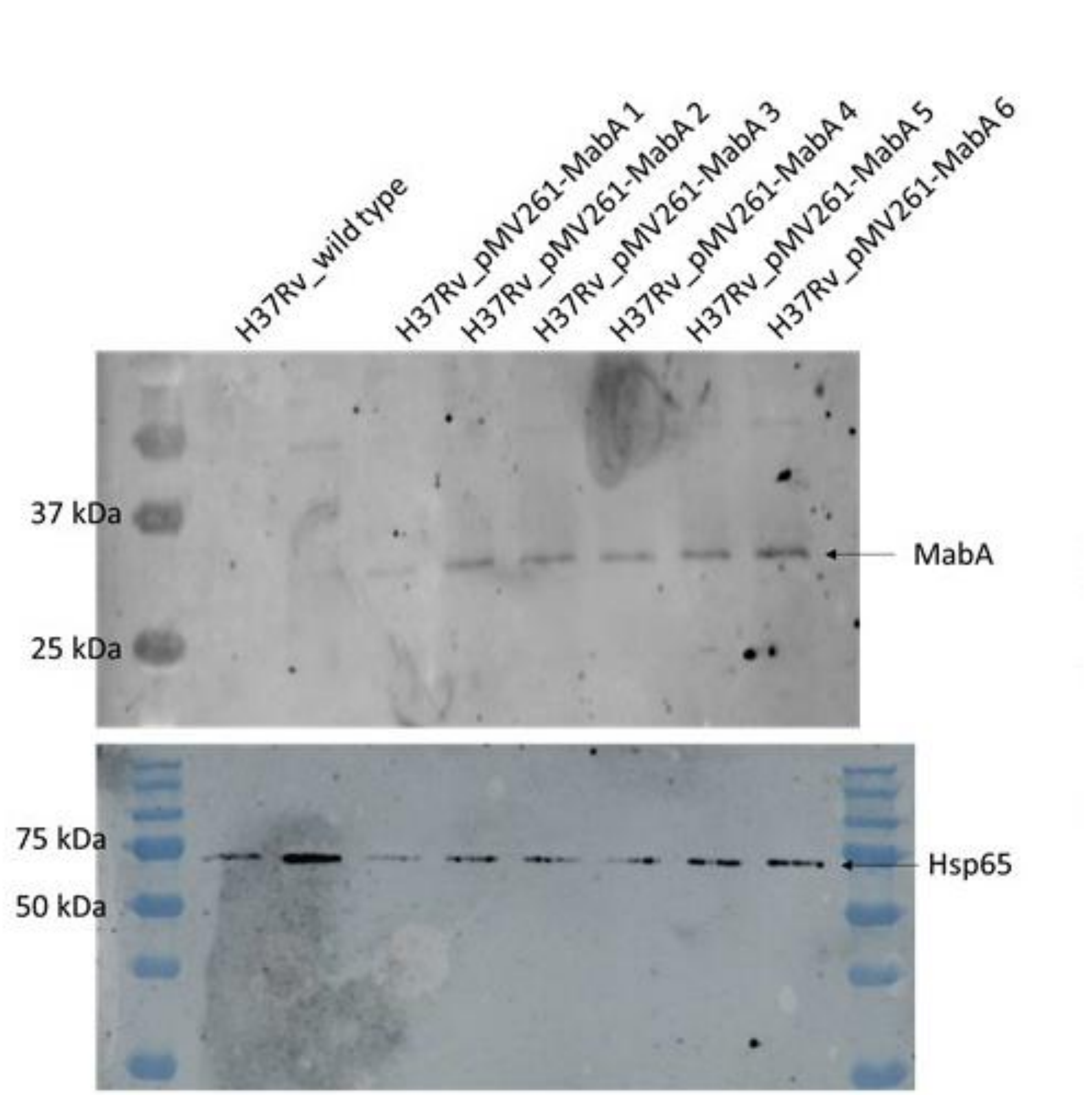

Figure S2: Western blot showing overexpression of MabA in H37Rv_pMV261-MabA, as compared to the parental control. H37Rv_pMV261-MabA 1 through 6 correspond to different clones tested.

^{1}H NMR of compound 2 (300 MHz, DMSO-d6)

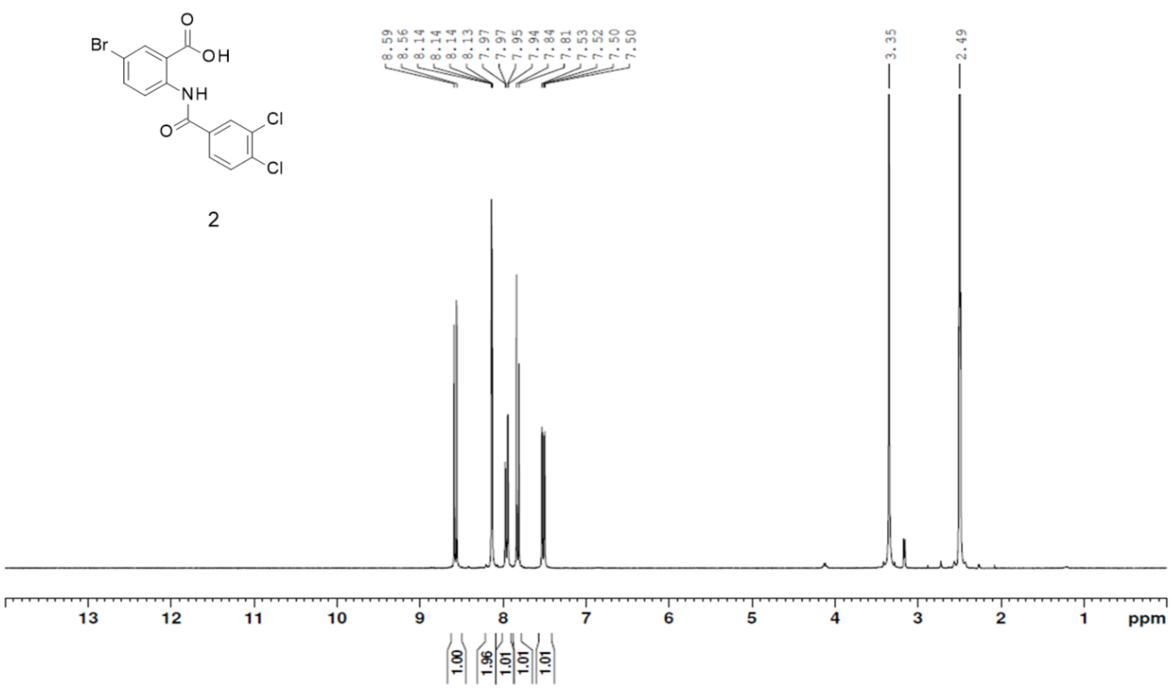

^{13}C NMR of compound 2 (75 MHz,DMSO-d6)

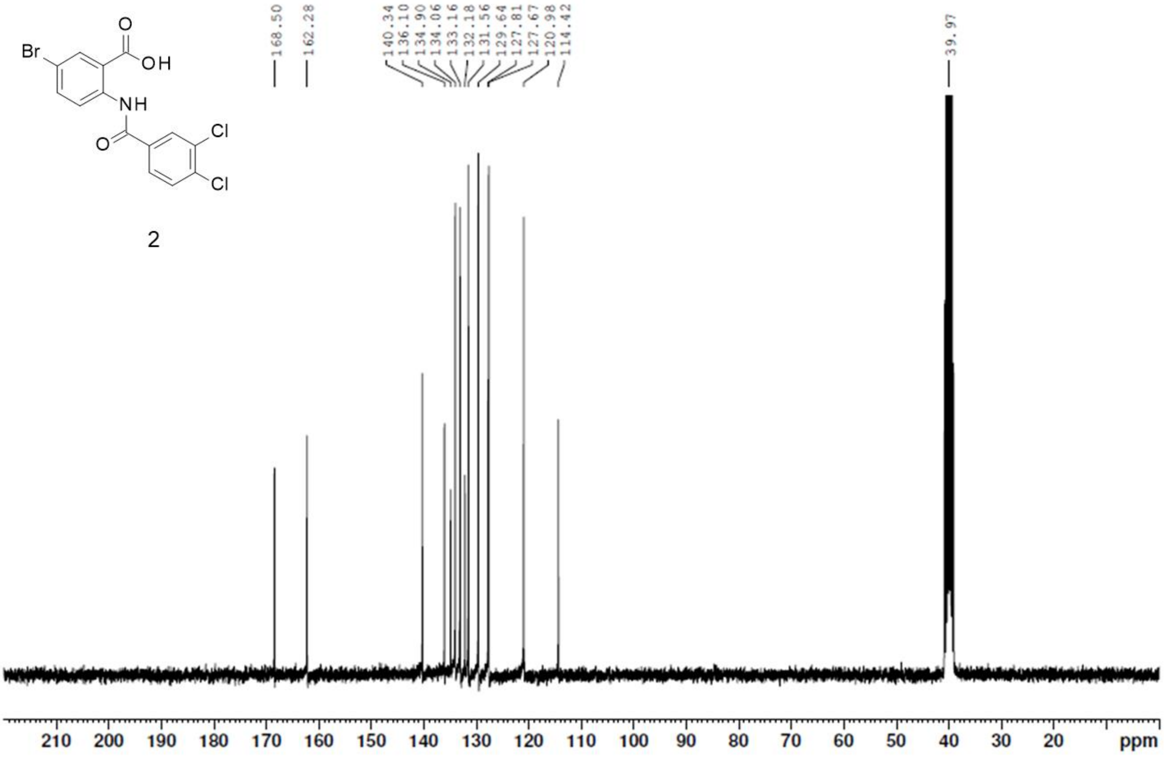

Figure S3: ^{1}H NMR and ^{13}C NMR spectra of compound **2**

Elemental Composition Report **Page 1**

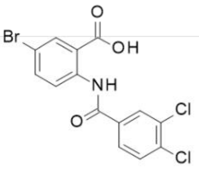

2

Single Mass Analysis
Tolerance = 100.0 mDa / DBE: min = -1.5, max = 500.0
Element prediction: Off
Number of isotope peaks used for i-FIT = 3

Monoisotopic Mass, Even Electron Ions
337 formula(e) evaluated with 55 results within limits (up to 10 best isotopic matches for each mass)
Elements Used:
C: 0-30 H: 0-40 N: 0-4 O: 0-6 Cl: 0-2 Br: 1-1

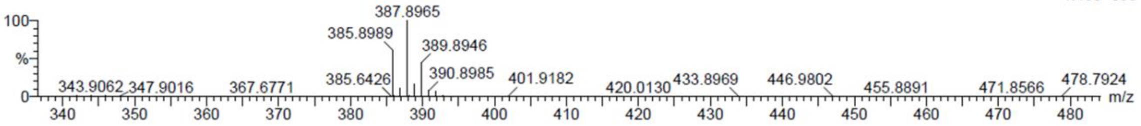

	Mass	Calc. Mass	mDa	PPM	DBE	i-FIT	Formula
Minimum:					-1.5		
Maximum:			100.0	5.0	500.0		
	385.8989	385.8986	0.3	0.8	10.5	6.7	C14 H7 N O3 Cl2 Br

Figure S4: HRMS spectrum of compound **2**

¹H NMR of compound 3 (300 MHz, MeOD-d4)

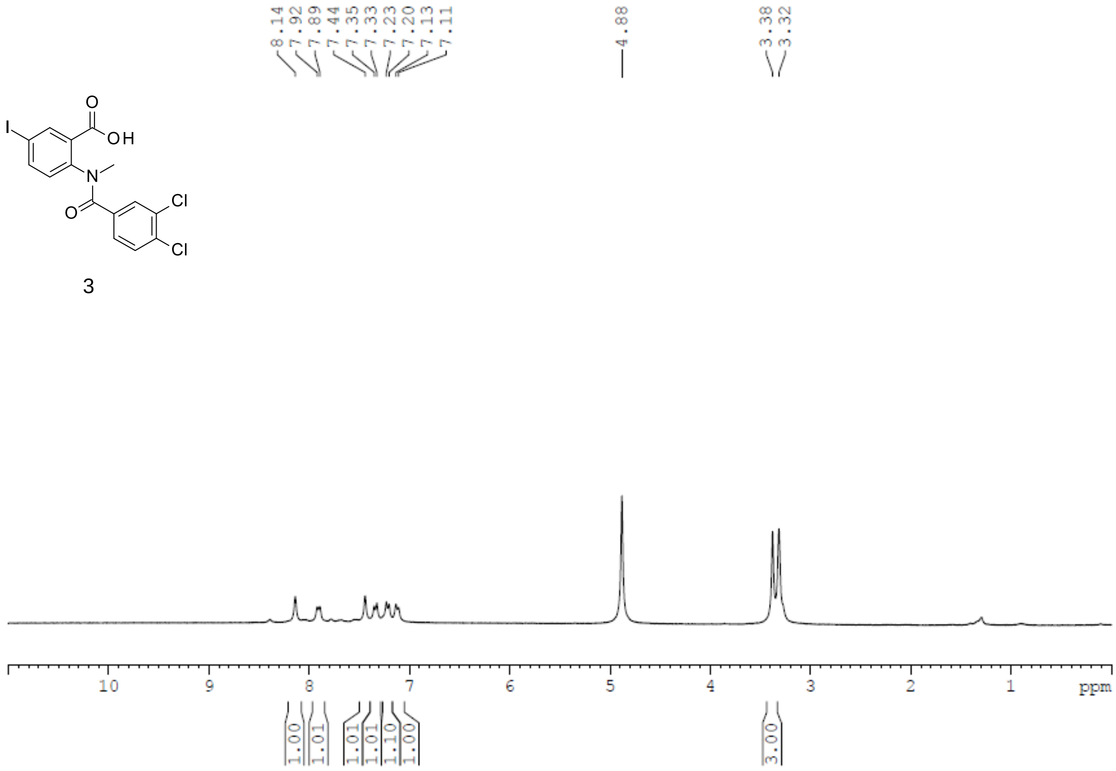

3

¹³C NMR of compound 3 (75 MHz, MeOD-d4)

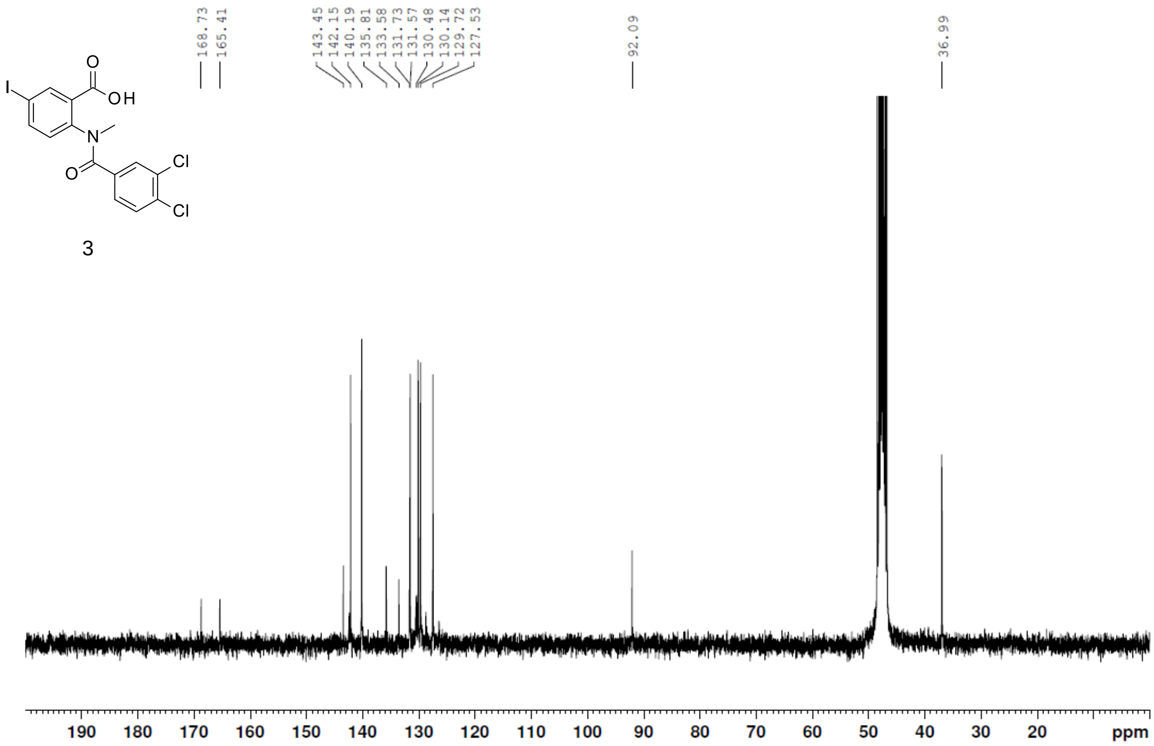

3

Figure S5: ¹H NMR and ¹³C NMR spectra of compound **3**

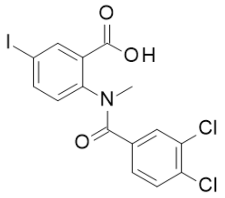

3

Elemental Composition Report Page 1

Single Mass Analysis
Tolerance = 20.0 PPM / DBE: min = -1.5, max = 80.0
Element prediction: Off
Number of isotope peaks used for i-FIT = 2

Monoisotopic Mass, Even Electron Ions
768 formula(e) evaluated with 11 results within limits (up to 1 best isotopic matches for each mass)
Elements Used:
C: 6-40 H: 2-36 N: 0-5 O: 0-7 Cl: 1-3 I: 0-1

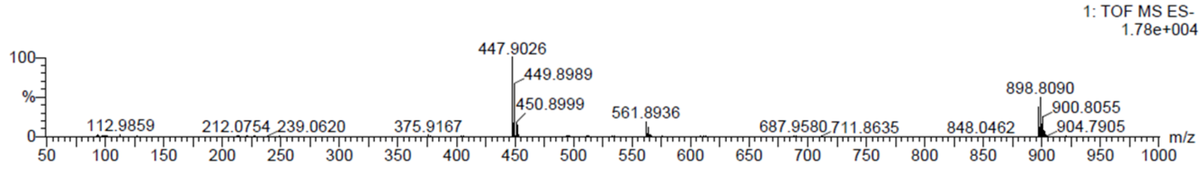

	Mass	Calc. Mass	mDa	PPM	DBE	i-FIT	Formula
Minimum:					-1.5		
Maximum:			10.0	20.0	80.0		
	447.9026	447.9004	2.2	4.9	10.5	2.1	C15 H9 N O3 Cl2 I

Figure S6: HRMS spectrum of compound **3**

^{1}H NMR of compound 4 (300 MHz, DMSO-d6)

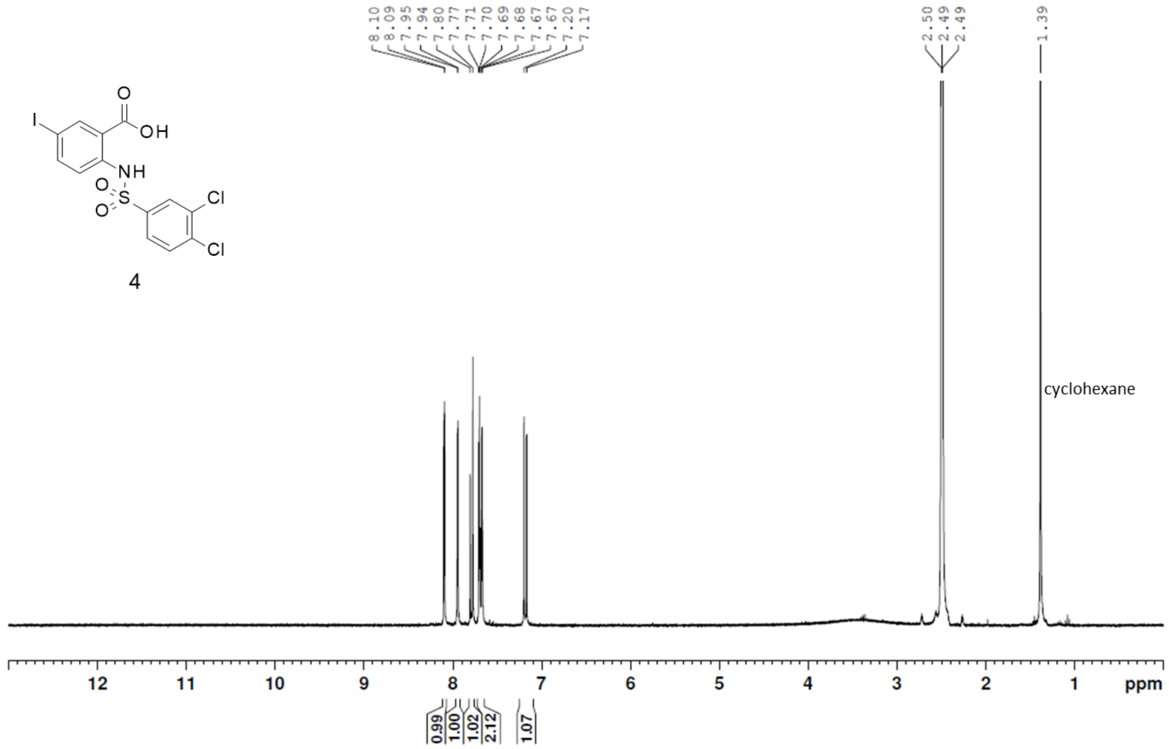

^{13}C NMR of compound 4 (75 MHz,DMSO-d6)

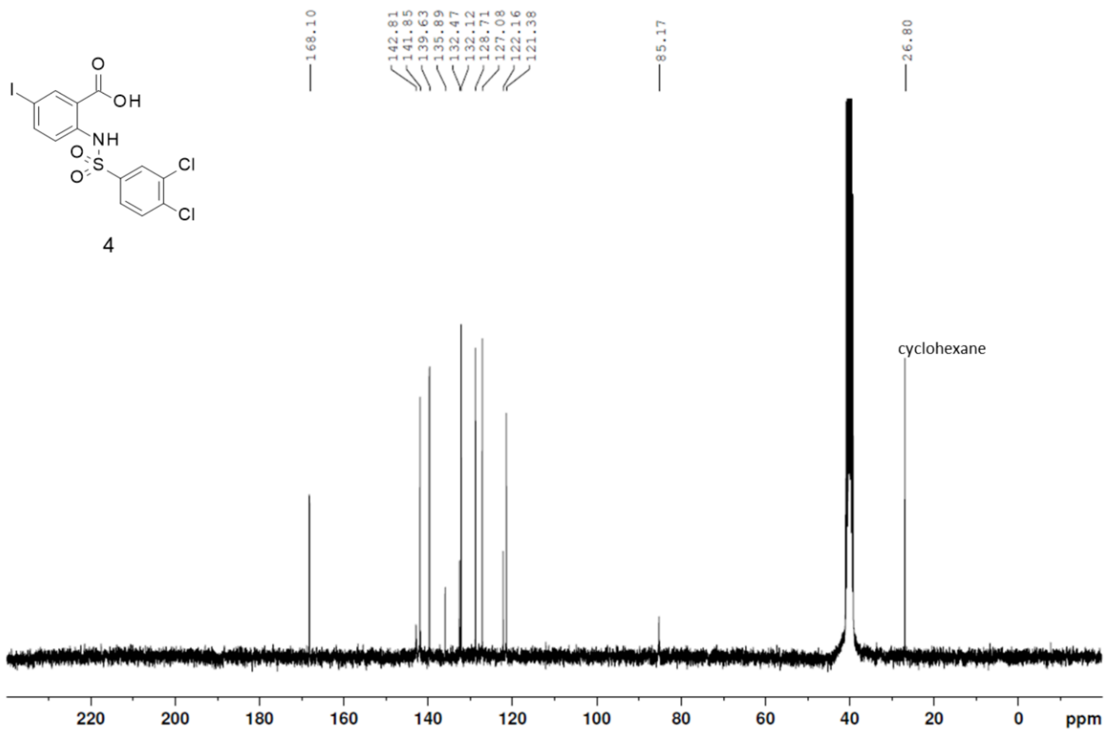

Figure S7: ^{1}H NMR and ^{13}C NMR spectra of compound **4**

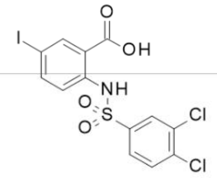

4

Elemental Composition Report

Single Mass Analysis

Tolerance = 10.0 PPM / DBE: min = -1.5, max = 50.0
Element prediction: Off
Number of isotope peaks used for i-FIT = 3

Monoisotopic Mass, Even Electron Ions
56 formula(e) evaluated with 1 results within limits (all results (up to 1000) for each mass)
Elements Used:
C: 1-17 H: 1-17 N: 1-2 O: 0-4 S: 1-1 Cl: 0-2 I: 0-1

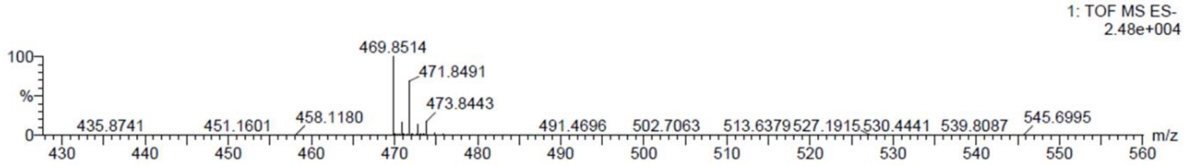

Minimum:				-1.5		
Maximum:		5.0	10.0	50.0		
Mass	Calc. Mass	mDa	PPM	DBE	i-FIT	Formula
469.8514	469.8518	-0.4	-0.9	9.5	6.1	C13 H7 N O4 S Cl2 I

Figure S8: HRMS spectrum of compound **4**

^{1}H NMR of compound 5 (300 MHz, DMSO-d6)

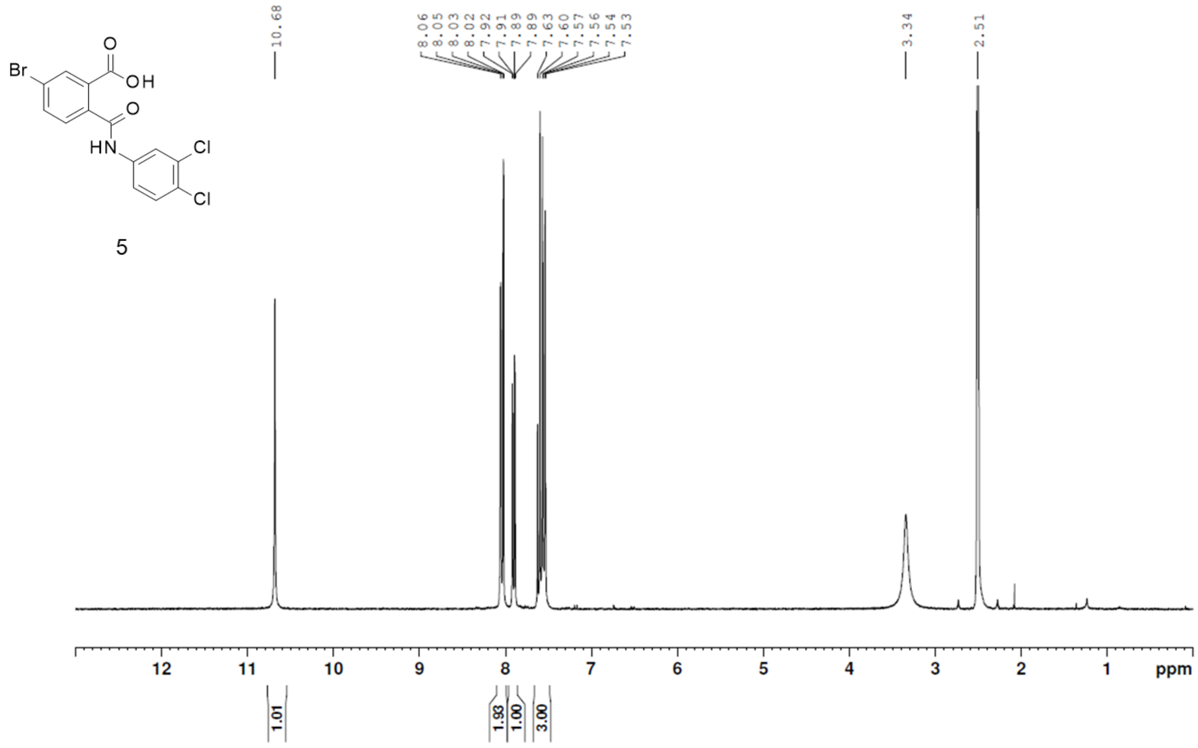

^{13}C NMR of compound 5 (75 MHz,DMSO-d6)

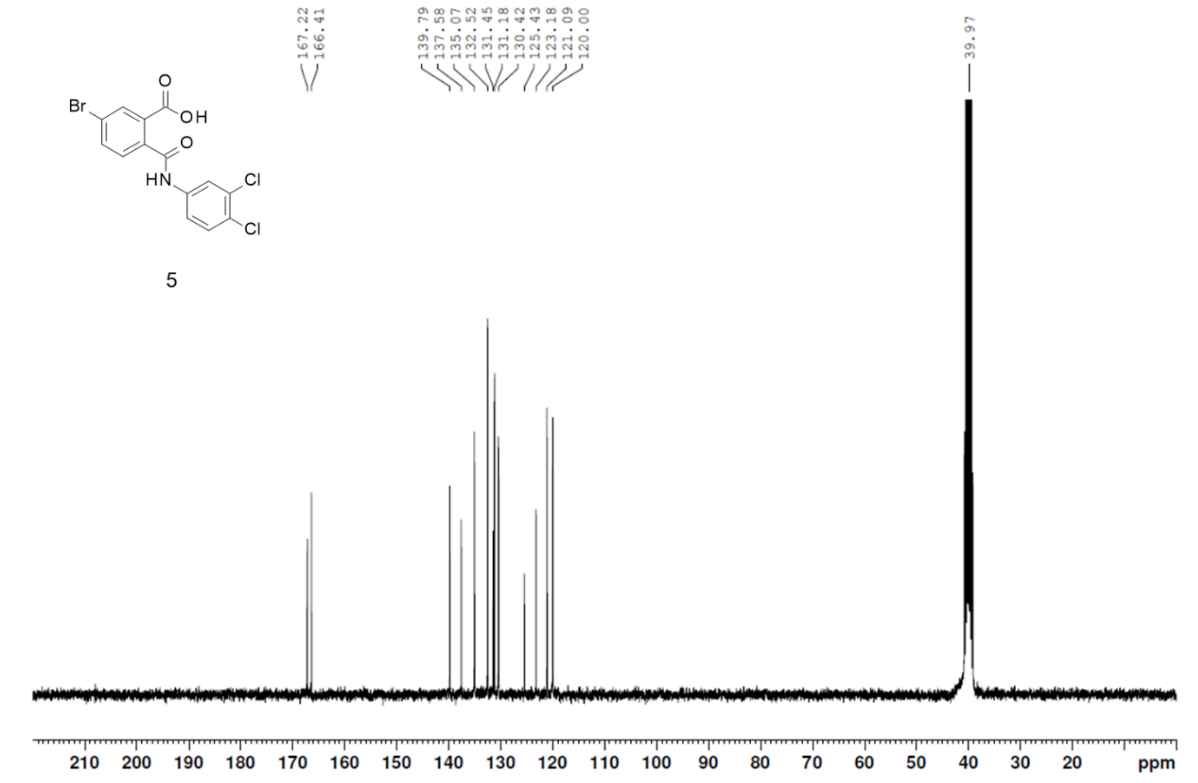

Figure S9: ^{1}H NMR and ^{13}C NMR spectra of compound **5**

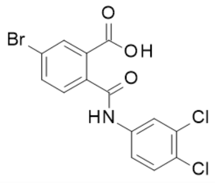

5

Elemental Composition Report **Page 1**

Single Mass Analysis
Tolerance = 20.0 PPM / DBE: min = -1.5, max = 50.0
Element prediction: Off
Number of isotope peaks used for i-FIT = 2

Monoisotopic Mass, Even Electron Ions
335 formula(e) evaluated with 7 results within limits (up to 1 best isotopic matches for each mass)
Elements Used:
C: 2-20 H: 2-36 N: 0-5 O: 0-7 Cl: 1-2 Br: 1-1 I: 0-1

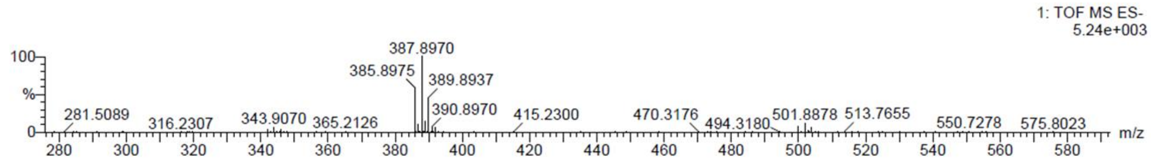

	Calc. Mass	mDa	PPM	DBE	i-FIT	Formula
Minimum:				-1.5		
Maximum:		10.0	20.0	50.0		
Mass	Calc. Mass	mDa	PPM	DBE	i-FIT	Formula
385.8975	385.8986	-1.1	-2.9	10.5	0.2	C14 H7 N O3 Cl2 Br

Figure S10: HRMS spectrum of compound **5**

^{1}H NMR of compound 6 (300 MHz, DMSO-d6)

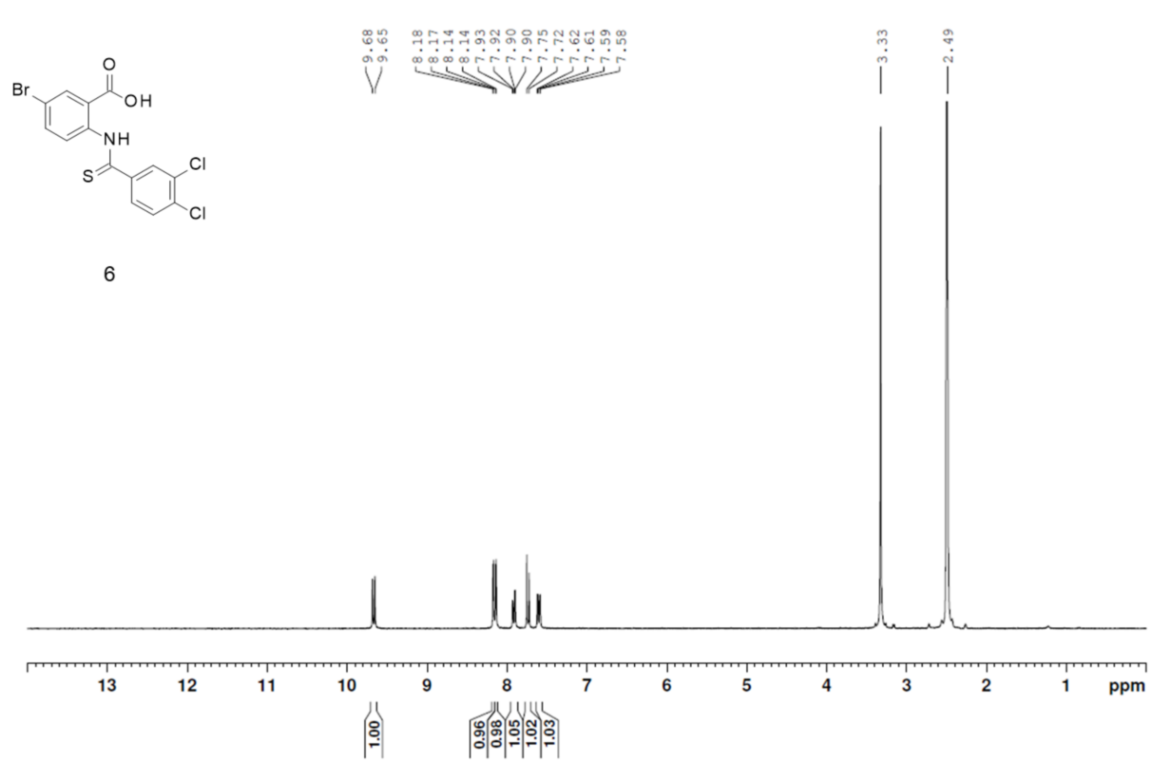

^{13}C NMR of compound 6 (75 MHz,DMSO-d6)

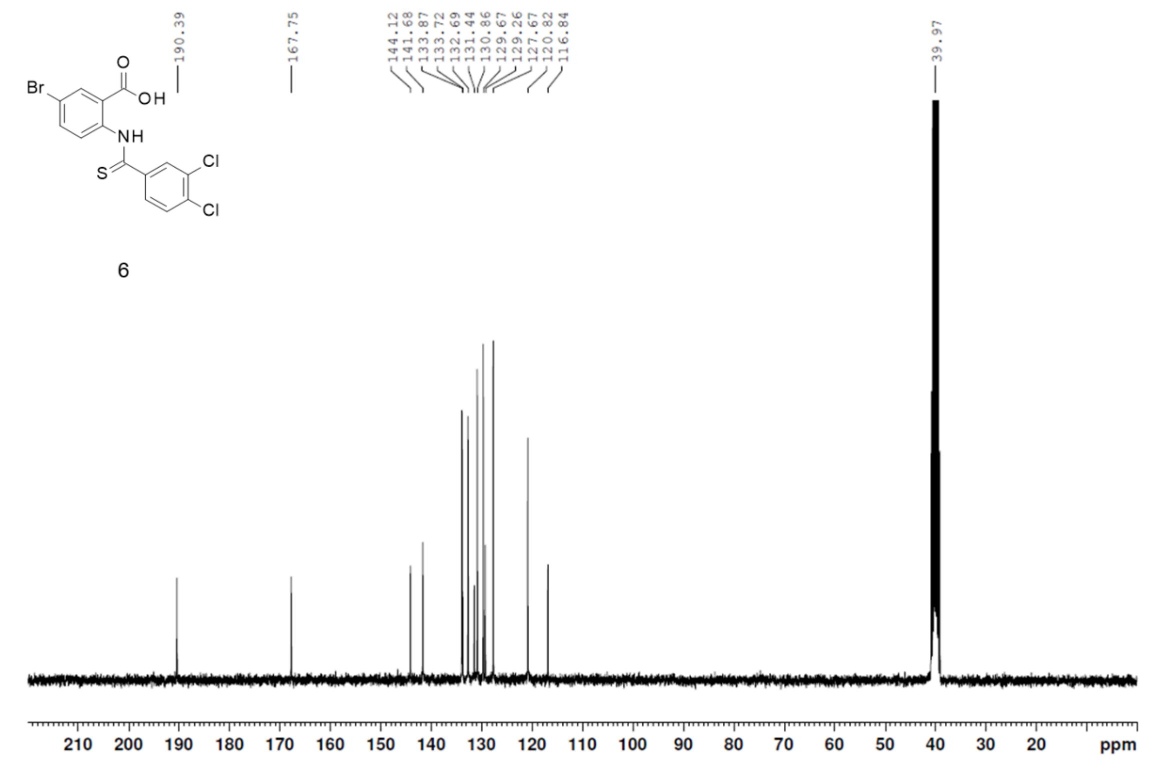

Figure S11: ^{1}H NMR and ^{13}C NMR spectra of compound **6**

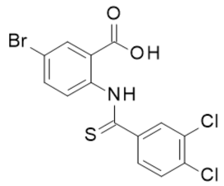

Elemental Composition Report

6

Single Mass Analysis
Tolerance = 20.0 PPM / DBE: min = -1.5, max = 50.0
Element prediction: Off
Number of isotope peaks used for i-FIT = 2

Monoisotopic Mass, Even Electron Ions
489 formula(e) evaluated with 16 results within limits (up to 1 best isotopic matches for each mass)
Elements Used:
C: 2-20 H: 2-36 N: 0-5 O: 0-7 S: 0-1 Cl: 1-2 Br: 1-1

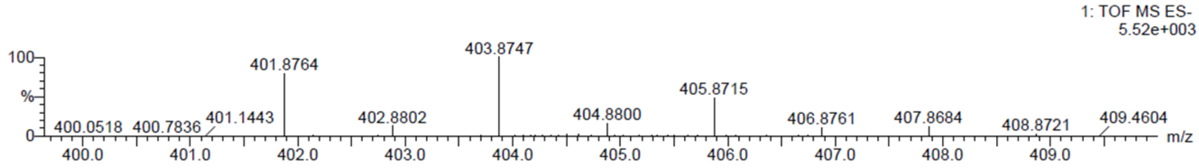

Minimum:				-1.5		
Maximum:		10.0	20.0	50.0		
Mass	Calc. Mass	mDa	PPM	DBE	i-FIT	Formula
401.8764	401.8758	0.6	1.5	10.5	1.8	C14 H7 N O2 S Cl2 Br

Figure S12: HRMS spectrum of compound **6**

^{1}H NMR of compound 7 (300 MHz, DMSO-d6)

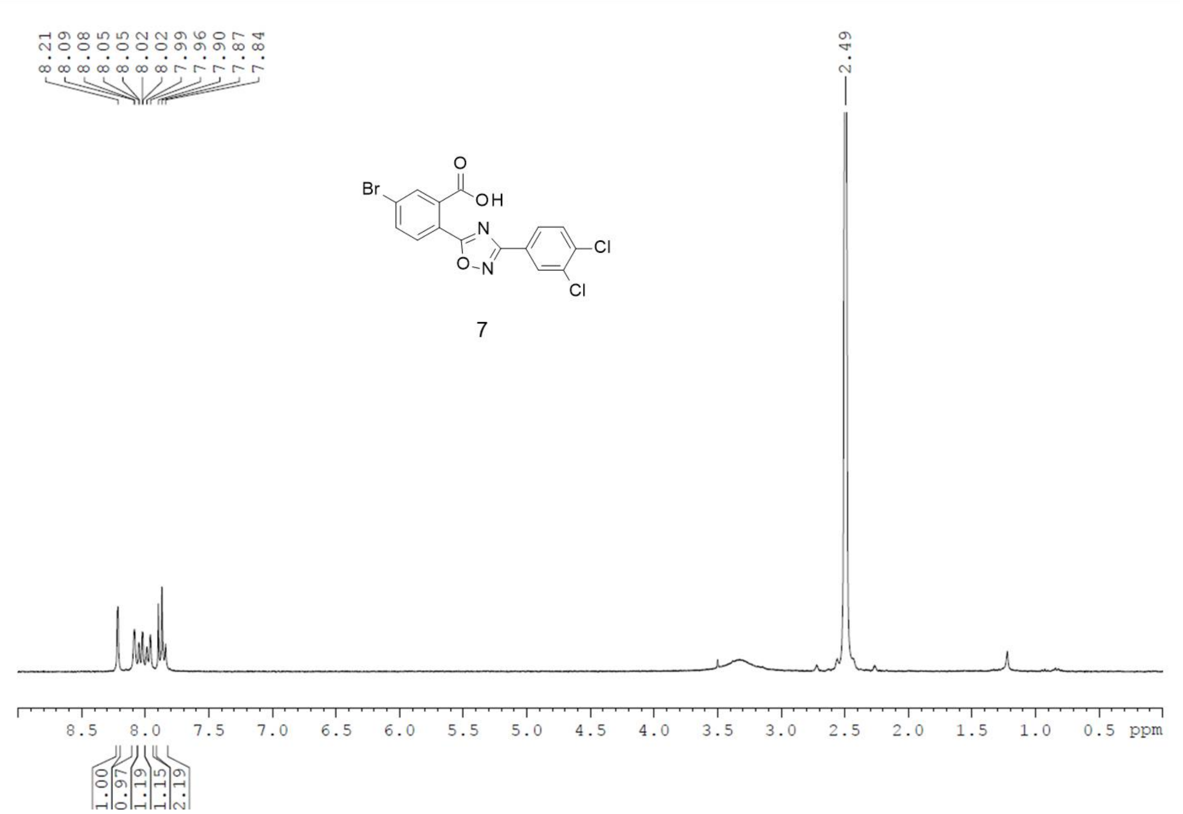

^{13}C NMR of compound 7 (75 MHz,DMSO-d6)

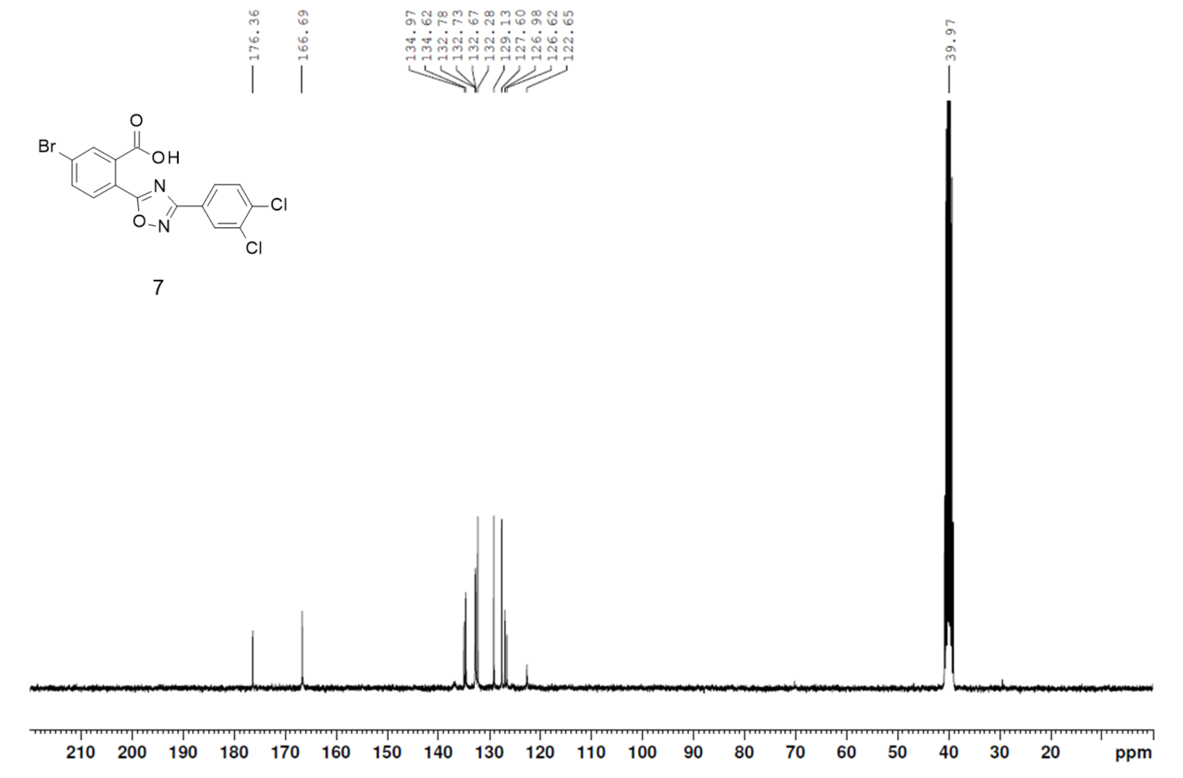

Figure S13: ^{1}H NMR and ^{13}C NMR spectra of compound **7**

Elemental Composition Report

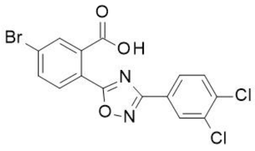

7

Single Mass Analysis

Tolerance = 100.0 mDa / DBE: min = -1.5, max = 500.0
Element prediction: Off
Number of isotope peaks used for i-FIT = 3

Monoisotopic Mass, Even Electron Ions
562 formula(e) evaluated with 159 results within limits (up to 10 best isotopic matches for each mass)
Elements Used:
C: 0-30 H: 0-40 N: 0-4 O: 0-6 Cl: 0-2 Br: 1-1 I: 0-1

1: TOF MS ES-
1.26e+005

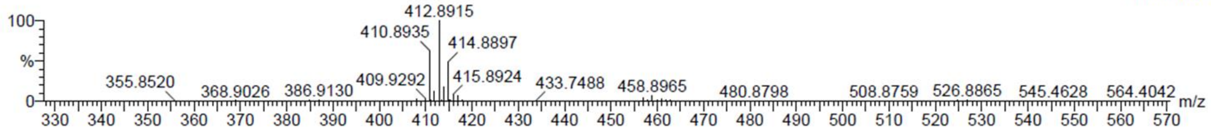

Minimum:				-1.5		
Maximum:		100.0	5.0	500.0		
Mass	Calc. Mass	mDa	PPM	DBE	i-FIT	Formula
410.8935	410.8939	-0.4	-1.0	12.5	59.1	C15 H6 N2 O3 Cl2 Br

Figure S14: HRMS spectrum of compound **7**

^{1}H NMR of compound 8 (300 MHz, DMSO-d6)

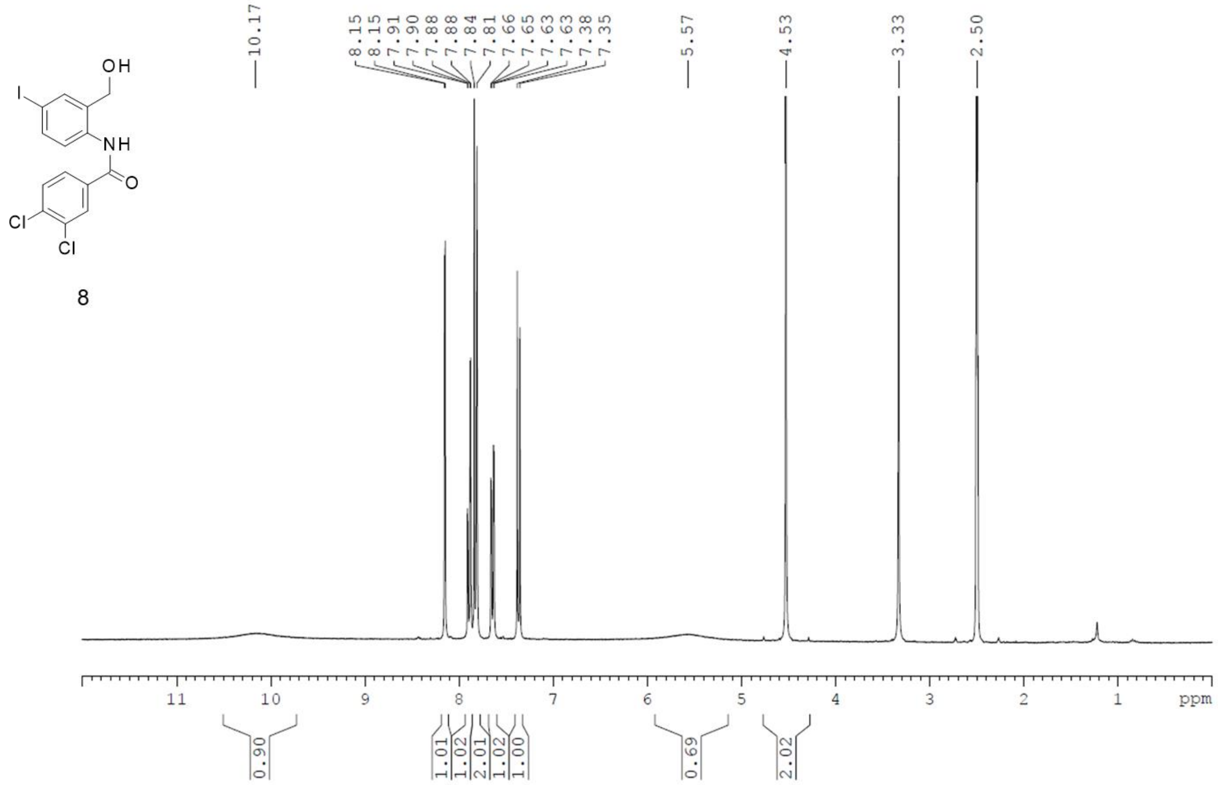

^{13}C NMR of compound 8 (75 MHz,DMSO-d6)

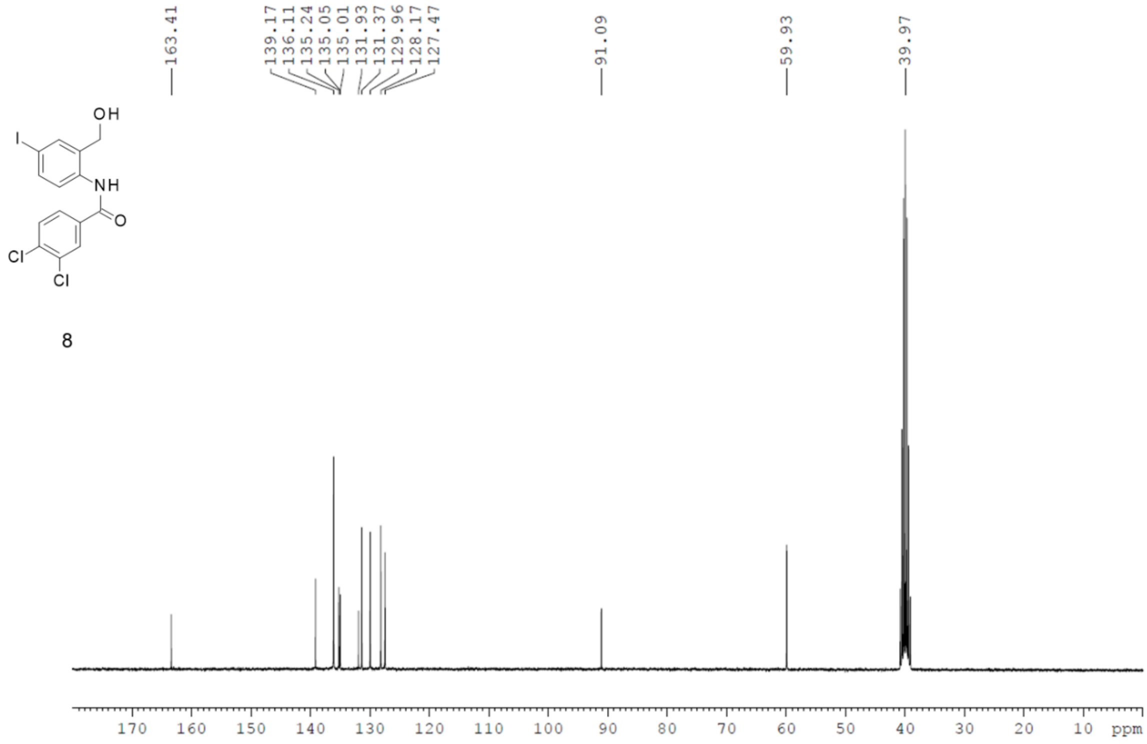

Figure S15: ^{1}H NMR and ^{13}C NMR spectra of compound **8**

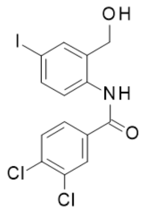

8

Elemental Composition Report

Single Mass Analysis

Tolerance = 20.0 PPM / DBE: min = -1.5, max = 50.0
Element prediction: Off
Number of isotope peaks used for i-FIT = 2

Monoisotopic Mass, Even Electron Ions
391 formula(e) evaluated with 10 results within limits (up to 1 best isotopic matches for each mass)
Elements Used:
C: 2-20 H: 2-36 N: 0-5 O: 0-7 Cl: 1-2 I: 0-1

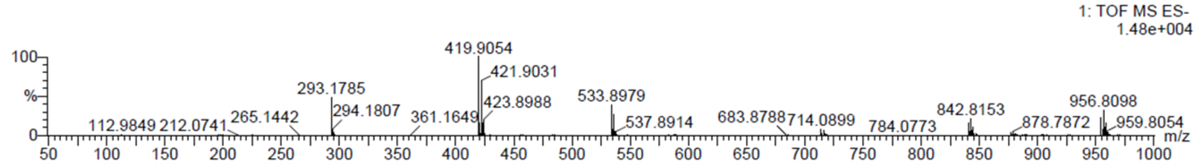

	Mass	Calc. Mass	mDa	PPM	DBE	i-FIT	Formula
Minimum:					-1.5		
Maximum:			10.0	20.0	50.0		
	419.9054	419.9055	-0.1	-0.2	9.5	0.2	C14 H9 N O2 Cl2 I

Figure S16: HRMS spectrum of compound **8**

^{1}H NMR of compound 9 (300 MHz, DMSO-d6)

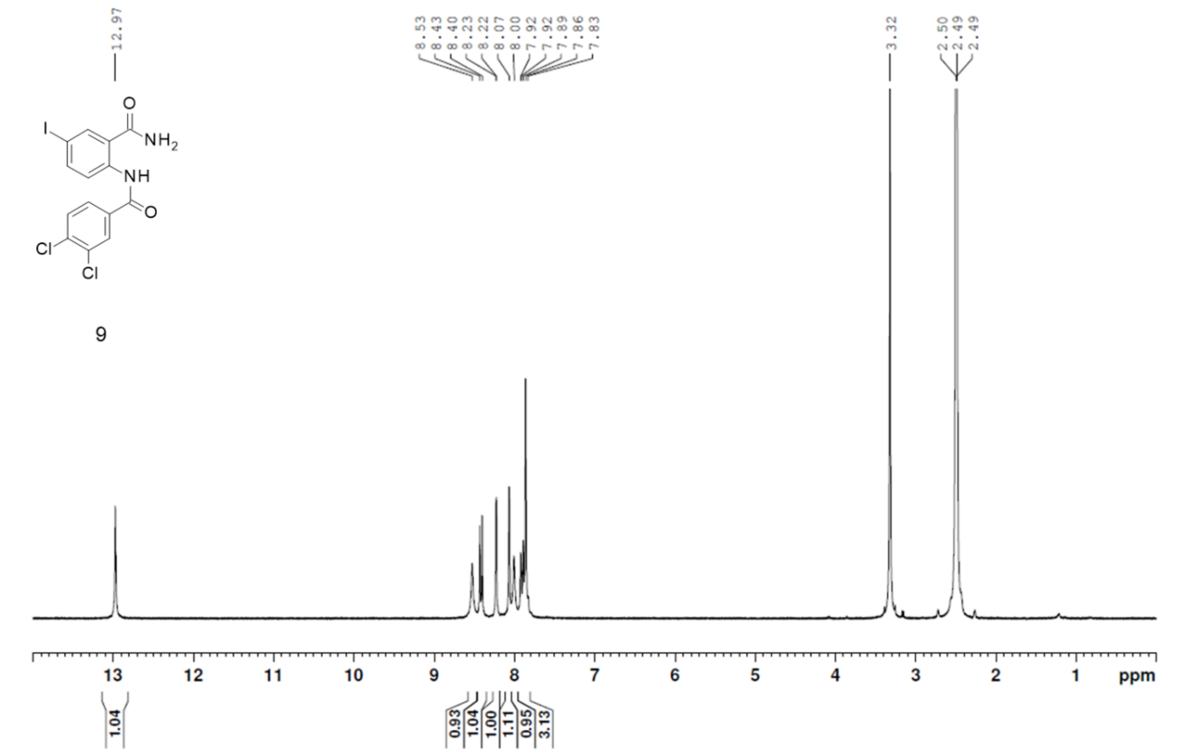

^{13}C NMR of compound 9 (75 MHz,DMSO-d6)

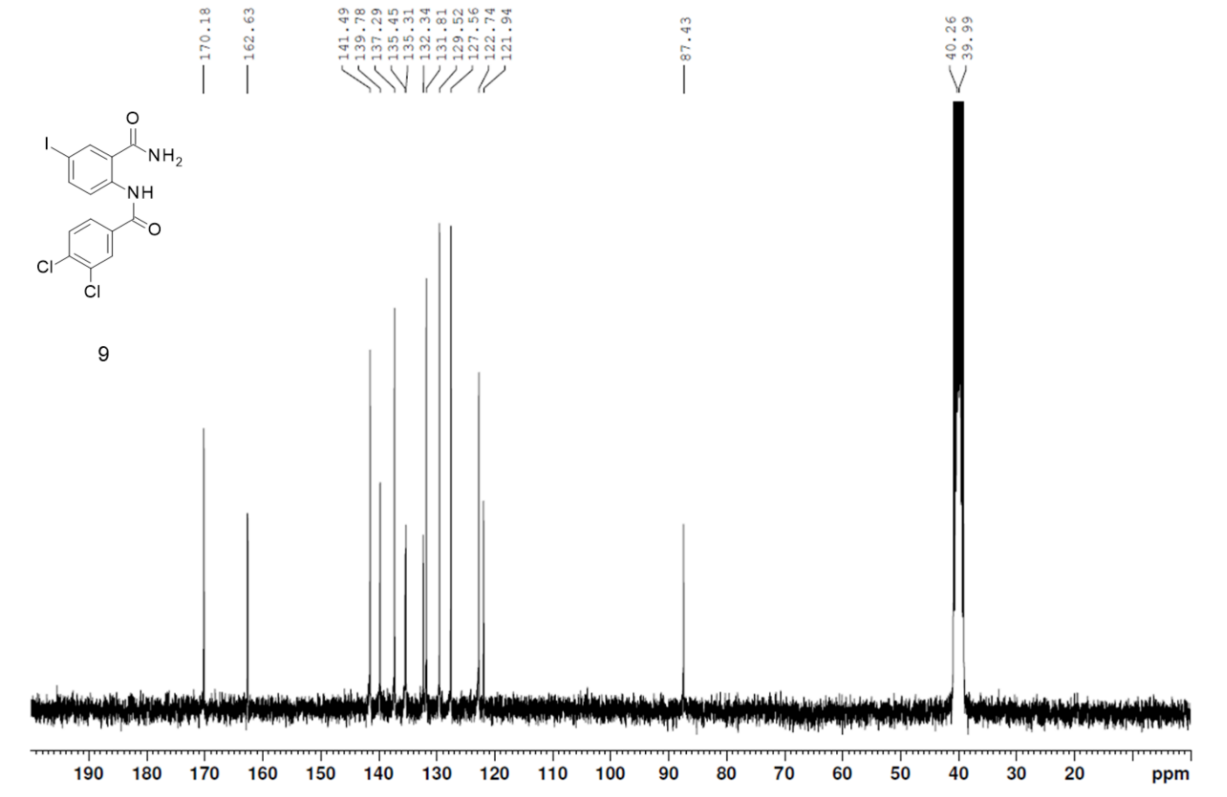

Figure S17: ^{1}H NMR and ^{13}C NMR spectra of compound **9**

Elemental Composition Report

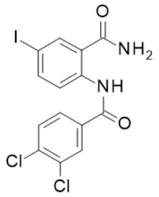

9

Single Mass Analysis

Tolerance = 20.0 PPM / DBE: min = -1.5, max = 80.0
Element prediction: Off
Number of isotope peaks used for i-FIT = 2

Monoisotopic Mass, Even Electron Ions
635 formula(e) evaluated with 12 results within limits (up to 1 closest results for each mass)
Elements Used:
C: 6-40 H: 2-36 N: 0-4 O: 0-7 Cl: 1-3 I: 0-1

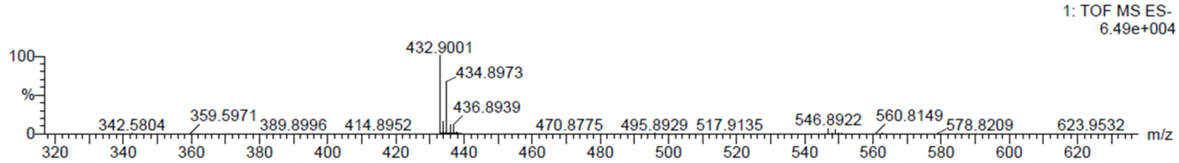

Minimum:				-1.5		
Maximum:		10.0	20.0	80.0		
Mass	Calc. Mass	mDa	PPM	DBE	i-FIT	Formula
432.9001	432.9008	-0.7	-1.6	10.5	5.6	C14 H8 N2 O2 Cl2 I

Figure S18: HRMS spectrum of compound **9**

^{1}H NMR of compound 10 (300 MHz, DMSO-d6)

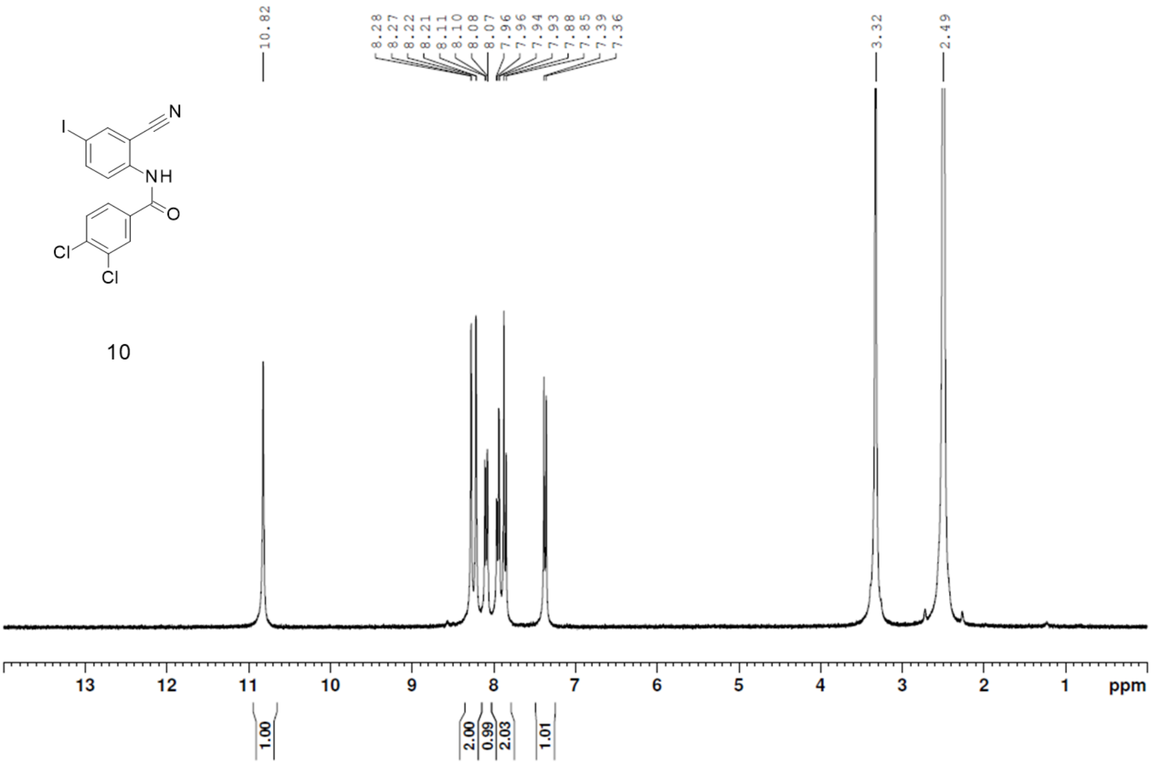

^{13}C NMR of compound 10 (75 MHz,DMSO-d6)

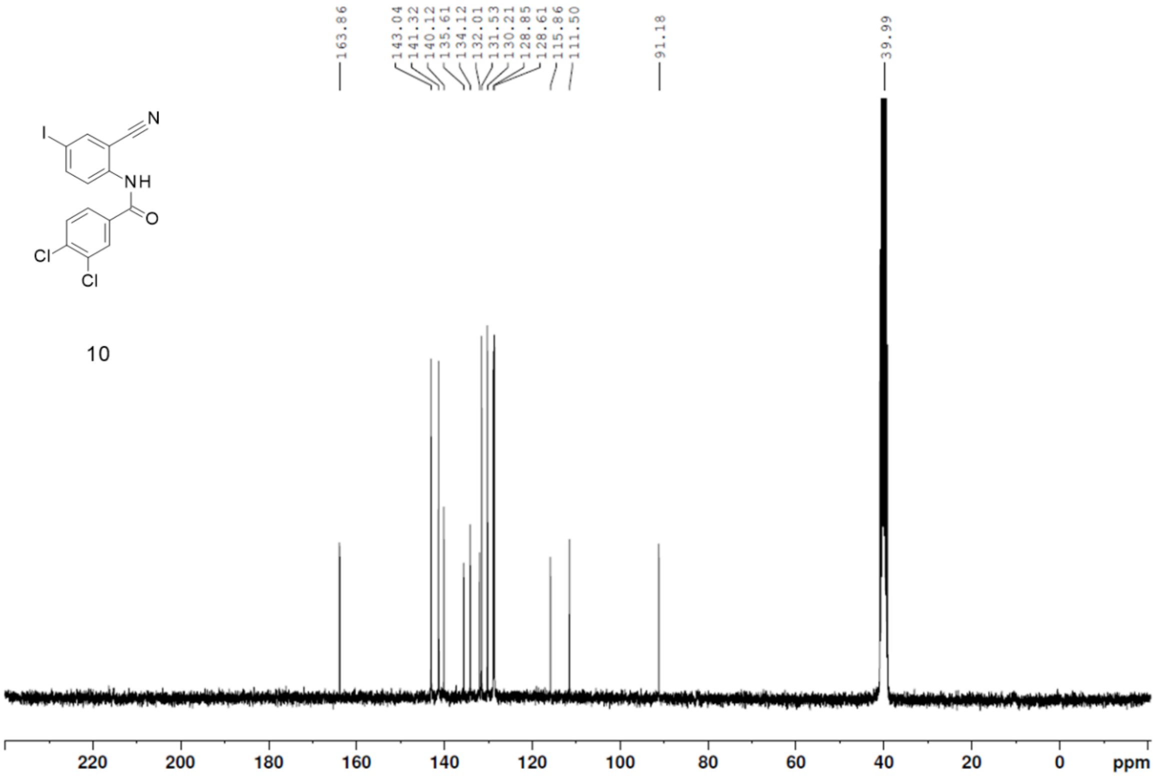

Figure S19: ^{1}H NMR and ^{13}C NMR spectra of compound **10**

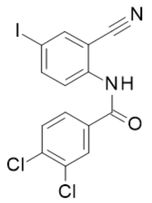

10

Elemental Composition Report **Page 1**

Single Mass Analysis
Tolerance = 20.0 PPM / DBE: min = -1.5, max = 80.0
Element prediction: Off
Number of isotope peaks used for i-FIT = 2

Monoisotopic Mass, Even Electron Ions
603 formula(e) evaluated with 12 results within limits (up to 1 closest results for each mass)
Elements Used:
C: 6-40 H: 2-36 N: 0-4 O: 0-7 Cl: 1-3 I: 0-1

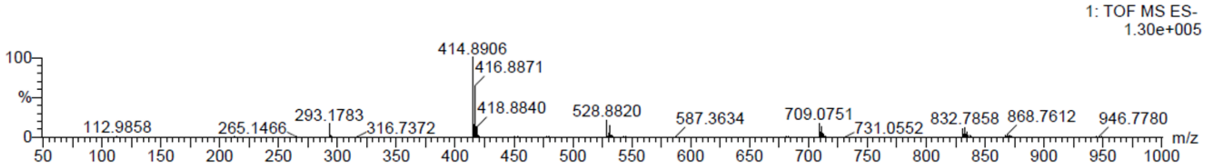

Minimum:				-1.5		
Maximum:		10.0	20.0	80.0		
Mass	Calc. Mass	mDa	PPM	DBE	i-FIT	Formula
414.8906	414.8902	0.4	1.0	11.5	1.1	C14 H6 N2 O Cl2 I

Figure S20: HRMS spectrum of compound **10**

^{1}H NMR of compound 11 (300 MHz, DMSO-d6)

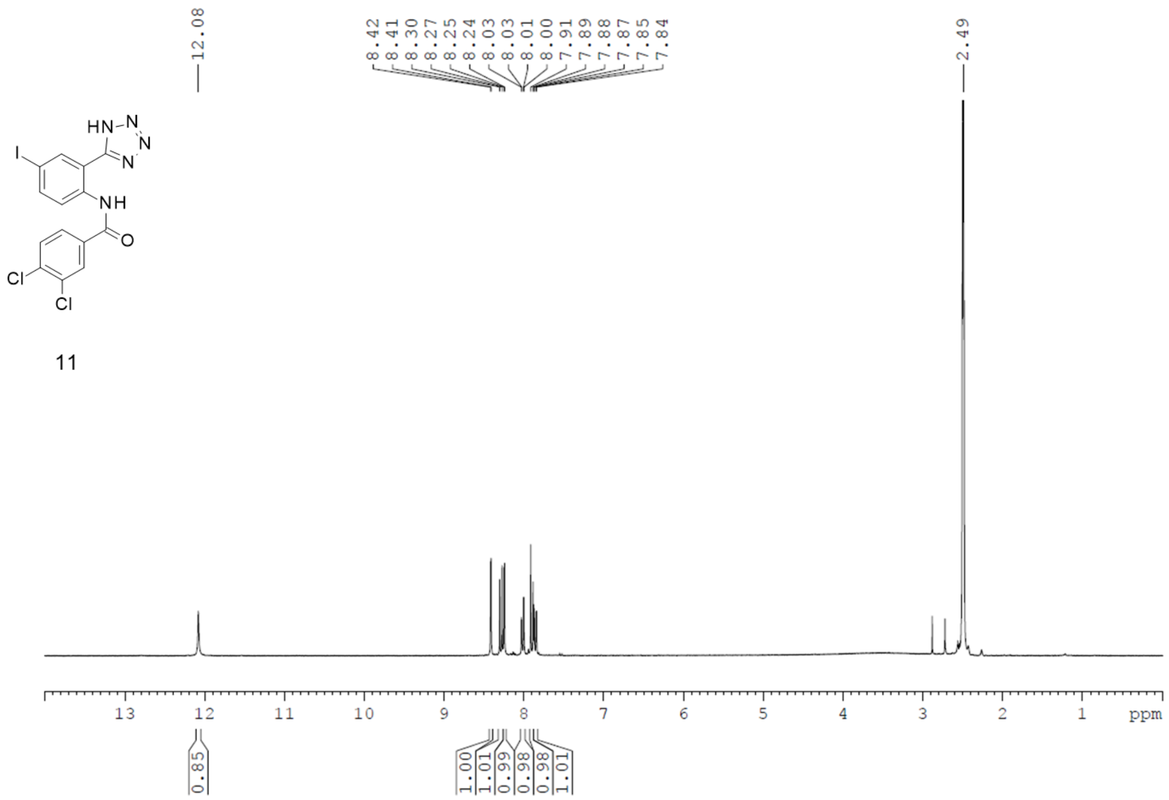

^{13}C NMR of compound 11 (75 MHz,DMSO-d6)

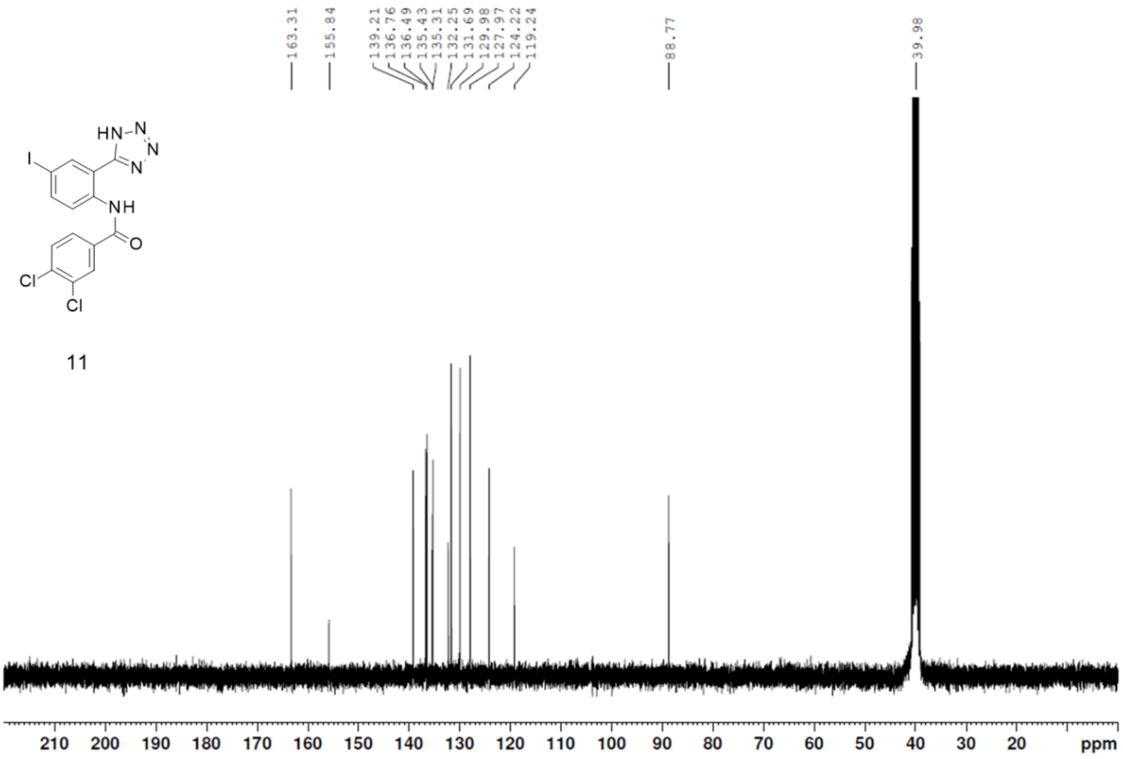

Figure S21: ^{1}H NMR and ^{13}C NMR spectra of compound **11**

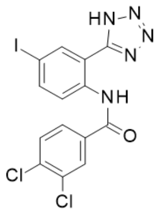

11

Elemental Composition Report

Single Mass Analysis

Tolerance = 20.0 PPM / DBE: min = -1.5, max = 80.0
Element prediction: Off
Number of isotope peaks used for i-FIT = 2

Monoisotopic Mass, Even Electron Ions
783 formula(e) evaluated with 17 results within limits (up to 1 closest results for each mass)
Elements Used:
C: 6-40 H: 2-36 N: 0-5 O: 0-7 Cl: 1-3 I: 0-1

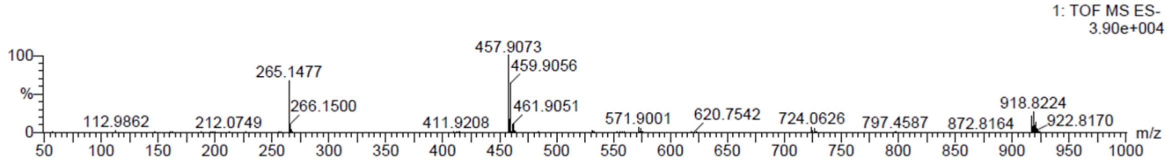

Minimum:				-1.5		
Maximum:		10.0	20.0	80.0		
Mass	Calc. Mass	mDa	PPM	DBE	i-FIT	Formula
457.9073	457.9072	0.1	0.2	12.5	8.0	C14 H7 N5 O Cl2 I

Figure S22: HRMS spectrum of compound **11**

^{1}H NMR of compound 12 (300 MHz, DMSO-d6)

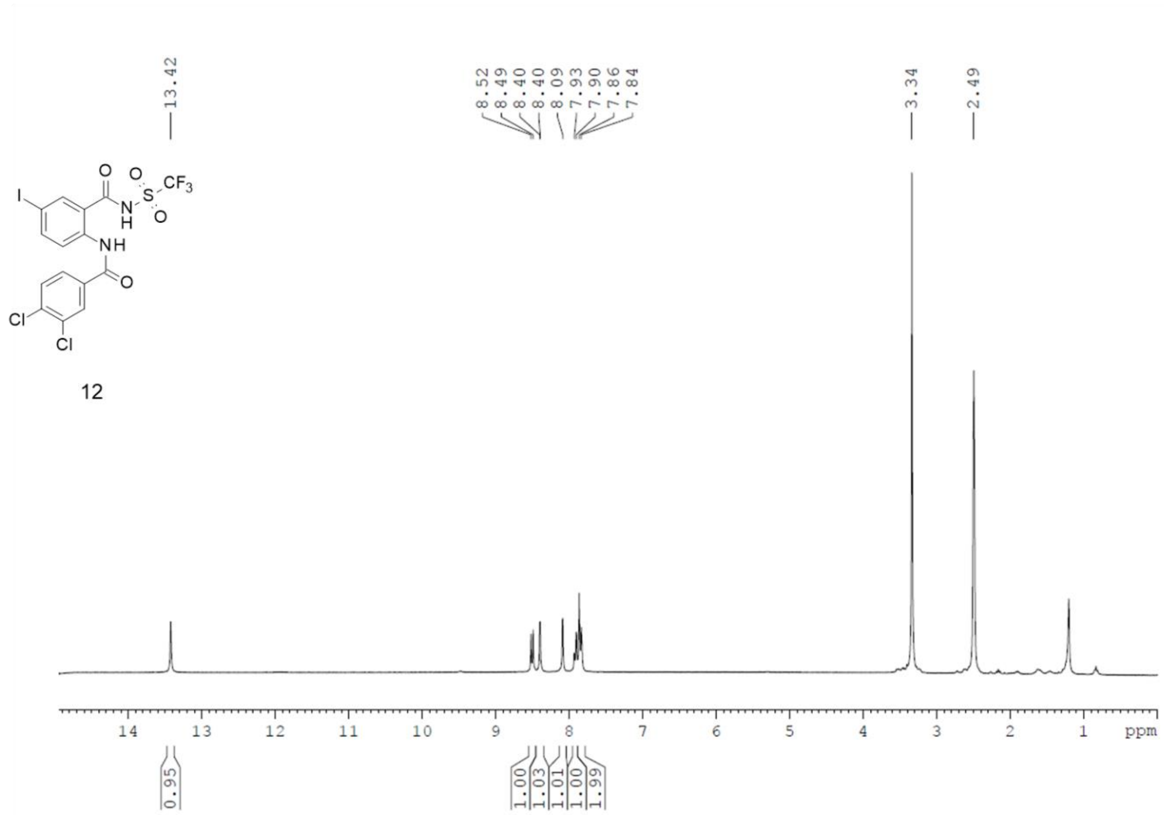

^{13}C NMR of compound 12 (75 MHz,DMSO-d6)

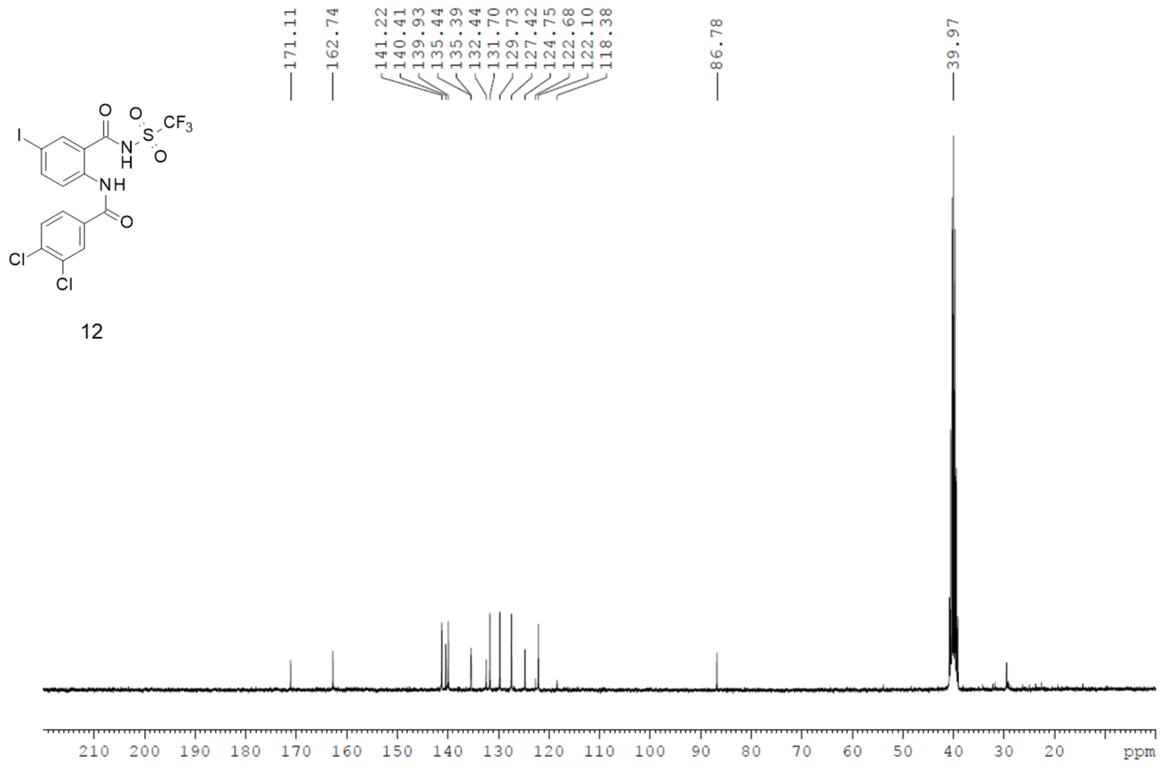

Figure S23: ^{1}H NMR and ^{13}C NMR spectra of compound **12**

Elemental Composition Report

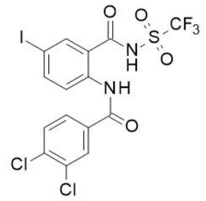

12

Page 1

Single Mass Analysis
Tolerance = 10.0 PPM / DBE: min = -1.5, max = 50.0
Element prediction: Off
Number of isotope peaks used for i-FIT = 3

Monoisotopic Mass, Even Electron Ions
11796 formula(e) evaluated with 19 results within limits (up to 5 closest results for each mass)
Elements Used:
C: 15-15 H: 0-60 N: 0-10 O: 0-15 F: 1-3 S: 0-3 Cl: 0-2 I: 0-1

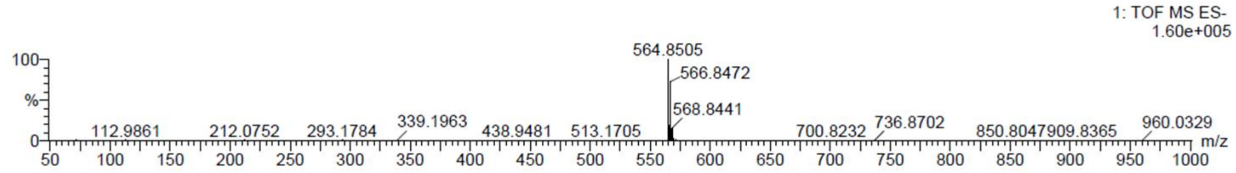

Minimum:				-1.5		
Maximum:		5.0	10.0	50.0		
Mass	Calc. Mass	mDa	PPM	DBE	i-FIT	Formula
564.8505	564.8500	0.5	0.9	10.5	35.5	C15 H7 N2 O4 F3 S Cl2 I

Figure S24: HRMS spectrum of compound **12**

^{1}H NMR of compound 13 (300 MHz, DMSO-d6)

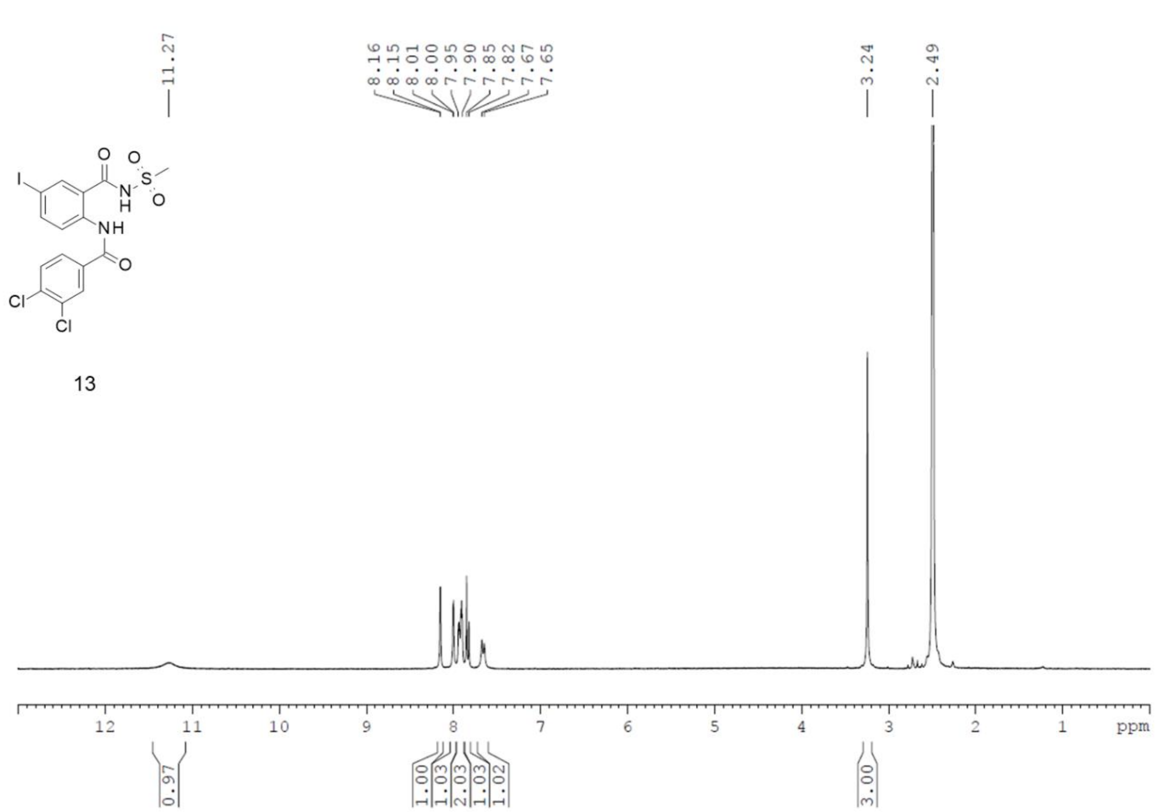

^{13}C NMR of compound 13 (75 MHz,DMSO-d6)

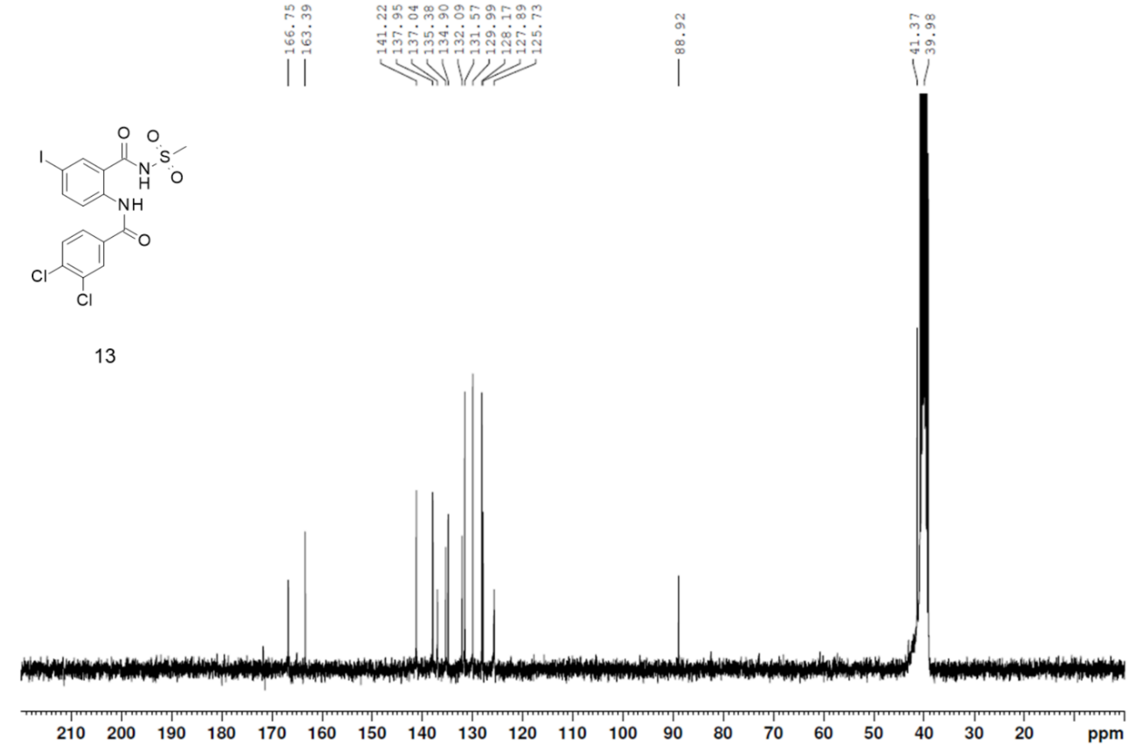

Figure S25: ^{1}H NMR and ^{13}C NMR spectra of compound **13**

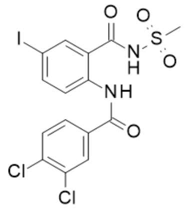

13

Elemental Composition Report **Page 1**

Single Mass Analysis
Tolerance = 20.0 PPM / DBE: min = -1.5, max = 80.0
Element prediction: Off
Number of isotope peaks used for i-FIT = 2

Monoisotopic Mass, Even Electron Ions
1636 formula(e) evaluated with 30 results within limits (up to 1 best isotopic matches for each mass)
Elements Used:
C: 6-40 H: 2-36 N: 0-5 O: 0-7 S: 0-1 Cl: 1-3 I: 0-1

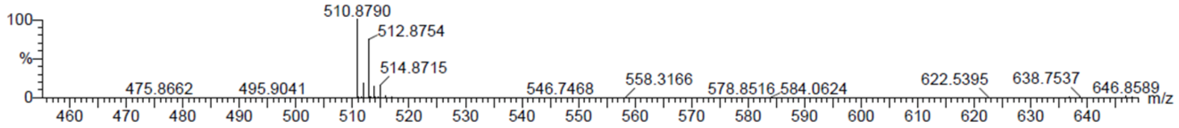

Minimum:				-1.5		
Maximum:		10.0	20.0	80.0		
Mass	Calc. Mass	mDa	PPM	DBE	i-FIT	Formula
510.8790	510.8783	0.7	1.4	10.5	0.2	C15 H10 N2 O4 S Cl2 I

Figure S26: HRMS spectrum of compound **13**

^1H NMR of compound 14 (300 MHz, DMSO-d6)

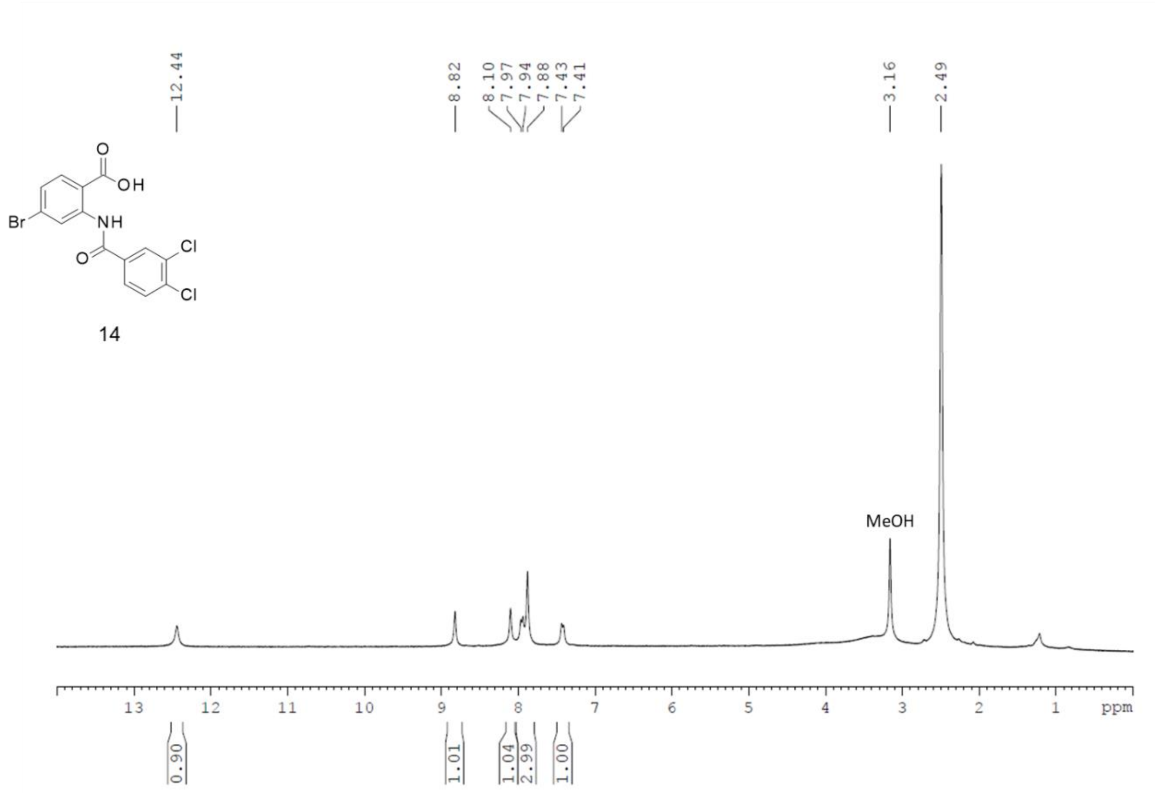

^{13}C JMOD NMR of compound 14 (75 MHz,DMSO-d6)

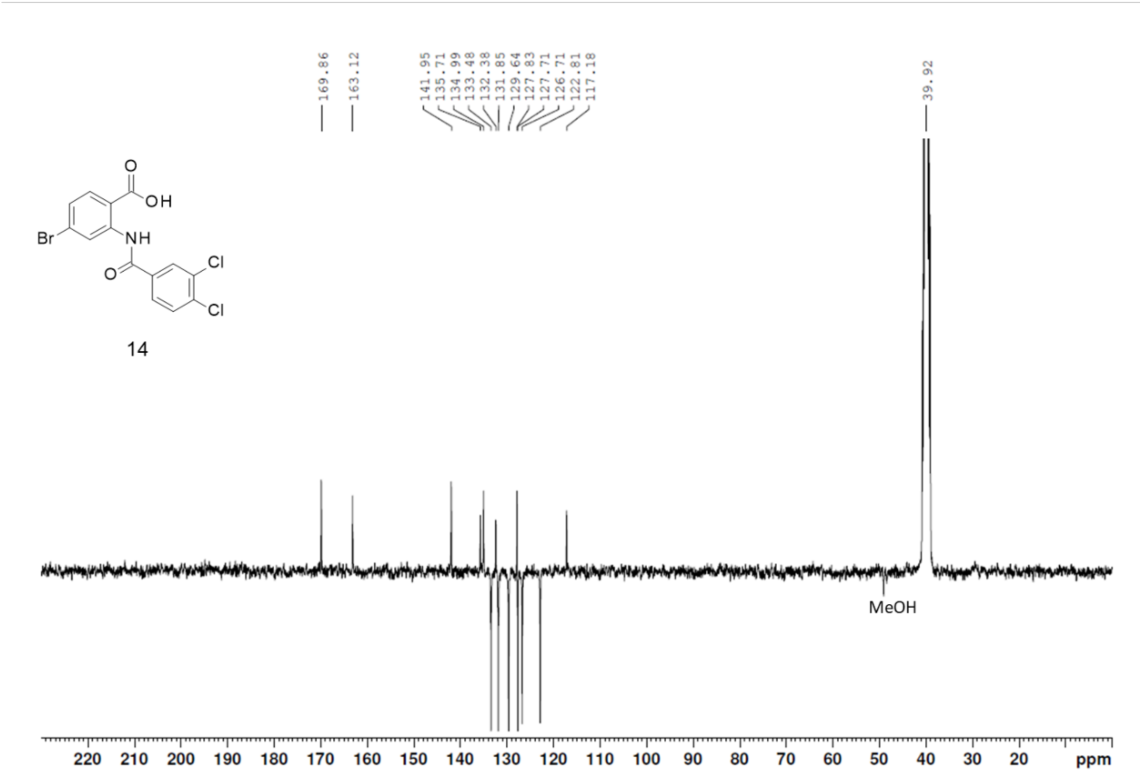

Figure S27: ^1H NMR and ^{13}C NMR spectra of compound **14**

Elemental Composition Report

Single Mass Analysis
Tolerance = 10.0 PPM / DBE: min = -1.5, max = 50.0
Element prediction: Off
Number of isotope peaks used for i-FIT = 3

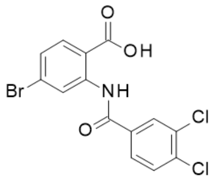

14

Monoisotopic Mass, Even Electron Ions
2876 formula(e) evaluated with 6 results within limits (up to 5 closest results for each mass)
Elements Used:
C: 10-50 H: 0-60 N: 0-10 O: 0-15 Cl: 0-2 Br: 0-1 I: 0-1

1: TOF MS ES-
5.20e+003

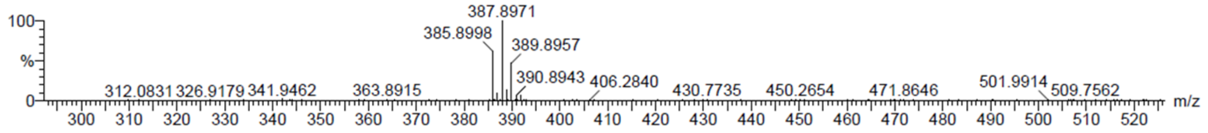

Mass	Calc. Mass	mDa	PPM	DBE	i-FIT	Formula
Minimum:				-1.5		
Maximum:		5.0	10.0	50.0		
385.8998	385.8986	1.2	3.1	10.5	1.8	C14 H7 N O3 Cl2 Br

Figure S28: HRMS spectrum of compound **14**

^{1}H NMR of compound 15 (300 MHz, DMSO-d6)

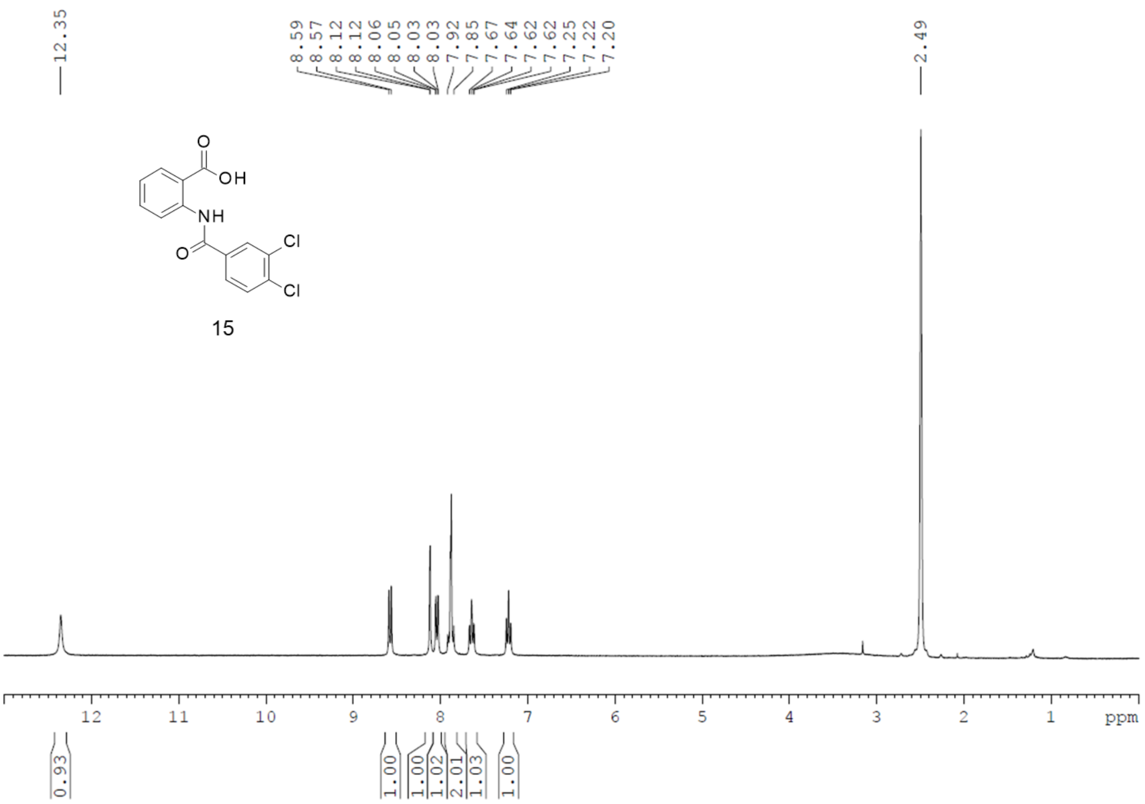

^{13}C JMOD NMR of compound 15 (75 MHz,DMSO-d6)

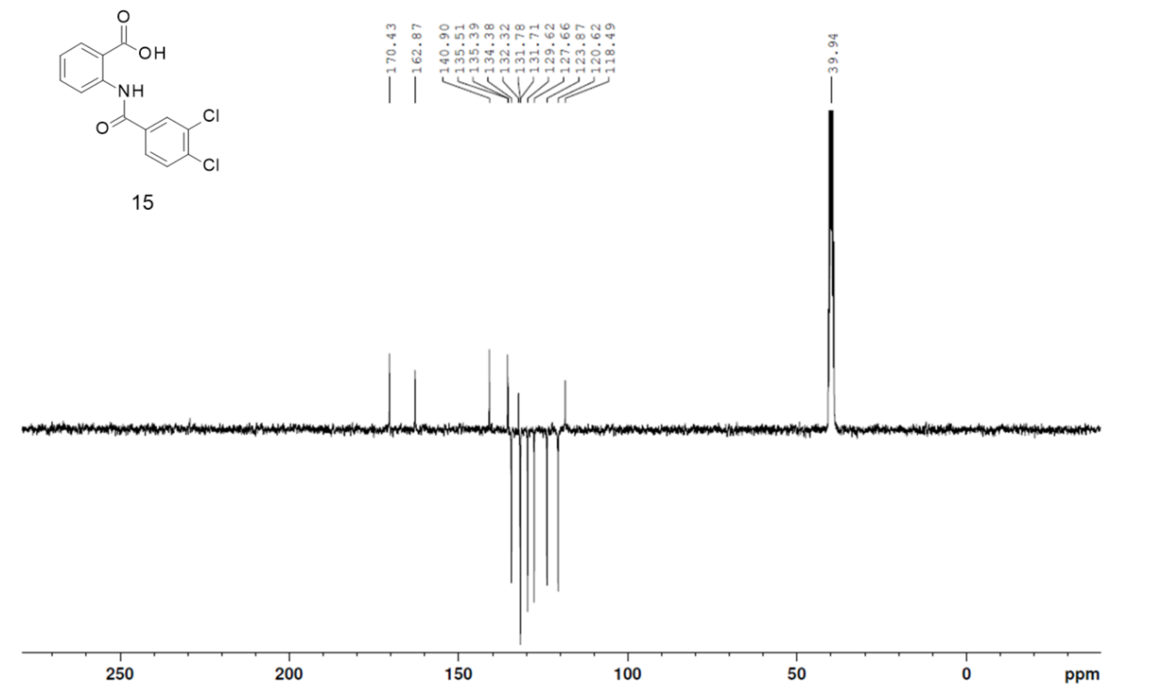

Figure S29: ^{1}H NMR and ^{13}C NMR spectra of compound **15**

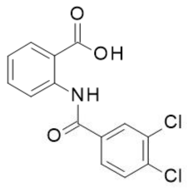

15

Elemental Composition Report **Page 1**

Single Mass Analysis
Tolerance = 20.0 PPM / DBE: min = -1.5, max = 50.0
Element prediction: Off
Number of isotope peaks used for i-FIT = 2

Monoisotopic Mass, Even Electron Ions
343 formula(e) evaluated with 5 results within limits (up to 1 best isotopic matches for each mass)
Elements Used:
C: 2-20 H: 2-36 N: 0-5 O: 0-7 Cl: 1-2 I: 0-1

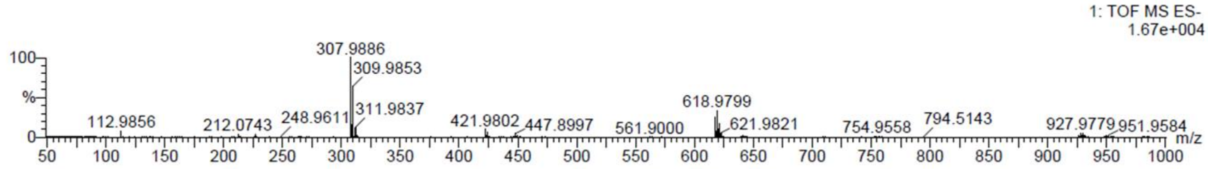

Minimum:				-1.5		
Maximum:		10.0	20.0	50.0		
Mass	Calc. Mass	mDa	PPM	DBE	i-FIT	Formula
307.9886	307.9881	0.5	1.6	10.5	1.1	C14 H8 N O3 Cl2

Figure S30: HRMS spectrum of compound **15**

^{1}H NMR of compound 16 (300 MHz, DMSO-d6)

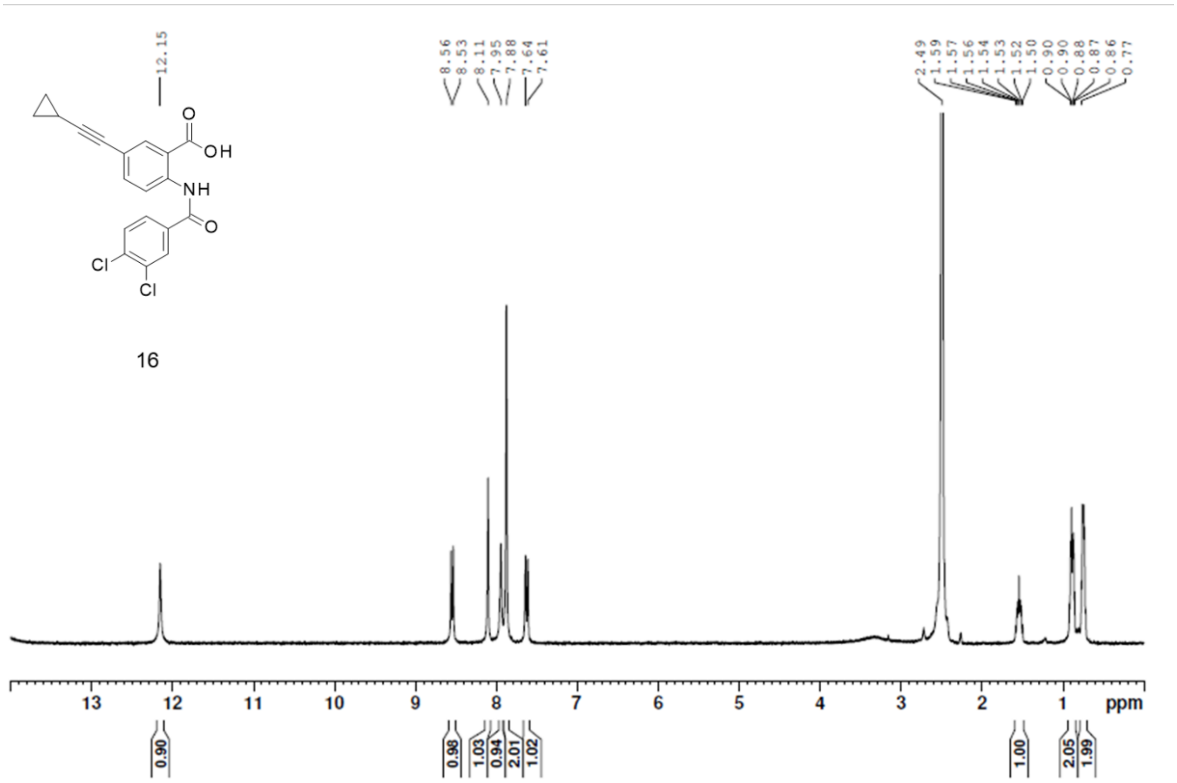

^{13}C NMR of compound 16 (75 MHz,DMSO-d6)

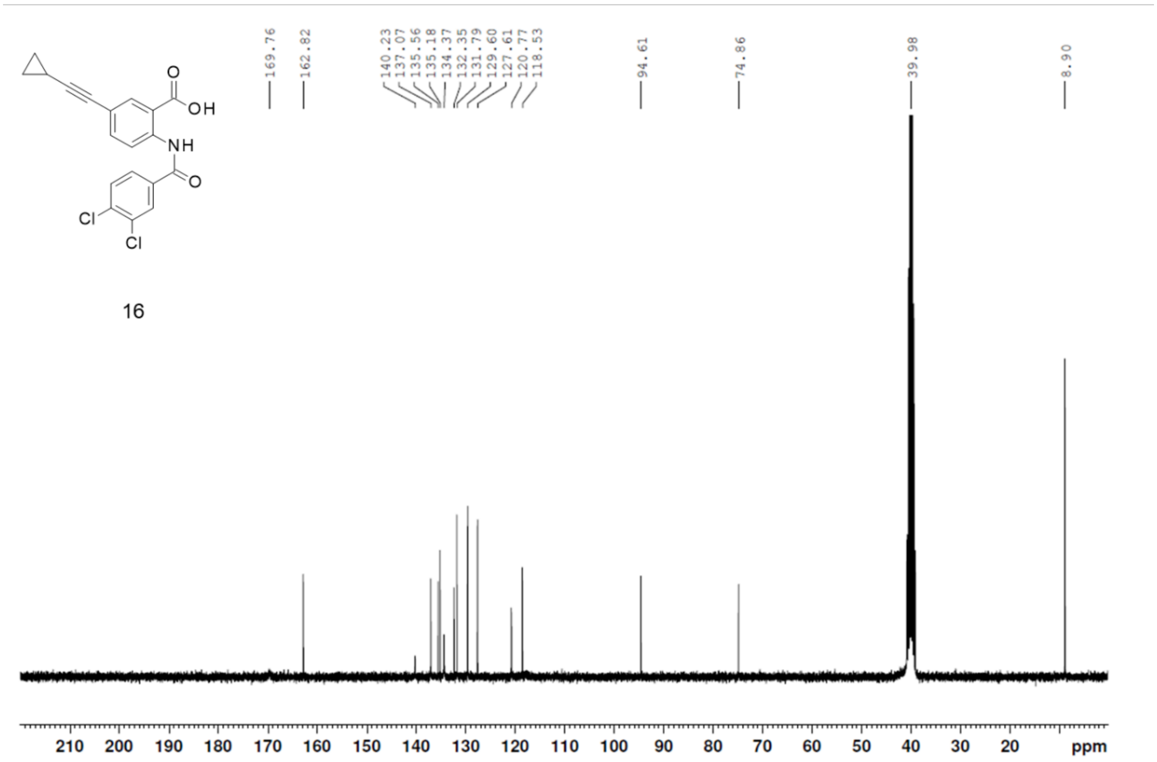

Figure S31: ^{1}H NMR and ^{13}C NMR spectra of compound **16**

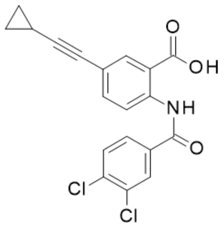

16

Elemental Composition Report **Page 1**

Single Mass Analysis
Tolerance = 20.0 PPM / DBE: min = -1.5, max = 50.0
Element prediction: Off
Number of isotope peaks used for i-FIT = 2

Monoisotopic Mass, Even Electron Ions
221 formula(e) evaluated with 5 results within limits (up to 1 best isotopic matches for each mass)
Elements Used:
C: 2-20 H: 2-36 N: 0-5 O: 0-7 Cl: 1-2

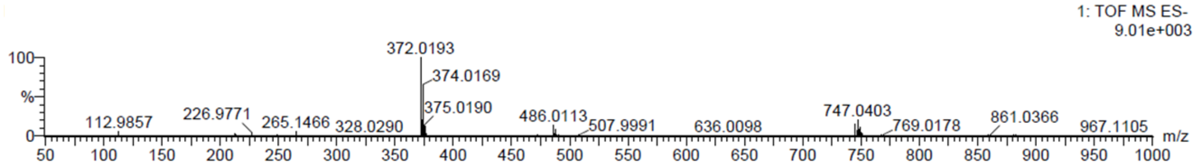

Minimum:				-1.5		
Maximum:		10.0	20.0	50.0		
Mass	Calc. Mass	mDa	PPM	DBE	i-FIT	Formula
372.0193	372.0194	-0.1	-0.3	13.5	1.1	C19 H12 N O3 Cl2

Figure S32: HRMS spectrum of compound **16**

^{1}H NMR of compound 17 (300 MHz, DMSO-d6)

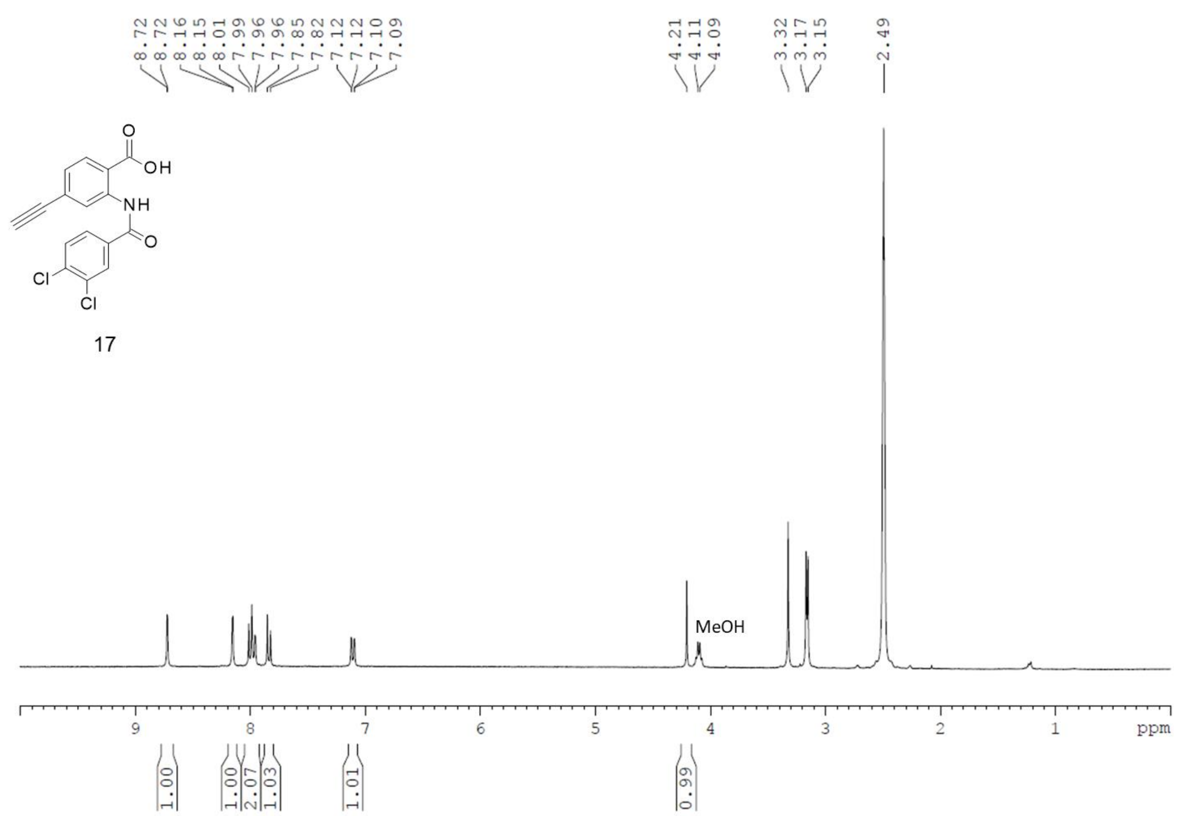

^{13}C NMR of compound 17 (75 MHz,DMSO-d6)

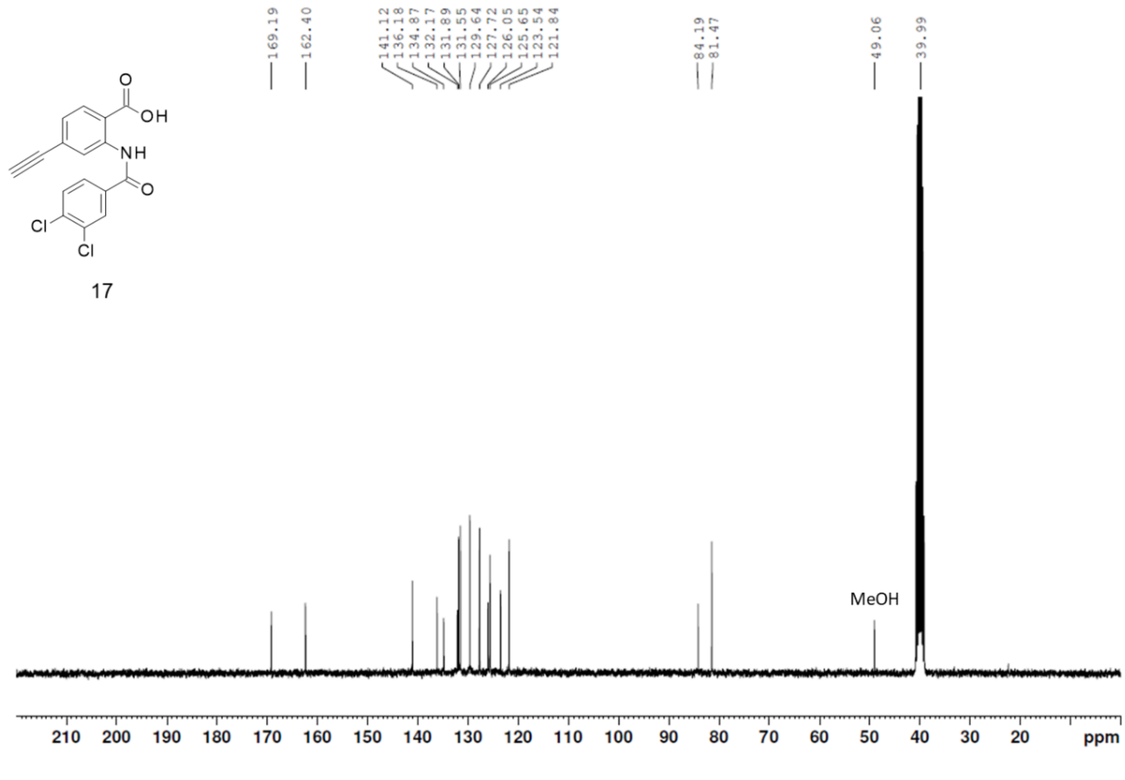

Figure S33: ^{1}H NMR and ^{13}C NMR spectra of compound **17**

Elemental Composition Report

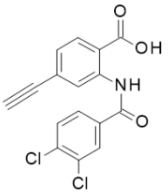

17

Page 1

Single Mass Analysis

Tolerance = 100.0 mDa / DBE: min = -1.5, max = 500.0
Element prediction: Off
Number of isotope peaks used for i-FIT = 3

Monoisotopic Mass, Even Electron Ions
350 formula(e) evaluated with 59 results within limits (up to 10 best isotopic matches for each mass)
Elements Used:
C: 0-30 H: 0-40 N: 0-4 O: 0-6 Cl: 0-2

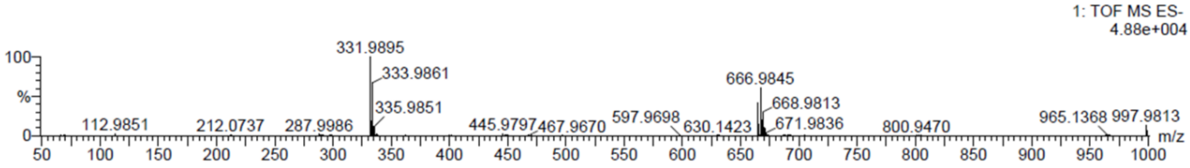

Minimum:				-1.5		
Maximum:		100.0	5.0	500.0		
Mass	Calc. Mass	mDa	PPM	DBE	i-FIT	Formula
331.9895	331.9881	1.4	4.2	12.5	9.8	C16 H8 N O3 Cl2

Figure S34: HRMS spectrum of compound **17**

^{1}H NMR of compound 18 (300 MHz, DMSO-d6)

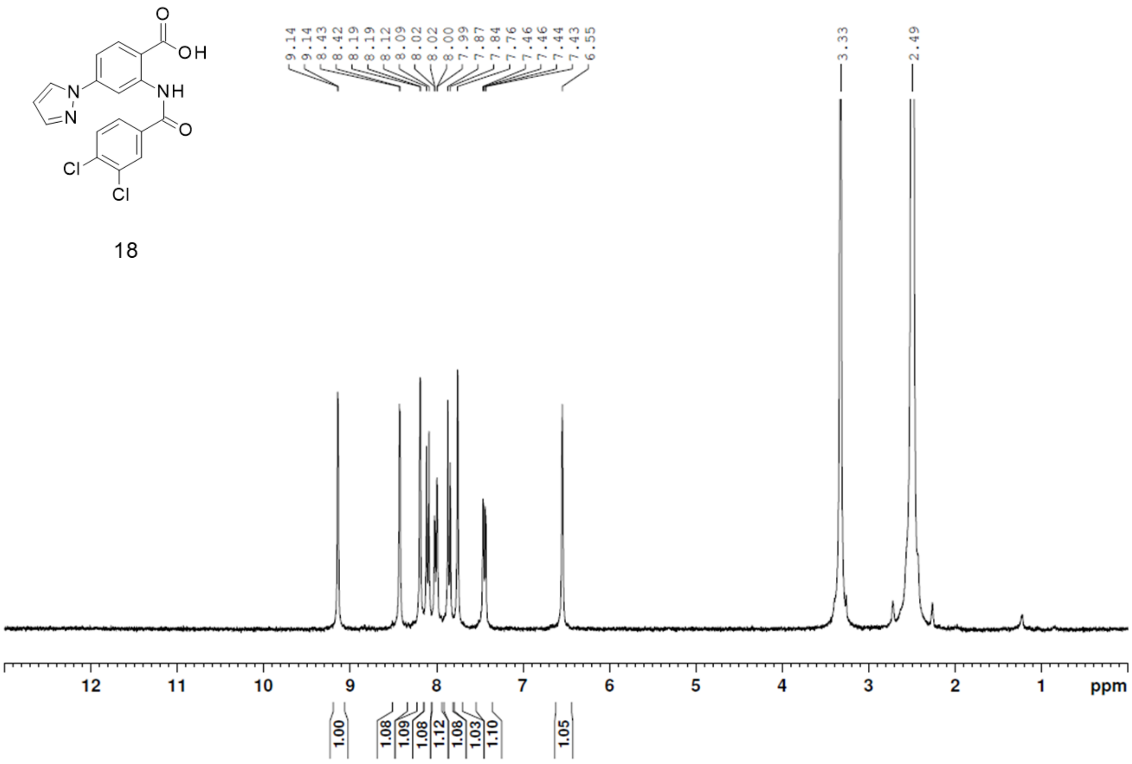

^{13}C JMOD NMR of compound 18 (75 MHz,DMSO-d6)

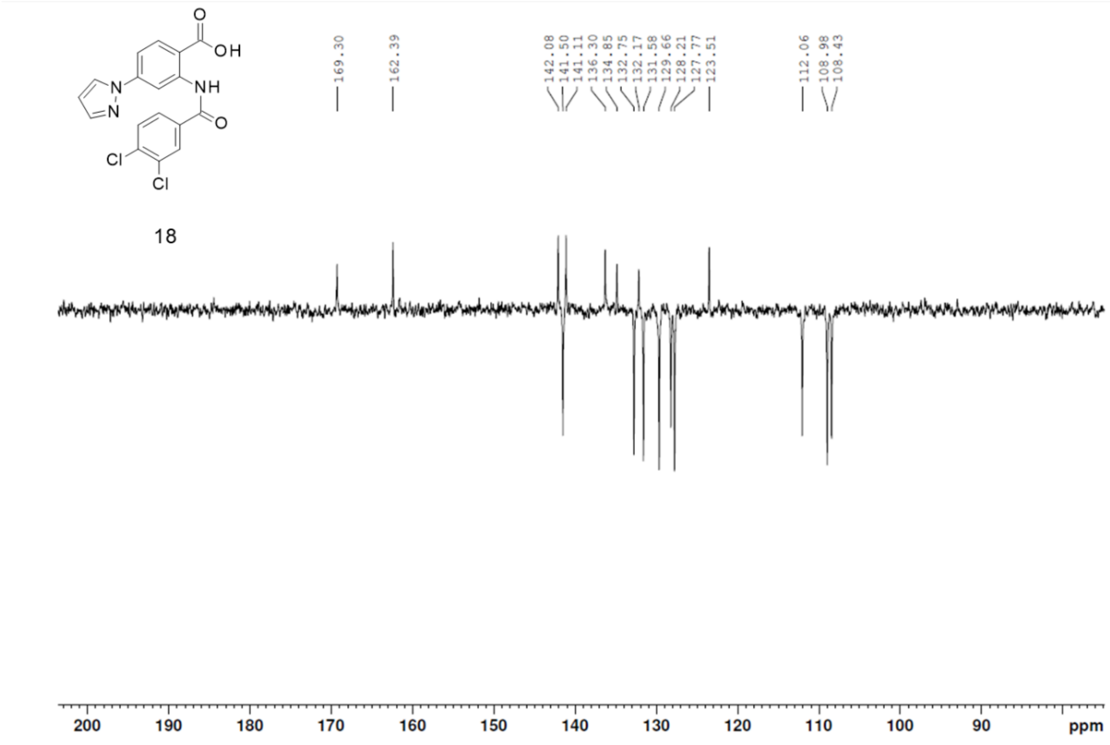

Figure S35: ^{1}H NMR and ^{13}C NMR spectra of compound **18**

Elemental Composition Report **Page 1**

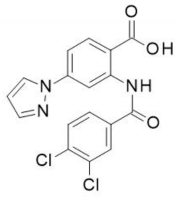

18

Single Mass Analysis

Tolerance = 10.0 PPM / DBE: min = -1.5, max = 50.0
Element prediction: Off
Number of isotope peaks used for i-FIT = 3

Monoisotopic Mass, Even Electron Ions
2596 formula(e) evaluated with 22 results within limits (up to 5 closest results for each mass)
Elements Used:
C: 10-50 H: 0-60 N: 0-10 O: 0-15 Cl: 0-2 Br: 0-1 I: 0-1

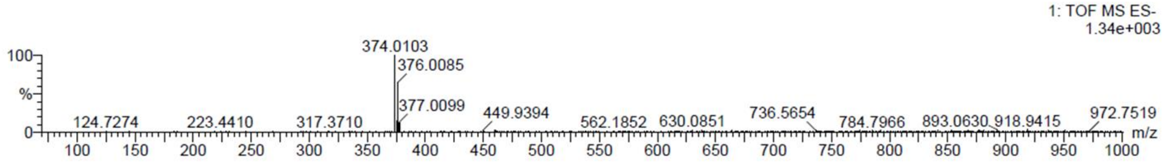

Mass	Calc. Mass	mDa	PPM	DBE	i-FIT	Formula
Minimum:				-1.5		
Maximum:		5.0	10.0	50.0		
374.0103	374.0099	0.4	1.1	13.5	9.9	C17 H10 N3 O3 Cl2

Figure S36: HRMS spectrum of compound **18**

^{1}H NMR of compound 19 (300 MHz, DMSO-d6)

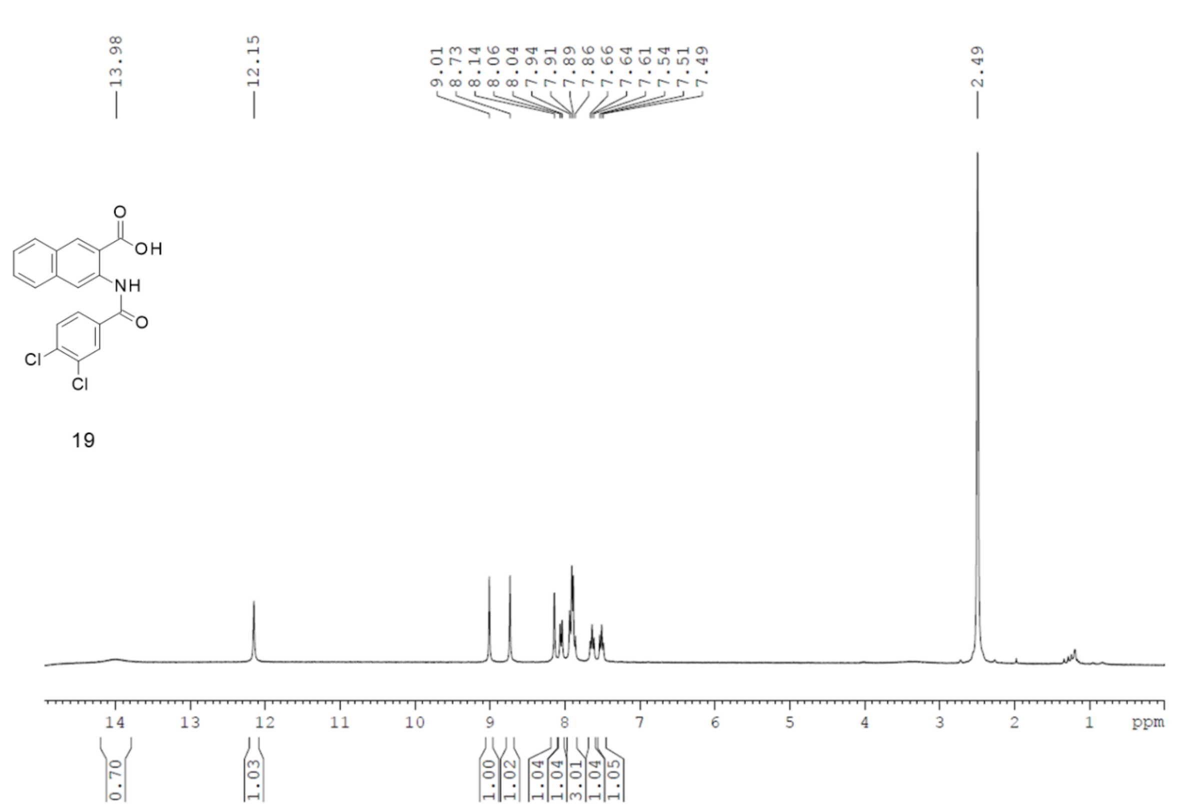

^{13}C NMR of compound 19 (75 MHz,DMSO-d6)

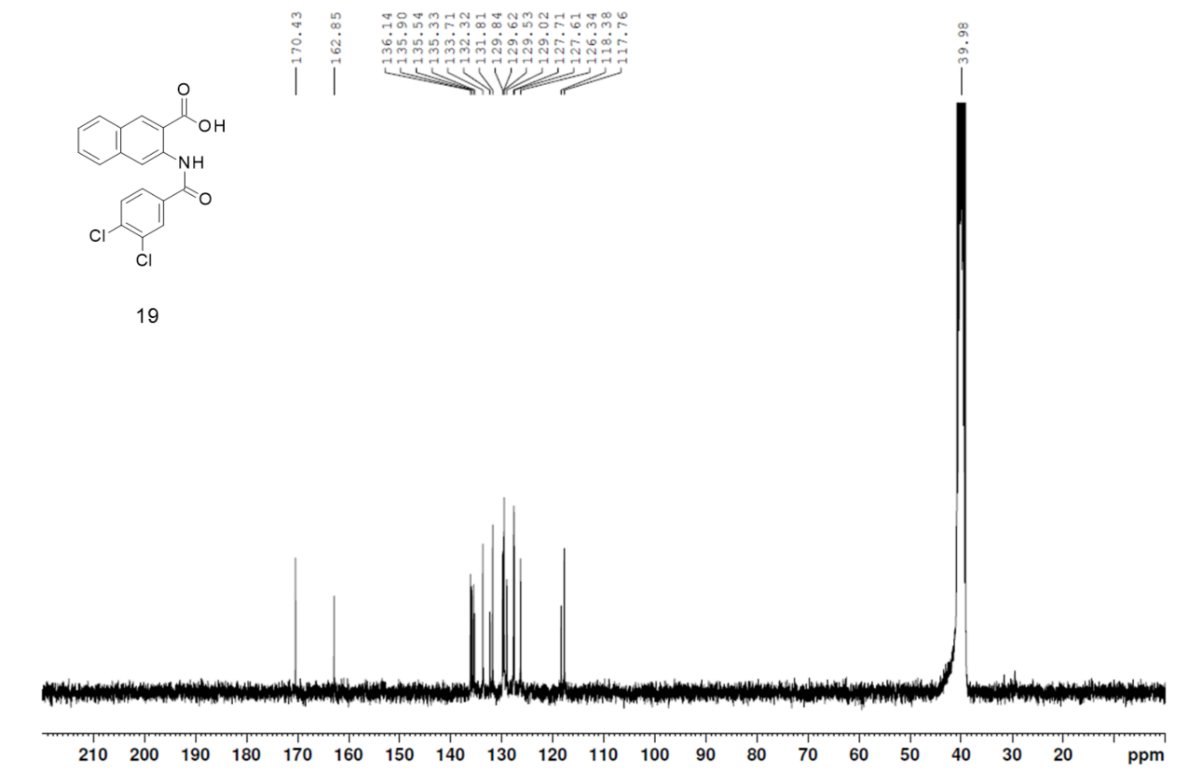

Figure S37: ^{1}H NMR and ^{13}C NMR spectra of compound **19**

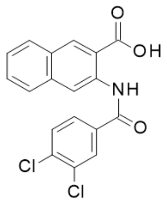

19

Elemental Composition Report

Page 1

Single Mass Analysis
Tolerance = 100.0 mDa / DBE: min = -1.5, max = 500.0
Element prediction: Off
Number of isotope peaks used for i-FIT = 3

Monoisotopic Mass, Even Electron Ions
360 formula(e) evaluated with 67 results within limits (up to 10 best isotopic matches for each mass)
Elements Used:
C: 0-30 H: 0-40 N: 0-4 O: 0-6 Cl: 0-2

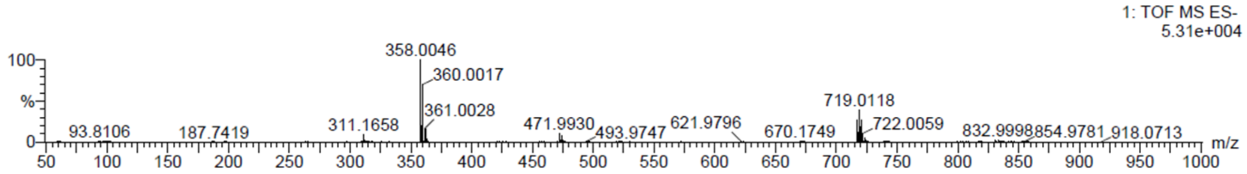

Minimum:				-1.5		
Maximum:		100.0	5.0	500.0		
Mass	Calc. Mass	mDa	PPM	DBE	i-FIT	Formula
358.0046	358.0038	0.8	2.2	13.5	54.2	C18 H10 N O3 Cl2

Figure S38: HRMS spectrum of compound **19**

^{1}H NMR of compound 20 (300 MHz, DMSO-d6)

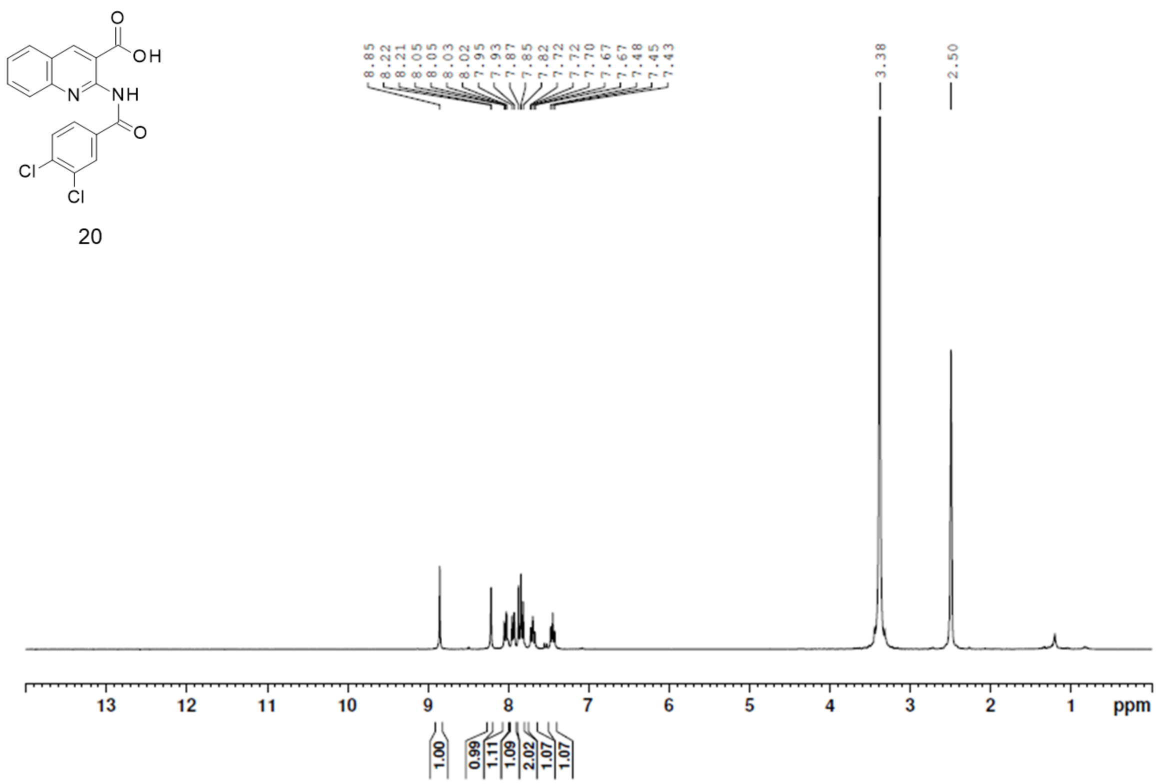

^{13}C NMR of compound 20 (75 MHz,DMSO-d6)

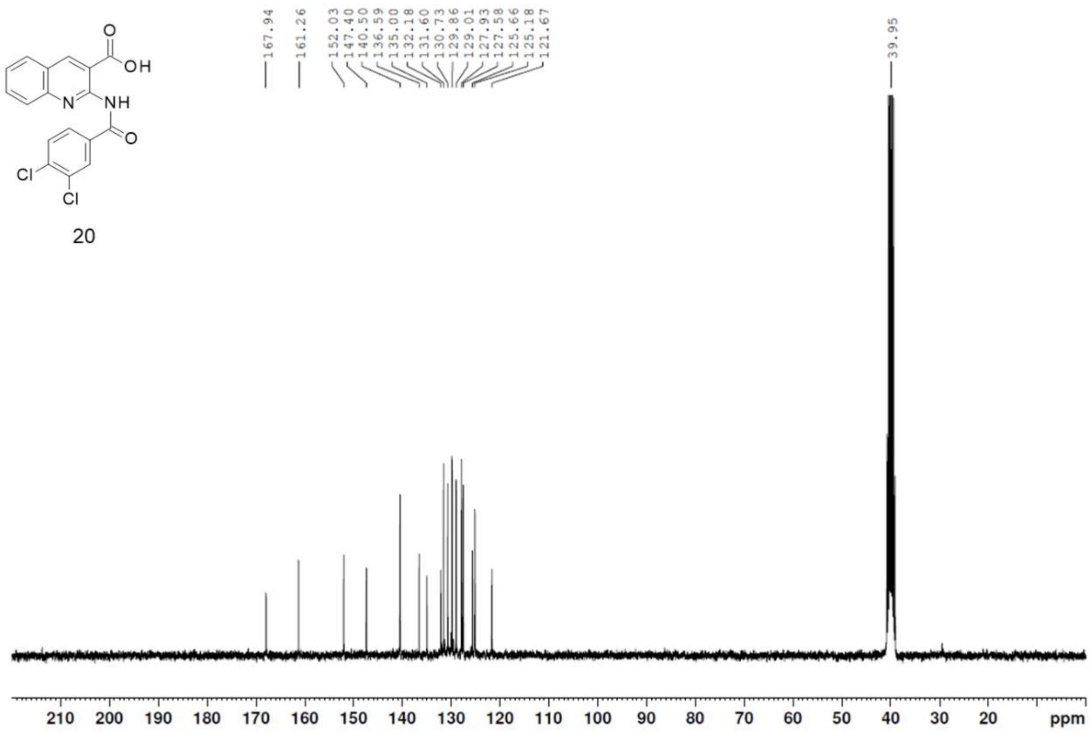

Figure S39: ^{1}H NMR and ^{13}C NMR spectra of compound **20**

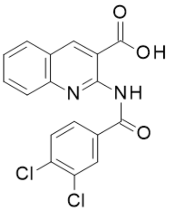

20

Elemental Composition Report

Single Mass Analysis
Tolerance = 20.0 PPM / DBE: min = -1.5, max = 50.0
Element prediction: Off
Number of isotope peaks used for i-FIT = 2

Monoisotopic Mass, Even Electron Ions
403 formula(e) evaluated with 6 results within limits (up to 1 best isotopic matches for each mass)
Elements Used:
C: 2-20 H: 2-36 N: 0-5 O: 0-7 Cl: 1-2 I: 0-1

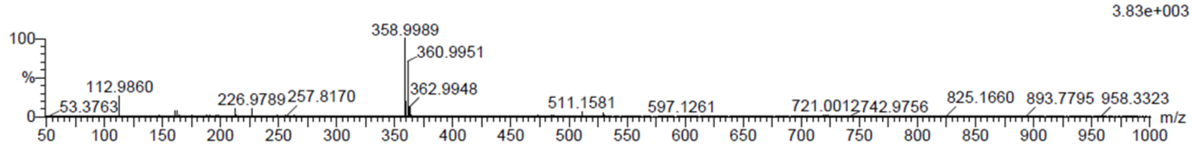

Minimum:				-1.5		
Maximum:		10.0	20.0	50.0		
Mass	Calc. Mass	mDa	PPM	DBE	i-FIT	Formula
358.9989	358.9990	-0.1	-0.3	13.5	0.0	C17 H9 N2 O3 Cl2

Figure S40: HRMS spectrum of compound **20**
